# Geschichte der Herzschrittmacher-Therapie in Deutschland

**DOI:** 10.1007/s00399-024-01010-4

**Published:** 2024-02-29

**Authors:** Bernd Lemke

**Affiliations:** Klinik für Kardiologie, Elektrophysiologie und Angiologie, Klinikum Lüdenscheid, Märkische Kliniken GmbH, Paulmannshöher Str. 14, 58515 Lüdenscheid, Deutschland

**Keywords:** Herzschrittmacherimplantation in Deutschland, Herzschrittmacherproduktion in der DDR, Elektrodenentwicklung, Batterieentwicklung, Optimale ventrikuläre Stimulation, Pacemaker implantation in Germany, Pacemaker production in the GDR, Battery development, Electrode development, Optimal ventricular pacing

## Abstract

Die Herzschrittmachertherapie beginnt bereits in der ersten Hälfte des 20. Jahrhunderts mit ersten erfolgreichen Herzstimulationen beim Menschen. Als eigentliche Geburtsstunde der heutigen Schrittmachertherapie gilt die erste vollständige Implantation eines Herzschrittmachers durch den Herzchirurgen Ake Senning am 08.10.1958 im Karolinska-Hospital in Stockholm. Die erste Herzschrittmacher-Implantation in Deutschland führte Heinz-Joachim Sykosch am 06.10.1961 in der Chirurgischen Klinik der Universität Düsseldorf durch. Zwei Jahre später erfolgte die erste Implantation in der DDR durch Friedrich Flemming am 02.09.1963 an der Charité in Ost-Berlin. Der erste in der BRD produzierte Schrittmacher kam 1963 auf den Markt, die DDR startete eine eigene Schrittmacherproduktion 1978. Im Jahr 1974 führte die Herzschrittmachertherapie in der BRD zu einer 50 %-Überlebensdauer von 6,3 Jahren im Vergleich zur medikamentösen Therapie mit < 1 Jahr. Schrittmacherelektroden haben seit der Verwendung blanker Metalldrähte eine deutlich verbesserte Qualität und Zuverlässigkeit erlangt. Einen vorläufigen Abschluss bildet hier der sondenlose Herzschrittmacher. Die Batterieentwicklung führte zu einer Flut erfinderischer Aktivitäten: wieder aufladbare Herzschrittmacher, biogalvanische Zelle, Bioenergiequellen, Nukleargeneratoren und Lithiumbatterien, die sich letztlich durchsetzten. Nach der Anfangsphase starrfrequenter Ventrikelstimulation wurden zunehmend an die Physiologie angepasste Systeme entwickelt: Bedarfsschrittmacher, vorhofbeteiligte Stimulation und frequenzadaptive Systeme. Aber erst die Rückbesinnung auf eine direkte Stimulation des Reizleitungssystems ermöglichte eine wirklich *physiologische* Stimulation des Herzens.

## Abstract (en)

Cardiac pacemaker therapy began with successful stimulation of human hearts already in the first half of the 20th century. Complete implantation of a pacemaker by the cardiac surgeon Åke Senning on October 8, 1958 at the Karolinska Hospital in Stockholm is considered the actual birth of today’s pacemaker therapy. The first pacemaker implantation in Germany was performed by Hans-Joachim Sykosch on October 6, 1961 at the Surgical Clinic of the University of Düsseldorf. Two years later, the first implantation in East Germany (GDR) was carried out by Friedrich Flemming on September 2, 1963 at the Charité in East Berlin. The first pacemaker manufactured in West Germany arrived on the market 1963; East Germany started device production in 1978. In 1974, pacemaker therapy in West Germany showed a 50% survival rate after 6.3 years compared to < 1 year with drug therapy. After initially using bare metal wires, pacemaker leads have significantly improved in both quality and reliability. Development culminated in the leadless pacemaker. Battery development led to a variety of inventions: rechargeable pacemakers, biogalvanic cells, bioenergy sources, nuclear generators and lithium batteries, the latter ultimately prevailed. In the beginning, only fixed-rate ventricular pacemakers were available. Subsequently, systems adapted to physiological requirements were developed: on-demand pacemakers, atrial-based pacing and rate-adaptive systems. However, it was not until the return to direct stimulation of the conduction system that truly *physiological* stimulation of the heart became possible.

## Internationale Vorgeschichte

Die Geschichte der Herzschrittmacher-Therapie beginnt vor ca. 100 Jahren. 1926 führte der australische Anästhesist *Mark C. Lidwell* transkutan mit einer isolierten Nadel und Netzstrom die erste erfolgreiche Herzschrittmacherstimulation bei einem Neugeborenen durch. Die Schrittmacherfrequenz war von 80–120/min variabel und die Spannung von 1,5–120 V. Die Fallbeschreibung und eine transportable Version des Stimulators stellte er auf der 3. Sitzung des Australian Medical Congress von 1929 vor^1^. Wohl aufgrund von massiven Anschuldigungen führte er keine weiteren Experimente an Menschen mehr durch.

Im Jahr 1932 stellte *Alfred Hyman* seinen externen „artificial pacemaker“ vor, der über Stimulationsnadeln den rechten Vorhof oder Ventrikel direkt reizen konnte 2. Bis März 1932 setzte er seinen Herzschrittmacher 43 Mal bei Patienten ein, in 14 Fällen mit Erfolg. Die Reaktion der Fachwelt und der Presse war verheerend. Hyman wurde vor Gericht gezerrt, man beschuldigte ihn der „frevelhaften Einmischung in die göttliche Vorsehung“. Daraufhin verzichtete Hyman auf eine Publikation seiner Reanimationsversuche bei Menschen und veröffentlichte nur die Ergebnisse von Tierversuchen. Vermutlich um *Lidwell* zu schützen, zitierte er dessen Ergebnisse nicht unter seinem Namen, sondern unter dem Namen „Gould“^3^.

Die kanadischen Herzchirurgen *John Callaghan* und *Wilfred Bigelow* versuchten 1951, während einer Hypothermie die Herzfrequenz durch Stimulation des Sinusknotens über eine Elektrode in der oberen Hohlvene zu erhöhen^4^. *Paul M. Zoll* vom Beth Israel Hospital in Boston, USA, stellte 1952 einen Elektrostimulator vor, der über thorakale Elektroden hochenergetische thorakale Impulse abgeben konnte, um damit Notfallsituationen mit Asystolie zu überbrücken^5^. Erst die Entwicklung **epimyokardialer Elektrodendrähte** durch *William L. Weirich* aus Minneapolis, USA, 1958 ermöglichte bei totalem AV-Block nach Vorhofseptumverschluss eine sichere, niederenergetische Stimulation^6^.

*Bigelow* (1950), *Zoll* (1952), *Lillehei* (1956) und *Reynolds* (1958) begannen mit der Herzstimulation unter Verwendung des *Grass*-Stimulators, der für physiologische Untersuchungen entwickelt wurde^7^. Das System war jedoch von einer externen Stromversorgung abhängig; 1957 verursachte ein Wintersturm in Minneapolis einen Stromausfall, der zum tragischen Tod eines Babys führte. *Walton Lillehei* bat daraufhin *Earl Bakken*^8^, der damals als Vertreter seiner kürzlich gegründeten Firma Medtronic das chirurgische Forschungslabor betreute, um einen batteriebetriebenen externen Herzschrittmacher zu konstruieren^9^. Nach ersten Experimenten mit einer Autobatterie griff *Bakken* schließlich auf einen damals neuartigen Stromkreislauf für Metronome zurück. Die Bauanleitung hatte er im Magazin *Popular Electronics* gefunden. Innerhalb weniger Wochen war Bakkens Herzschrittmacher einsatzbereit. Dieser **erste tragbare, batteriebetriebene Herzschrittmacher** war epochal, da hiermit die Leistungsfähigkeit dieser Therapieform demonstriert werden konnte.

Der **transvenöse Zugangsweg** zu einer endokardialen Stimulation wurde 1958 von *Seymour Furman* vom Montefiore Medical Center und Albert Einstein College of Medicine, Bronx, New York beschritten. Nachdem am 12. März 1958 im Tierversuch über die äußere Jugularvene eine rechtsventrikuläre endokardiale Stimulation erfolgreich durchgeführt wurde, konnte ein Patient mit erworbenem kompletten Herzblock und einer idioventrikulären Frequenz von 30 Schlägen/min, der eine Resektion eines Kolonkarzinoms benötigte, am 16. Juli 1958 für 2 h während der Operation erfolgreich stimuliert werden. Nach Entfernen der Stimulationselektrode kehrte der vollständige Herzblock mit idioventrikulärem Rhythmus zurück, und er starb plötzlich am 31. Juli 1958. Am 18. August 1958 wurde der zweite Patient mit Vorhofflimmern und langsamer ventrikulärer Reaktion einer Elektrodenplatzierung über die linke Basilikavene unterzogen. Die rechtsventrikuläre Stimulation wurde bis zum 21. November 1958 fortgesetzt, zu diesem Zeitpunkt war er 13 Wochen und 5 Tage kontinuierlich stimuliert worden. Er hatte sich von seinen mentalen und neurologischen Defiziten erholt und wurde mit Vorhofflimmern und einer anhaltend nicht stimulierten ventrikulären Frequenz aus dem Krankenhaus entlassen. Er hatte mehrere Rezidive, die kürzere Schrittmacherperioden erforderten, und überlebte bis zum 18. Februar 1962. Bis zum 15. Januar 1961 wurden transvenöse Stimulationen im Herzkatheterlabor unter der Leitung von *Doris Escher* bei 25 Patienten erfolgreich durchgeführt, von denen 13 mehr als 6 Monate überlebten, maximal 315 Monate. Transvenöse Elektroden wurden kombiniert mit einem externen Impulsgenerator angewandt, bis implantierbare Impulsgeneratoren verfügbar wurden^10^.

Als die eigentliche Geburtsstunde der heutigen Herzschrittmachertherapie gilt die am 08.10.1958 im Karolinska Hospital in Stockholm durchgeführte **erste vollständige Implantation eines Herzschrittmachers** mit extern wiederaufladbaren Nickel-Cadmium -Batterien durch den Herzchirurgen *Ake Senning*. Der Schrittmacher war von dem Arzt und Ingenieur *Rune Elmqvist*^11^ entwickelt und trotz Bedenken der Pioniere auf Drängen der verzweifelten Ehefrau dem Patienten *Arne Larsson* implantiert worden, der täglich 20 bis 30 Adams-Stokes-Anfälle erlitt und immer wieder reanimiert werden musste. Die Stimulation erfolgte über 2 Elektrodendrähte, deren distales Ende in das Myokard eingenäht wurde. Das System versagte innerhalb eines Tages, ein zweites hielt 8 Tage. Der Patient blieb danach 2 Jahre unversorgt, bevor 1961 die Elektroden ersetzt und ein Herzschrittmacher mit einer Quecksilber-Zink- Batterie implantiert wurde. Der Patient benötigte 5 Elektroden und 22 Impulsgeneratoren bis zu seinem Tod an einem Malignom am 28. Dezember 2001 im Alter von 86 Jahren. Der wiederaufladbare Herzschrittmacher Elema 135 wurde erfolgreich in Stockholm (1958), Uruguay (Februar 1960) und England (März 1960) implantiert, aber Elema Schonander (später Siemens-Elema) reichte nie eine Patentanmeldung ein – die Marktperspektiven wurden als schlecht wahrgenommen! Herzschrittmacher wurden als eine teure Dienstleistung für prominente Kunden mit geringem kommerziellem Wert angesehen.

Im Jahr 1960 patentierte *Wilson Greatbatch*^12^ aus Buffalo, USA, den implantierbaren Herzschrittmacher, der durch Quecksilber-Zink-Batterien betrieben wurde. *William Chardack* berichtete 1960 über die erste erfolgreiche Implantation dieses Geräts beim Menschen. Zunächst wurde am 18. April 1960 eine bipolare Elektrode epimyokardial implantiert und erst am 6. Juni 1960, bei stabiler Reizschwelle, das Schrittmacheraggregat^13^. Eine zweite Implantation erfolgte am 20.07.1960 von *Paul M. Zoll* in Boston^14^. Bis 1961 wurden von *Chardack*^15^ 15 und von *Zoll* 14 Herzschrittmacher implantiert und es wurde über eine Nachbeobachtungszeit von bis zu 12 Monaten berichtet.

## Erste Herzschrittmacher-Implantationen in Deutschland

Die erste Herzschrittmacher-Implantation in Deutschland wurde am 06.10.1961 von *Heinz-Joachim Sykosch* in der Chirurgischen Klinik der Universität **Düsseldorf** durchgeführt. Die Umstände des Eingriffs sind ausführlich von *Sven Effert*^16^ beschrieben.

Es handelte sich um ein 19-jähriges Unfallopfer, das durch eine Commotio cordis einen totalen AV-Block mit schwerem Adams-Stokes-Syndrom entwickelte und bei dem monatelang versucht wurde, mit periodischer externer Stimulation auftretende Asystolien zu überbrücken. Zunächst wurde mittels eines Trokars im 5. Interkostalraum links eine Elektrode an das Perikard vorgeführt und über Stunden stimuliert, bis wieder ein Ersatzrhythmus auftrat. Als 2 Tage später das Verfahren wiederholt werden musste, entschloss man sich zur transvenösen Applikation einer intrakardialen Elektrode, die an der Spitze eines gewöhnlichen Cournand-Katheters angebracht war und die bis in die Ausflussbahn des rechten Ventrikels vorgeführt wurde. Als die Stimulation über die Katheterelektrode wegen ansteigender Reizschwellenwerte über 80 V und Dislokation der Elektrode nicht mehr gelang und die Anfälle trotz medikamentöser Behandlung mit Adrenalin rezidivierten, wurden nach medialer Sternotomie 2 Elektroden aus rostfreiem Stahl in die rechte Ventrikelwand eingenäht, zur externen Stimulation nach außen geleitet und an einen externen Stimulator angeschlossen. Beide Elektroden brachen innerhalb eines Monats, nach einer weiteren Thorakotomie wurden zwei mit Polyäthylen isolierte Stahldrahtelektroden implantiert. Diese hielten 4 Monate und mussten wegen Sondenbruchs und einer Infektion des Drahts entfernt werden. Inzwischen verfügte die Klinik über einen externen Stimulator, der über thorakale Elektroden mit einer Spannung von 12 V das Herz stimulieren konnte. Wegen der schmerzhaften Mitstimulation der Thoraxmuskulatur konnte die Stimulation jeweils nur für Minuten durchgeführt werden. Nachdem der Patient für 3 Monate mit einem Ersatzrhythmus von 26 bis 28 min^−1^ stabil war, traten erneut anhaltende Asystolie-Anfälle auf. „In dieser Situation entschlossen wir uns zur Implantation eines Mikroschrittmachers [von Chardack] mit Elektroden aus einer Platinlegierung … Die Operation fand am 6. Oktober 1961 in Intratrachealnarkose statt … Unter permanenter elektrischer Stimulation wurde der Herzbeutel eröffnet, … die beiden Elektroden in einem Abstand von ca. 2 cm an der Vorderwand der linken Kammer angenäht, … der Schrittmacher in einer Tasche der Bauchmuskulatur versenkt. Die Aktivierung der Kammern durch den implantierten Mikroschrittmacher erfolgte unmittelbar nach dem Anschluss der Elektroden.“^17^

**Abb. 1:**
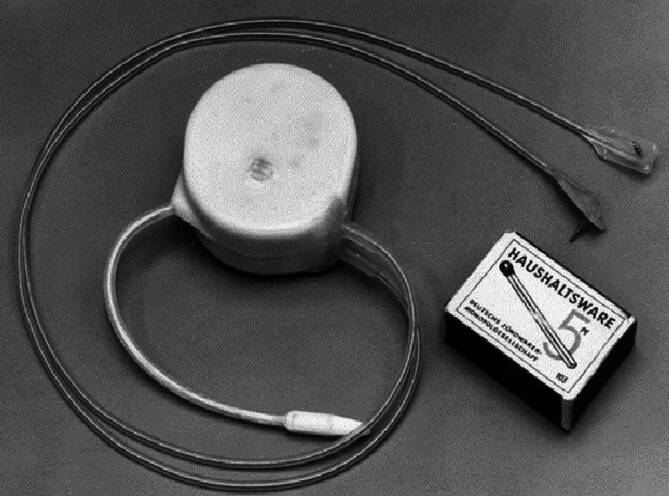
Greatbatch-Schrittmacher mit epimyokardialen Elektroden. (Quelle: Prof. Joachim Winter, Düsseldorf)

Über die Beschaffung des Geräts (Abb. 1) gibt es unterschiedliche Darstellungen.

In einem „Zeitzeugen-Interview“ mit *Berndt Lüderitz*^18^ gab *Effert* an, auf Einladung *Chardacks* 1960 in Buffalo erstmalig den implantierbaren Herzschrittmacher gesehen zu haben. Er beschloss, nach Rücksprache mit seinem Chef, der die Kosten übernahm [sic!], den Herzschrittmacher in der rechten Hosentasche nach Deutschland einzuschmuggeln. *Sykosch* widerspricht in einer persönlichen Mitteilung dieser „nice story“. Die unsterilen Transportbedingungen und die äußerst brüchigen Platin-Iridium-Elektroden hätten eine sichere Beförderung in der Hosentasche nicht zugelassen. Aber auch die Darstellung von *Sykosch*, er habe nach Kontaktaufnahme mit *Chardack* und *Greatbatch* die persönliche Bestellung des Herzschrittmachers veranlasst, entspricht nicht den Aktenunterlagen. Demnach handelte es sich um eine „ordnungsgemäße“ Bestellung des Geräts durch *Effert* im Namen der 1. Medizinischen Klinik bei der Firma Heinrich G. Ulrich, die den Vertrieb in Deutschland für die Firma Medtronic übernommen hatte und einen Hinweis auf die „Eilbedürftigkeit der Beschaffung, insbesondere im Hinblick auf den Behandlungsfall“ enthält (Faksimile der Bestellung vom 12.09.1961)^19^. Von der Erstimplantation eines aufladbaren Herzschrittmachersystems in Schweden hatten sowohl *Sykosch* als auch *Effert* keine Informationen, da *Senning* nur auf einem französischen Kongress für Medical Electronics über die Implantation berichtet hatte. 1976 konnte *Effert* dazu beitragen, dass *Elmqvist* und *Senning* der renommierte Aachen-Münchener Preis für Technik und angewandte Naturwissenschaften verliehen wurde.

Auch um die Durchführung der Operation ranken sich manche Gerüchte.

Im „Zeitzeugen-Interview“ mit *Lüderitz* berichtet *Effert*: „Am 6. Oktober 1961 haben wir in Düsseldorf implantiert. *Jochen Sykosch* – Herzchirurg, mit dem ich lange Jahre zusammengearbeitet habe – war der Operateur.“ Dem widerspricht *Sykosch*, nach dessen Angaben er, in verschworener Gemeinschaft aus OP-Personal‘ an einem Samstag früh morgens um sieben Uhr vor Eintreffen seiner Assistentenkollegen und ohne Kenntnis seines Chefs in der Klinik operiert habe. (Persönliche Mitteilungen (2012) Prof. *Heinz-Joachim Sykosch*, Düsseldorf).

*Sykosch* selbst stellt die Umstände und Konsequenzen des Eingriffs wie folgt dar: „Mein Chef hatte gesagt, ich soll den Mann in Frieden sterben lassen. Aber ich hatte von der Entwicklung eines erstmals vollständig implantierbaren Schrittmachers in den USA gehört. Das Gerät habe ich bekommen und es hat funktioniert.“ Da er sich damit über die Anweisungen seines Chefs, Prof. *Ernst Derra* hinweggesetzt hatte, sei ihm gekündigt worden, er wurde aber bald darauf wieder eingestellt^20^. Der von *Sykosch* operierte Patient wurde 45 Jahre alt – er starb an einem Nierenleiden.

Am 13.12.1961 führte *Paul Sunder-Plaßmann* in **Münster** bei einem Patienten mit totalem AV-Block die zweite Herzschrittmacher-Implantation in Deutschland durch. Die epimyokardial implantierten bipolaren Platinelektroden wurden nach Tunnelung mit einem abdominal implantierten System verbunden. Elf Monate später berichtete er darüber beim Deutschen Thoraxchirurgenkongress^21^,^22^. Dem Patienten ging es sehr gut, es trat kein Adams-Stokes-Anfall mehr auf.

Die Implantationszahlen waren im Folgejahr noch gering. So wurden 1962 in Düsseldorf nur 6 weitere Patienten^23^ und in Münster sogar nur ein weiterer Fall versorgt^24^. 1963 implantierte *Sebastian Bücherl* in **Berlin (West)** den ersten in Deutschland von dem Physiker *Max Schaldach*^25^ und dem Elektroingenieur *Otto Franke* entwickelten Herzschrittmacher.

Die erste Herzschrittmacher-Implantation in der **DDR** wurde am 02.09.1963 von *Friedrich Flemming* an der **Charité in Ost-Berlin** durchgeführt, gefolgt von Operationen am 17.10. und 31.10.1963 durch *Gerd Kuhlgatz* in **Rostock** und am 13.11.1963 durch *Martin Herbst* und *Wolfgang Ursinus* in **Leipzig**^26^.

Bei der ersten Patientin handelte es sich um eine 30-jährige Frau, die wegen rezidivierender Synkopen und Verdacht auf eine idiopathische Herzmuskelhypertrophie im Januar 1963 an der **Charité** eine Angiokardiographie erhielt. Bei der retrograden Passage des Katheters in den linken Ventrikel trat eine Asystolie auf, die durch präkordiale Faustschläge unterbrochen und in eine Kammerautomatie von 35 min^−1^ überführt werden konnte. Unter einer Isoproterenol (Novodrin®)-Medikation blieb die Patientin bis Mai 1963 anfallsfrei. Danach häuften sich Adams-Stokes-Anfälle und die Patientin konnte nur unter einer Orciprenalin-Tropfinfusion frei von Anfällen gehalten werden. Dies veranlasste dazu, die Implantation eines elektrischen Herzschrittmachers am 02.09.1963 in der Chirurgischen Klinik der Charité vorzunehmen. Die Patientin war danach bei einer Pulsfrequenz von 90 min^−1^ auch ohne Einnahme von Medikamenten beschwerdefrei.

Der zweite Patient war ein 63-jähriger Mann mit Gelenkrheumatismus und rezidivierender Bewusstlosigkeit. Im Januar 1963 nahmen die Anfälle zu. Durch lautes Schreien und kräftige Faustschläge auf die Brust konnte der Patient diese Anfälle vermeiden. Die Pulsfrequenz betrug 40 min^−1^ , und im EKG konnte ein 2:1- AV-Block registriert werden. Der Patient wurde auf Isoprenalin-Tabletten eingestellt. Ende August 1963 verstärkten sich die Anfälle derart, dass Anfallsfreiheit nur unter Orciprenalin-Gaben zu erreichen war. Die Pulsfrequenz schwankte zwischen 40 und 20 min^−1^. Aus diesem Grund wurde am 07.11.1963 die Implantation eines elektrischen Herzschrittmachers vorgenommen. Der Patient konnte danach beschwerdefrei entlassen werden. Am 08.01.1964 traten erneut Adams-Stokes-Anfälle auf. Als Ursache stellte sich ein Bruch der Platinelektrode heraus, die gegen die Stahldrahtelektrode ausgetauscht wurde. Eine weitere Implantation erfolgte am 18.11.1963 bei einem 59-jährigen Patienten mit totalem AV-Block und Adams-Stokes-Anfällen. Auch hier trat nach 37 Tagen ein Bruch des Überleitungsdrahts auf, der in Lokalanästhesie ausgewechselt wurde^27^.

Der ersten Herzschrittmacher-Implantation in **Rostock** im Oktober 1963 ging der tragische Fall einer 39-jährigen Frau voraus, die bei AV-Block III mit Asystolie am 27.01.1963 per Thorakotomie eine ventrikuläre Myokardelektrode implantiert bekam und an einen batteriebetriebenen externen Schrittmacher tschechischer Bauart angeschlossen wurde. Nach 12 Wochen kam es zum Reizschwellenanstieg mit Ausfall der Stimulation und Tod der Patientin^28^.

Protagonisten der Herzschrittmachertherapie an der Universität Rostock im Jahr 1963 waren Oberärzte der Medizinischen und der Chirurgischen Klinik, der Internist *Gerhart Hafemeister* und der Chirurg *Gerd Kuhlgatz*. Das erste vollständig implantierte Gerät erhielt am 17.10.1963 eine 54-jährige Patientin wegen zunehmender Adams-Stokes-Anfälle bei totalem AV-Block. Die Operation wurde über eine linksseitige posterolaterale Thorakotomie vorgenommen und das Aggregat im linken Oberbauch subkutan implantiert. Bis zum Jahresende 1963 wurden in Rostock noch zwei weitere Patienten versorgt.

In **Leipzig** erhielt am 13.11.1963 ein 71-jähriger Mann mit gehäuften Adams-Stokes-Anfällen den ersten Herzschrittmacher. Durch Umschaltung der Frequenz von 70 auf 90/min wurden die anfänglich noch auftretenden Spontanreaktionen unterdrückt. Im November und Dezember 1963 wurden in Leipzig noch zwei weitere Patienten mit totalem AV-Block versorgt, eine 40-jährige Frau nach toxisch-entzündlicher Schädigung des Erregungsleitungssystems und eine 19-jährige Frau nach kardiopulmonaler Reanimation^29^.

Für die ersten Operationen in der DDR stand der Schrittmacher der Firma Vitatron, Niederlande, zur Verfügung, der an der Universität Groningen entwickelt wurde. Seine Abmessungen betrugen 9,2 × 4,4 × 2,2 cm, sein Gewicht ca. 150 g, 6 Quecksilberbatterien lieferten eine Spannung von 8 V. Über einen transistorgesteuerten Impulsgenerator wurden Rechteckimpulse von 2 ms Dauer generiert. Um das Gehäuse aus Silikon war die indifferente Elektrode als schmaler Metallstreifen angebracht. Die Stimulation erfolgte unipolar über einen isolierten Platinstift, der mit dem isolierten Edelstahlkabel verbunden war. Eine Reserveelektrode war als Drahtelektrode konstruiert, die durch den Herzmuskel durchgeführt wurde. Sie kam zum Einsatz bei einem Bruch der Plantinelektrode, die dann in Lokalanästhesie gegen die Stahldrahtelektrode ausgetauscht werden konnte. Der starrfrequente Herzschrittmacher konnte postoperativ per Magnet, der in Längsrichtung des Schrittmachers um 180^o^ gedreht wurde, auf zwei Frequenzen (65 und 85 min^−1^) eingestellt werden. Neben 7 Aggregaten der Firma Vitatron wurden bis 1967 noch 40 Herzschrittmacher der Firma Devices Sales Ltd. (entwickelt am St. Georges Hospital in London, England), 6 P-Wellen-gesteuerte Atricor-Pacemaker der Firma Cordis, USA, und 3 Schrittmacher der Firma Elema, Schweden, implantiert.

Von Oktober 1963 bis März 1965 erhielten in **Rostock** insgesamt 15 Patienten einen Herzschrittmacher. Bei einer Nachbeobachtung bis zu 17,5 Monaten wurden ein Bruch eines Elektrodenkabels und technische Komplikationen mit kontinuierlichem Frequenzabfall und Ausfall der Schrittmacherfunktion bei 4 Patienten beobachtet. Eine Patientin starb 3 Wochen nach der Operation an einer Hirnembolie. Bei allen anderen Patienten führte die Schrittmacherbehandlung zur Anfallsfreiheit und deutlichen Verbesserung der körperlichen Leistungsfähigkeit^30^.

Über den Krankheitsverlauf der ersten Rostocker Patientin berichtete *Georg H. von Knorre* 2013^31^, der die Bandbreite der in den Anfängen dieser Therapie häufigen Komplikationen illustriert. Bereits 6 Monate nach der Implantation musste sich die Patientin einer ersten Revisionsoperation wegen eines Kabelbruchs unterziehen, bei der die gebrochene Elektrode gegen die Ersatzelektrode am Gerät ausgetauscht wurde. Insgesamt erfolgten 17 Re-Eingriffe, dennoch konnte die Patientin ein adäquates Leben führen, bis sie 80-jährig am 24.01.1990 nach über 26 Jahren mit einem Herzschrittmacher verstarb.

## Ergebnisse der Herzschrittmachertherapie in Deutschland

**Abb. 2:**
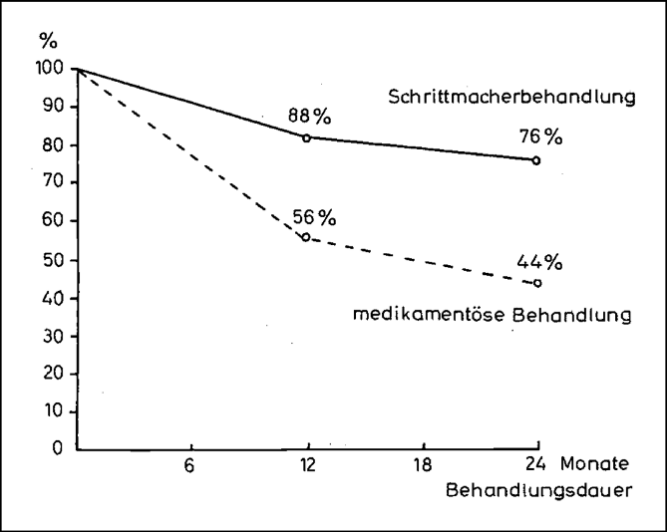
Überlebensrate nach Feststellung eines kompletten atrioventrikulären Blocks in Abhängigkeit von der Therapie. Untere, *gestrichelte Linie*: medikamentöser Behandlung nach Literaturangaben. Obere, *durchgezogene* *Linie*: nach Schrittmacherbehandlung. (© Georg Thieme Verlag KG, mit freundl. Genehmigung. Sykosch J, Büchner M, Effert S. Sechs Jahre Schrittmachertherapie. DMW 1968; 93:777–784)

In der **Bundesrepublik** stellte *Sykosch* aus **Düsseldorf** 1968 die Ergebnisse von 6 Jahren Herzschrittmachertherapie vor. Eindrucksvoll konnte gezeigt werden, dass die Überlebensrate nach 1 und 2 Jahren deutlich über der bei medikamentöser Behandlung lag (Abb. 2).

Bei 256 von 372 Patienten lag ein kompletter atrioventrikulärer Block vor, 91 % Adams-Stockes-Anfälle, 88 % der Patienten waren älter als 50, 31 % älter als 70 Jahre. Das Verhältnis Männer zu Frauen betrug 2:1. Bei den ersten 102 Patienten wurden überwiegend Chardack-Elektroden nach Thorakotomie epimyokardial, in der Regel am linken Ventrikel appliziert. Bei 11 Patienten wurden die Elektroden mittels Pericardiotomia inferior am rechten Ventrikel fixiert. Bei 259 Fällen wurde die intrakardiale Elektrodentechnik angewendet: Einführen eines Elektrodenkatheters durch eine Vene (anfangs Venae sectio der rechten Vena jugularis, später Vena cephalica), subkutane Verbindung des Katheters zum Schrittmacher rechts subpektoral.

Bei 90 % der Patienten wurden Herzschrittmacher mit fixer Frequenz verwandt. Zwölf Patienten (3 %) erhielten einen P‑Wellen-gesteuerten Schrittmacher, bei 2 Patienten trat eine vom Schrittmacher auf die Kammern übertragene Vorhoftachykardie auf. 7 % der Patienten erhielten einen Demand-Schrittmacher.

Die Problematik der Herzschrittmachertherapie bestand in der relativ hohen Komplikationsrate. Diese lag zum einen an vorzeitigem Batterieversagen oder Ausfall elektronischer Bauelemente. Bei der ersten Serie von 1961 bis 1964 musste der Schrittmacher nach 13,8 Monaten ausgetauscht werden, danach wurde mit einer mittleren einwandfreien Funktion von 2 Jahren gerechnet.

Eine zweite typische Komplikation waren Elektrodenbrüche, die bei Verwendung von epimyokardial applizierten Elektroden in 21 % auftraten, im Gegensatz zu 2,5 % bei transvenös platzierten Elektroden. Dafür kam es bei diesen in 8,5 % zur Verlagerung der Elektrodenspitze, die revidiert werden musste. Bei 8 Patienten (3 %) traten Ventrikelperforationen auf, die durch Zurückziehen der Elektrodenspitze in allen Fällen [!] ohne weitere Folgen korrigiert werden konnten. Infektionen der Schrittmacherkapsel und Perforationen des Herzschrittmachers traten in 8 % nur bei den epimyokardialen Elektroden auf, Hautperforationen der Elektroden nur bei den über die V. jugularis applizierten Sonden^32^.

Die Ergebnisse von 15 Jahren Herzschrittmachertherapie stellten *Effert* und *Irnich*^33^ aus **Aachen** 1974 vor. Ende 1972 lebten in der Bundesrepublik etwa 18.000 Herzschrittmacherpatienten, 2 Jahre später waren es geschätzt bereits 30.000. Seit 1969 stieg die Zahl der lebenden Patienten exponentiell an, wobei sich die Zahl alle 1,6 Jahre verdoppelte. Der Nutzen der Herzschrittmachertherapie ergab sich vor allem aus der Überlebensrate nach Erstimplantation. Bei einem Implantationsalter von 66,9 Jahren betrug die 50 %-Überlebensdauer 6,3 Jahre und lag damit um knapp 6 Jahre unter der mittleren Lebenserwartung. Demgegenüber betrug bei medikamentöser Behandlung die 50 %-Überlebensrate weniger als 1 Jahr. Der Vergleich der Implantationszahlen mit anderen Ländern ergab 1972, dass wahrscheinlich Schweden mit 350 pro Million Einwohner die meisten Herzschrittmacherpatienten hatte. Bei der Rate der Neuimplantationen lagen Schweden mit 123, USA und Kanada mit geschätzt 75 bis 143 und die Bundesrepublik mit 130 gleich auf. Von den östlichen Staaten war die DDR mit 122 Herzschrittmacherpatienten und 39 Neuimplantationen pro Million Einwohner das Land mit der größten Schrittmacherdichte, noch vor England mit 73 und 22 Patienten und Japan mit 6 und 1,2 Patienten. 1974 musste man davon ausgehen, dass die Herzschrittmachertherapie in weiten Teilen der Welt noch keinen Eingang gefunden hatte, weniger aufgrund mangelnder Kenntnis der Herzschrittmachertherapie als wegen Kostenproblemen.

Nach einer Umfrage des Bundesgesundheitsamtes betrug die mittlere Funktionsdauer eines Herzschrittmachers in der Bundesrepublik im Mittel 23,2 Monate. Bei DM 3400 für eine Schrittmacherbehandlung einschließlich 8‑tägigem Krankenhausaufenthalt ergaben sich 1974 Behandlungskosten von 52,7 Mio. DM pro Jahr. Wären alle Herzschrittmacher so kontrolliert worden, dass sie bis zur tatsächlichen Batterieerschöpfung belassen worden wären, hätten sich Funktionszeiten von über 30 Monaten ergeben. Leider wurden die Kontrollen von den Krankenkassen nicht bezahlt, so dass ein großer Teil der Herzschrittmacher prophylaktisch ausgetauscht wurde.

Richtungsweisend waren auch die von *Effert* und *Irnich* gegebenen Hinweise zur optimalen Herzschrittmachertherapie. Für die AV-Blockierungen wäre der vorhofgesteuerte Herzschrittmacher hämodynamisch gesehen am vorteilhaftesten, bei binodaler Erkrankung die sequenzielle Stimulation von Vorhof und Kammer und bei isolierter Sinusbradykardie der synchronisierte Vorhofschrittmacher. Nur bei Vorhofflimmern und bei intermittierenden AV-Blockierungen wäre der kammersynchrone Schrittmacher geeignet. Allerdings machten 1974 vorhofbeteiligte Systeme gegenüber dem theoretischen Bedarf von 77 % nur 1 % aus. Dies beruhte in erster Linie auf der Schwierigkeit, eine Vorhofelektrode sicher zu platzieren. Bisher war dazu die Thorakotomie unerlässlich. Die Ergebnisse der in Deutschland entwickelten Hakenelektrode waren aber ermutigend^34^ (siehe Kap. „Vorhofbeteiligte Stimulation“).

Über die Ergebnisse von 10 Jahren Therapie mit Herzschrittmachern an der **Charité Berlin, DDR,** berichtete 1973 *Lothar Dressler* von der I. Medizinischen Klinik. Von September 1963 bis April 1973 wurden insgesamt 700 Patienten mit einem Herzschrittmacher versorgt. Das Durchschnittsalter betrug 65,9 Jahre (Männer: 65,7; Frauen: 66,2 Jahre); der jüngste Patient war 14 Tage, der älteste 86 Jahre alt.

Die Implantationstechnik hatte sich im Verlauf der 10 Jahre gewandelt; während in den ersten 4 Jahren überwiegend eine Thorakotomie zur Elektrodenfixation erforderlich war, wurde dieses Verfahren in den späteren Jahren zugunsten der transvenösen intrakardialen Elektrodenimplantation fast völlig verlassen. Schon 1967 betrug der Anteil der Patienten mit transvenöser Implantation 50 %. In 80,7 % der Fälle wurde die V. cephalica als Zugangsvene benutzt und das Aggregat in einer Subkutantasche platziert. Drucknekrosen der Haut traten bei 7 % der Patienten auf und eine Primärinfektion bei 1,2 %.

In den ersten Jahren standen Brüche der Elektroden im Vordergrund. Eine wesentliche Verbesserung wurde mit Einführung der Elgiloy-Spiralen erreicht. Seit 1967 wurden bei Erst- und Reimplantationen fast 700 transvenöse Biotronik-Elektroden dieser Technologie verwandt. Ein Elektrodenbruch trat nur bei 5 Patienten auf (0,7 %). Durch die Elastizität der Elektroden war eine Ventrikelperforation sehr selten (2 Patienten). Auch eine Reizschwellenerhöhung mit Exit-Block trat nur bei 3 Patienten auf. Das Hauptproblem, die Sondendislokation, konnte durch die Einführung der Kragenelektrode (Biotronik IE 60) von 30 % auf 7 % reduziert werden (siehe Abschn. „Endokardiale Stimulation“).

Ab 1968 wurden ausschließlich Biotronik-Schrittmacher eingesetzt. Für die Serie IP‑3 (starrfrequent) lag die Funktionsdauer bei 25,8 Monaten, für die Serie IRP‑3 (R-synchron) bei 24,1 Monaten. Die seit 1969 verwendeten Serien IP-44 und IRP-44 erreichten 36,5 und 29 Monate. Die Ausfallrate durch technischen Fehler betrug 3,1 % und 6,3 %. Die seit 1970 implantierten 59 Demand-Schrittmacher IDP-44 wiesen bei kurzer Nachbeobachtungszeit keine technischen Defekte auf. Bei den vorhofgesteuerten Schrittmachern (IVP‑3, IVP-44 und IVP-54) konnte eine Beurteilung wegen der kleinen Fallzahlen (14 Patienten seit 1967) nicht erfolgen^35^ (siehe Kap. „Batterieentwicklung“).

Für die **DDR** stellte *Joachim Witte* von der I. Medizinischen Klinik der **Charité Berlin** 1973 „Stand und Tendenz der Versorgung mit Herzschrittmachern“ vor. In den Jahren 1968 bis 1972 wurden an 8 Zentren 2771 Patienten versorgt; die Neuimplantationsrate lag bei 44 Herzschrittmachern pro Million Einwohner. Immer häufiger wurden R‑Zacken gesteuerte Schrittmacher eingesetzt. Unter ökonomischen Gesichtspunkten und wegen des notwendigen Imports (doppelter Preis der gesteuerten gegenüber den festfrequenten Schrittmachern) stellten 31 % starrfrequente Systeme in Berlin gegenüber 61 % in Leipzig ein vertretbares aber extremes Minimum dar. Auch die noch unzureichende Ausrüstung kleinerer Einrichtungen mit geeigneten externen Herzschrittmachern und Elektroden für die zeitweilige Stimulation stellte ein Problem dar. An der Entwicklung einer flexiblen Einschwemmkatheterelektrode, die ohne Röntgendurchleuchtung gelegt werden konnte, wurde in Zusammenarbeit mit der Industrie gearbeitet^36^.

Durch die **Einführung eines Herzschrittmacher-Dokumentationssystems** 1975 in der DDR, an dem sich alle [!] implantierenden Kliniken beteiligten, lagen 1977 erstmals exakte Angaben über die zwischen 1963 und 1976 versorgten 9242 Patienten vor. Durch die exponentielle Entwicklung nahm die Neuimplantationsrate auf 173 pro Million Einwohner zu. Die klinische Indikation bestand bei 67,7 % der Patienten in einem Adams-Stokes-Syndrom, bei 5,5 % in einer schweren Bradykardie ohne Bewusstlosigkeit und bei 8,2 % in einer bradykarden Herzinsuffizienz. Eine prophylaktische Schrittmacher-Implantation wurde bei 2,3 % der Patienten vorgenommen, bei 16,3 % lagen andere Gründe vor. Die häufigste EKG-Indikation war mit 41 % der permanente AV-Block III^o^, zuzüglich des permanenten AV-Blocks II^o^ mit 4,9 % und der intermittierenden AV-Blockierungen mit 29,4 %. Erst in den letzten Jahren nahm der Anteil der supraventrikulären Bradykardien auf 11,8 % zu und das Bradykardie-Tachykardie-Syndrom auf 3,3 %. Parallel dem zunehmenden Anteil intermittierender Bradykardien kam es zu einer kontinuierlichen Abnahme des Anteils festfrequenter Systeme auf unter 40 %. Anfang der 60er-Jahre stand als einzige Alternative die vorhofgesteuerte Version mit myokardialen Elektroden zur Verfügung. Im Zusammenhang mit der Entwicklung der transvenösen Implantationstechnik fanden die ventrikelgesteuerten Systeme eine schnelle Verbreitung und verdrängten die vorhofgesteuerte Version weitestgehend^37^.

Während 1969 die Herzschrittmacherimplantation in 8 Zentren durchgeführt wurde, gab es 1975 bereits 19, 1977 dann 24 und 1989 schließlich 36 implantierende Kliniken. Die Neuimplantationsrate nahm auf 222 pro Million Einwohner zu, parallel dazu erhöhte sich der Anteil supraventrikulärer Bradykardie auf 26,4 % und der Bradyarrhythmia absoluta auf 5,9 %. Zusammen mit dem Bemühen, die chirurgische Intervention durch Lokalanästhesie, kleine Herzschrittmacher und Punktionstechnik über die V. subclavia zu minimieren, wurde in 11 der Zentren die Operation zunehmend von Kardiologen durchgeführt^38^.

### Aufbau einer eigenen Herzschrittmacherproduktion in der DDR

„Industriell in der DDR hergestellte Herzschrittmacher-Elektroden sind über Versuche nie hinausgekommen. Anfangs wurden die Herzschrittmacher mit Biotronik-Elektroden implantiert. Später wurden überwiegend unipolare Kragenelektroden der tschechischen Firma TESLA verwendet. Es gab auch bipolare Tesla-Elektroden in der DDR. Die waren aber nicht sehr beliebt, obwohl wir natürlich wussten, dass bipolare Elektroden in den USA bevorzugt wurden. Die bipolaren Tesla-Elektroden waren ziemlich anfällig: Es gab vermehrt Elektrodenprobleme und -ausfälle, auch Isolationsdefekte selbst an mechanisch gar nicht so sehr beanspruchten Stellen. Und man konnte defekte Elektroden nicht reparieren. Eine unipolare Elektrode konnte man abschneiden, dann wurde eine Schraube hineingedreht, und sie hielt gleich noch einmal so lange. Solche Bastelarbeiten waren ja in einem chronisch klammen Land überlebenswichtig. Eingeführt wurden die Elektroden via Venae sectio der V. cephalica, dann auch über (selbstgebaute!) Einführungsbestecke. Selbstgebaut aus den Schutzhüllen von Vygon Swan-Ganz-Kathetern (!!!). Bastelarbeit …“^39^

Zwischen der Charité und der Firma Biotronik gab es in den 60er-Jahren eine Zusammenarbeit bei der Entwicklung und Erprobung neuartiger Elektroden (siehe Kap. „Vorhofbeteiligte Stimulation“) und Stromversorgungssysteme (siehe Kap. „Batterieentwicklung“). Im Zusammenhang mit der aus wirtschaftlichen Gründen angestrebten Herzschrittmacher-Importablösung wurden 1971 mit Biotronik Lizenzverhandlungen geführt, die im April 1972 von Biotronik abgebrochen wurden. Eine von Biotronik ausgehende Wiederaufnahme der Verhandlungen wurde 1974 aus politischen Gründen abgelehnt. Parallel dazu wurden 1972 Lizenzverhandlungen mit Medtronic aufgenommen, die im April 1973 wegen fehlender wirtschaftlicher Tragfähigkeit abgebrochen wurden. Die Kosten für die Herzschrittmacher-Importe beliefen sich 1975 auf 6 Mio. DM^40^.

Initiator einer eigenständigen Herzschrittmacherentwicklung in der DDR war der Neurologe *Rudolf Richwien*, Oberarzt an der Neurochirurgischen Abteilung der Chirurgischen Uniklinik **Halle**^41^. Als Hobbyelektroniker und Funkamateur entwickelte er die Schaltungen und Steuerungen und war Inhaber mehrerer Patente sowie Autor mehrerer Studien zur Konstruktion elektronischer Herzschrittmacher^42^. Im Frühjahr 1971 gab es erste Kontakte zwischen *Richwien* und der Geschäftsleitung der Privatfirma Wagomat Halle (Entwicklungsleiter Dipl.-Physiker *Kurt-Bernd Otte*^43^) über den Vorschlag von *Richwien*, implantierbare Herzschrittmacher in der DDR selbst herzustellen.

Es ist das Verdienst der 1973 gegründeten Arbeitsgruppe „Herzschrittmachertherapie“ und ihres langjährigen Leiters *Joachim Witte* von der Charité **Berlin**, der beim MfG^44^ die notwendigen Mittel für Importe und zur Entwicklung der DDR-Schrittmacher- und Elektrodenindustrie mobilisieren konnte, dass die Herzschrittmacherimplantationen bereits zu Beginn der 80er-Jahre international empfohlene Zahlen erreichten.^45^ Er lehnte die parallel zur Eigenentwicklung bestehenden Bestrebungen einer Lizenznahme als wirtschaftlichen Irrweg ab und setzte sich damit massiven Anfeindungen aus. Nach einem Porträt im Neuen Deutschland vom 4./5. Januar 1986 wurde „der Gedanke an Eigenentwicklung und Produktion per Gutachten verworfen, als Vermessenheit, ja Spinnerei abgetan.“^46^ Als 1973 die Entwicklungsarbeiten wegen fehlender Bauelemente stockten, schlug er vor, in der Zwischenzeit externe Herzschrittmacher für den Notfalleinsatz zu entwickeln und herzustellen. Da diese Schrittmacher komplett mit DDR-Bauelemente und Materialien hergestellt werden konnten, wurde die Entwicklung und Produktionsaufnahme Mitte 1974 abgeschlossen. Das vollständige Gerätesystem für die temporäre Elektrostimulation („extracard“, „minicard“ und Elektrodenkatheter) wurde auf der Leipziger Herbstmesse 1974 ausgestellt und mit einer Goldmedaille ausgezeichnet^47^.

Seit 1978 wurden im Ausland hergestellte Herzschrittmacher schrittweise durch Aggregate eigener Herstellung (VEB TUR Dresden/Ultraschalltechnik Halle) ersetzt. Der Anteil importierter Aggregate ging 1987 auf 4 % zurück. Von den Herzschrittmachern der zweiten Generation wurden seit 1983 mehr als 34.000 in der DDR und im Ausland implantiert.

Die 1. Generation (1978–1981) besaß als Stromquelle Quecksilberoxid-Zink-Elemente der Firma Mallory. Elektronik und Batterien wurden als Epoxydharz-Verguss von einem Edelstahlgehäuse ummantelt. Zur Verfügung standen ein starrfrequentes und zwei getriggerte Modelle mit unterschiedlicher Impulsbreite für die Kammerstimulation, Gewicht 160–175 g. Diese wurden Anfang der 1980er-Jahre durch Geräte der 2. Generation abgelöst. Als Energiequelle dienten bereits ab 1982 im sächsischen Betrieb Fahrzeugelektrik Pirna hergestellte Lithium-Jod-Batterien (2,8 V; 2,5 Ah). Kern der Elektronik war ein an der TU Karl-Marx-Stadt (heute Chemnitz), konzipierter und im Zentrum Mikroelektronik Dresden hergestellter Schaltkreis mit 1200 Transistoren mit der damals aufkommenden CMOS-Technik. Batterie und Schaltung waren hermetisch abgeschlossen in einem Titangehäuse. Die Geräte der „MCP-Serie“ und der „LCP-Serie“ (Longlife Cardiac Pacemaker) umfassten 3 Systeme: Ventrikel-Demand-Schrittmacher (VVI)^48^ in zwei Frequenzvarianten mit 74 und 55 min^−1^ (Anteil 1987: 91,2 %), Vorhof-Demand-Schrittmacher (AAI; 3,6 %), vorhofgesteuerter Ventrikelschrittmacher (VAT; 2,0 %) mit einem Gewicht von 59 g und einer erwarteten Lebensdauer von 12 Jahren. Die 2. Generation stellte bis 1989 den Hauptanteil der in der DDR implantierten Herzschrittmacher dar. Bis zu 6000 Herzschrittmacher jährlich wurden nach Ungarn, Polen und Bulgarien exportiert. Außerdem lief in China eine Lizenzproduktion an.

Mitte der 1980er-Jahre begann die Entwicklung der 3. Generation. Dabei handelte es sich um die multiprogrammierbaren 1‑ und 2‑Kammer-Schrittmacher „Reficard“ und „Reficard duo“ mit integriertem CMOS-Schaltkreis und 12.000 Transistoren, der eine bidirektionale Datenübertragung ermöglichte. Ab Februar 1989 gingen erste Fertigungsmuster für die klinische Erprobung an die Kliniken. Die Serienproduktion startete im August 1989. Nach der Wiedervereinigung 1990 ging der Betrieb in Halle an die Firma Medtronic. Die Geräte der 3. Generation wurden zunächst als „Billiglinien“ weiter angeboten, aber kaum noch gekauft und die Produktion eingestellt. Den Herstellerbetrieb der Lithiumbatterien in Pirna und die Schaltkreisentwickler aus Chemnitz übernahm die Firma Biotronik.

Zu den Herzschrittmachern der 2. Generation gehören Geräte, die durch ungewöhnlich lange Laufzeiten auffielen. So informierte die Mitteldeutsche Zeitung am 22.12.2012, dass in der Helios Klinik Eisleben ein LCP 201 nach 25 Jahren Laufzeit, vermutlich aus Sicherheitsüberlegungen, gewechselt wurde^49^. Im Herzzentrum Leipzig staunten die Ärzte 2013 über einen 1987 im St. Georg-Krankenhaus implantierten DDR-Schrittmacher, der sogar noch einige Jahre funktioniert hätte und gegen ein Defibrillatorsystem ausgetauscht werden musste^50^. Über eine in der kardiologischen Praxis Dr. J. Placke, Rostock, kontrollierte Patientin mit LCP 255, der wegen intermittierender Bradykardien implantiert wurde und nach 30 Jahren immer noch funktionierte, berichtete die Bützower Zeitung am 25.01.2017^51^.

Unter den technologischen Faktoren wirken sich Batteriekapazität, Selbstentladung der Batterie und Leerlaufstrom der Schaltung auf die Funktionsdauer eines Herzschrittmachers aus. Laut Prospekt betrug der Leerlaufstrom 0,01 mA. Der Eigenstromverbrauch des CMOS-Schaltkreises der LCP-Serie war hauptsächlich so niedrig, weil wesentliche Funktionen in einem einzigen Halbleiterchip integriert waren und weil – aus heutiger Sicht – simple Schaltungen verwendet wurden^52^.

## Elektrodenentwicklung und Zugangsweg

Seit der Verwendung blanker Metalldrähte aus rostfreiem Stahl, die durch die Brustwand mittels Thorakotomie in das linksventrikuläre Epimyokard eingeführt wurden, haben die aktuellen implantierbaren Schrittmacherelektroden eine deutlich verbesserte Qualität und Zuverlässigkeit erlangt. Einen vorläufigen Abschluss bildet hier der sondenlose Herzschrittmacher.

### Epimyokardiale Stimulation

Eine entscheidende Verbesserung in der Haltbarkeit epimyokardialer Stimulationsdrähte war die von dem Chirurgen *Samuel Hunter* zusammen mit dem Medtronic-Chefingenieur *Norman Roth* entwickelte bipolare Elektrode. Sie bestand aus 2 Edelstahlleitern, die mit einem Silikonkautschukgemisch zur Isolation ummantelt waren, und einer dornartigen Sondenspitze, die eine stabile Verankerung im Myokard ermöglichen sollte^53^. Am 4. April 1959 wurde dem ersten von 3 Patienten diese Elektrode in das linksventrikuläre Myokard implantiert, durch die Brustwand ausgeleitet und mit dem batteriebetriebenen externen Pulsgenerator von Medtronic verbunden. Der Patient überlebte trotz mehrerer Stimulationsausfälle und Notfallrevisionen 7 Jahre mit dem externen Schrittmachersystem^54^.

Eine elastische Elektrode zur Reduktion von Leiterbrüchen wurde 1959 von der Firma Elema Schönander (später Siemens-Elema) zusammen mit der Telefongesellschaft Ericsson entwickelt. Ein Leiterdraht aus rostfreiem Stahl wurde in vier einzelnen Bändern „koaxial“ um ein Polyestergeflecht gewickelt und zusätzlich von einer äußeren Isolation aus weichem Polyethylen umhüllt. Die unipolare epikardiale Stimulationselektrode bestand aus einer Platinscheibe mit einem Durchmesser von 8 mm^55^.

Im weiteren Verlauf konzentrierte sich die Entwicklung auf die Modifikation des Elektrodenkopfes und der myokardialen Kontaktfläche, sowie unterschiedlicher Fixationskonzepte mit aufnähbaren (suture-on) oder einschraubbaren (screw-in) Elektroden. Allerdings zeigten die Elektroden immer wieder starke Reizschwellenanstiege, die zum Funktionsverlust mit notwendigen Revisionseingriffen und folglich zu einer äußerst eingeschränkten Akzeptanz führten. Ursache waren zumeist lokale Ödem‑, Blutungs- und Inflammationsreaktionen, die oftmals durch die gewebstraumatisierenden Elektrodenköpfe selbst induziert wurden. Ein Fortschritt ergab sich schließlich durch die Einführung steroidfreisetzender Kontaktflächen, die diese lokalen Reaktionen dämpften und zu elektrisch stabileren Messwerten führten^56^.

In **Deutschland** konnte *Heiko Burger*^57^ aus **Bad Nauheim** 2012 in einer retrospektiven Analyse bei 130 konsekutiven Patienten über einen Zeitraum von 48 Monaten zeigen, dass die Implantation epikardialer bipolarer und steroidfreisetzender Elektroden sicher ist und eine sehr geringe Komplikationsrate aufweist. Keines der technischen Elektrodenkonzepte (einschraubbar vs. annähbar) erwies sich als überlegen, alle epikardialen Elektroden zeigten eine hervorragende Langzeitleistung und Haltbarkeit. Sie stellen deshalb, insbesondere für die kardiale Resynchronisationstherapie, eine gute Alternative zu transvenösen Elektroden dar, wenn diese nicht zu implantieren sind oder bei Trikuspidalklappenersatz vermieden werden sollen.

### Endokardiale Stimulation

In den frühen 60er-Jahren wurde die endokardiale Sondenplatzierung mit implantierbaren Herzschrittmachern kombiniert und entwickelte sich zum Standard in der Schrittmachertherapie. Anfang 1962 ließ *Hans Lagergren* in Stockholm eine unipolare Elektrode für die permanente intrakardiale Stimulation entwickeln, und am 5. Juni 1962 wurde erstmalig eine endokardiale Elektrode an einen implantierbaren Impulsgenerator angeschlossen^58^. Bis zum 1. April 1965 wurde sie bei 100 aufeinanderfolgenden Patienten implantiert, mit einer maximalen Beobachtungszeit von 3 Jahren.

Die intrakardiale Elektrode (EM 588, Siemens Elema, Stockholm, Schweden) war extrem weich und flexibel, hatte einen Durchmesser von 1,2 mm und war etwa 1,5 m lang. Sie bestand aus einem gesponnenen Textilkern, um den vier dünne Streifen aus Edelstahl gewickelt waren und eine isolierende Umhüllung aus Polyethylen. Die Elektrodenspitze bildete ein zylindrischer Platinknopf, 10 mm lang und 3 mm im Durchmesser (später 6 mm/2,5 mm). Sie hatte kein Lumen, wurde über einen Führungskatheter appliziert und in die richtige Position im Herzen „eingeschwemmt“.

Die Elektrode wurde vorzugsweise über die freigelegte äußere oder innere Jugularvene auf der rechten Seite eingeführt und ihre Spitze unter Röntgenkontrolle in der Apexregion des rechten Ventrikels verankert. Sie wurde in Schlaufen im Nackengewebe vernäht und der Rest des Kabels subkutan hinter dem Schlüsselbein über Brust und Bauch bis zur rechten Leistengegend gezogen. Die Elektroden wurden mit einem externen, batteriebetriebenen Herzschrittmacher verbunden, der in einem Gurt über der Schulter getragen wurde.

Wenn der Herzschrittmacher für etwa 2 Monate funktionierte, blieb der Patient entweder weiterhin an einem externen Gerät oder die Kabel wurden an der Stelle, wo sie steril im Unterhautgewebe lagen, durchtrennt und es wurde ein subkutaner Herzschrittmacher implantiert. Angesichts der hohen Defekthäufigkeit der bis dahin verfügbaren Systeme wurde dieser nur bei 25 Patienten implantiert, während die restlichen 75 Patienten die ganze Zeit über ein externes Gerät trugen. Da der neue subkutane Herzschrittmacher von Siemens-Elema zuverlässiger war, sollte frühzeitiger implantiert werden, insbesondere um Elektrodenverschiebungen bei Verwendung eines externen Schrittmachers zu vermeiden^58^. 1979 berichteten *Lagergren* und *Edhag* über die 10-Jahres Langzeitdaten bei 306 Patienten. Bei 62 % traten im Verlauf keine Sondenprobleme auf und die Stimulationsreizschwelle blieb bei 2,2 V ± 0,8 V stabil. Bei den 46 Patienten mit Sondenkomplikationen lag die Dislokation mit 55 % an 1. Stelle, gefolgt vom Reizschwellenanstieg (32 %) und Sondeninfektionen (13 %). Ein Elektrodenbruch trat nur bei 2 Elektroden auf, die wegen eines Twiddler-Syndroms revidiert werden mussten. Dies wurde auf das flexible Design der Elektrode und die Technik, die Elektrode in die richtige Position im Herzen „einzuschwemmen“ zurückgeführt^59^.

In den USA etablierte *Victor Parsonnet* aus Newark dieses Verfahren am 6. Oktober 1962^60^. Ein vollständig transvenös implantierbares System wurde 1965 von *Chardack* und Medtronic, USA, kommerziell eingeführt. 1966 berichtete *Chardack* über 31 Patienten, die mit einer neuen bipolaren endokardialen Elektrode behandelt wurden. Das Design bestand aus zwei Spiralen aus rostfreiem Stahl, die sich in getrennten Lumina eines Silikonkautschukschlauchs bewegten und mit der Elektrodenspitze aus Platin verschweißt waren. Die zum Einführen und Manipulieren der Elektrode erforderliche Steifheit und leichte Krümmung wurde durch Edelstahlstilette erreicht, die in das Lumen der Spiralen eingesetzt wurden^61^.

#### Probleme der Elektrodenentwicklung

Zu den Hauptproblemen, die in den frühen Jahren der Elektrodenentwicklung auftraten, gehörten Dislokationen, Myokardperforationen, Leiterbrüche, Isolationsdefekte und Austrittsblockaden (hohe Reizschwellen).

#### Sondenstabilität

Historisch gesehen war das häufigste klinische Problem die Dislokation oder myokardiale Penetration, die in einigen Serien bis zu 30 % oder mehr betrug. Daher wurden in den 1970er-Jahren zur stabileren Elektrodenlage Fixationsmechanismen entwickelt. Hierbei erhielten die Sondenköpfe kleine Ankerflügelchen („tines“) zur passiven oder korkenzieherartige Schrauben („helix“) zur aktiven Fixation. Anfangs wurden die aktiv fixierbaren Elektroden überwiegend im Vorhof eingesetzt. Da die modernen Steroid-eluierenden Schraubelektroden mit mikroporösem Stimulationskopf sich kaum in der Reizschwelle und Wahrnehmung von atraumatischen Elektroden unterscheiden, werden sie heute überwiegend auch im Ventrikel eingesetzt. Mit ihnen erreicht man bei einer Dislokationsrate von unter 1 % eine hohe Stabilität, erhält mehr Auswahlmöglichkeiten für die Implantationsstelle und kann die Elektrode bei Bedarf auch leichter wieder extrahieren (s. Kapitel „Vorhofbeteiligte Stimulation“).

#### Elektrodenleiter

Nachdem in den 1960er-Jahren die blanken Metallelektroden eine unzuverlässige Korrosionsbeständigkeit gezeigt hatten, wurden sie zugunsten von Metalllegierungen wie Elgiloy^62^ oder MP-35N^63^ ersetzt. Zur Reduktion der Sondenbrüche wurden die einwendligen Leiterdrähte Ende der 70er-Jahre durch mehrwendlige Elektroden abgelöst. Diese waren entweder koradial oder koaxial konfiguriert, was eine größere Biegungsstabilität ermöglichte.

In **Deutschland** berichtete 1987 *Eckhard Alt* aus **München** über 2563 implantierte Elektroden mit einer Gesamtbeobachtungszeit von 8558 Jahren. Sondenbrüche traten in 3,9 % auf, entsprechend 1,2 % pro Patientenjahr. Elektrodenbrüche waren mit 7 % bei den einwendligen Elektroden signifikant häufiger (2,0 % pro Patientenjahr) als bei der Siemens Elema 588 Elektrode ohne Innenlumen mit 2,5 % (0,5 % pro Patientenjahr) und bei den mehrwendligen Elektroden mit 1 % (0,7 % pro Patientenjahr). Bei Patienten mit AV-Block 2. oder 3. Grades konnte bei Verwendung einer mehrwendligen Elektrode eine signifikant bessere Überlebensrate nachgewiesen werden. Deshalb sollten einwendlige Elektroden nicht mehr implantiert und bei schrittmacherabhängigen Patienten ausgetauscht werden^64^.

#### Isolation und Polarität

Die frühesten Elektroden verwendeten eine Teflon- oder Polyethylenisolierung, die bald durch Silikon und Polyurethan ersetzt wurde. Silikon hatte sich als zuverlässig erwiesen, erforderte aber wegen geringer Zug‑, Dehnungs- und Reißfestigkeit eine dickere Isolation. Polyurethan, ein Polymer, zeigte eine höhere Festigkeit und weniger Abrieb als Silikon, wodurch die Isolierung dünner ausfallen konnte. Polyurethanelektroden wurden seit 1977 beim Menschen implantiert. Aufgrund von Fällen von Spannungsrissen („environmental stress cracking“, ESC), die 1981 festgestellt wurden, wurden Änderungen im Herstellungsprozess vorgenommen. *Kenneth Stokes*, Director of Brady Leads Research, Medtronic, berichtete 1986 über eine jährliche Versagensrate von 0,3−0,7 % der meisten Medtronic Polyurethan-Elektroden mit Ausnahme der Elektrode 6972 mit einer Rate von 4,1 %^65^. Demgegenüber berichtete *David Hayes* von der Mayo Medical School in Rochester, USA, noch 1992 über eine 6‑Jahres-Ausfallrate der Medtronic 4012 Elektrode von 21,2 %. Alle Ausfälle waren mit isolierungsbedingtem Versagen vereinbar^66^.

Im dänischen Herzschrittmacherregister beobachtete *Mogens Møller* aus Odense, Dänemark, 1996 einen signifikanten Unterschied zwischen der Zuverlässigkeit von unipolaren und bipolaren Elektroden. Nach 10 Jahren zeigten die 17.823 unipolaren Elektroden eine Zuverlässigkeit von 97,0 %, die 9827 bipolaren Elektroden dagegen nur eine von 75,5 %. Drei verschiedene Elektrodenmodelle mit Polyurethan-80A-Innenisolierung waren die Hauptursache für die verringerte Zuverlässigkeit von bipolaren Elektroden. Als Erklärung wurde eine Metallionen-Oxidation der Polyurethanschicht zwischen Innen- und Außenleiter angenommen. Für die bipolaren Elektroden Medtronic 4012, Telectronics 284 und Siemens 1010T, 105T und 1050T wurde eine sorgfältige Überwachung und ein angemessen indizierter Austausch gefordert^67^.

Nach dem Fehlergipfel der Polyurethanelektroden in den frühen 1990er-Jahren, einem Auslieferungsstopp für die verdächtigten Sonden und umfangreichen Bauartänderungen zeigten jüngere Daten aus Dänemark^68^ und Überwachungsprogramme der betroffenen Hersteller kaum noch Unterschiede in den Überlebensraten zwischen uni- und bipolaren Elektroden.

Zur Verbesserung der Zuverlässigkeit des Isolationsmaterials wurden Copolymere aus Silikon und Polyurethan (SPC) entwickelt (St. Jude/Abbott), die eine hohe Biostabilität und Flexibilität aufwiesen^69^. Neue Isoliermaterialien wie Ethylen-Fluor-Ethylen (ETFE) und beschichtete Drahttechnologien ermöglichten sehr schlanke (5 Fr) mehrpolige Elektroden mit einer einzelnen radialen Leiterspule (Intermedics). Polyurethan wurde nur noch für die Handhabungseigenschaften aufgebracht. Nach *Willem de Voogt* aus Amsterdam, Niederlande, waren 1999 die mittelfristigen Zuverlässigkeitsergebnisse mit 99 % nach 5 Jahren sehr vielversprechend.^70^ Heute werden fast ausschließlich bipolare Elektroden eingesetzt. Sie sind deutlich weniger störanfällig und minimieren das Risiko für extrakardiale Mitstimulation, Myopotential-Oversensing und elektromagnetische Interferenz (EMI).

#### Venöser Zugangsweg

Mit der endokardialen Stimulation über einen venösen Zugang eröffnete sich das Feld der Herzschrittmacherimplantation auch für Nichtchirurgen. Bis Ende der 1970er-Jahre führten Chirurgen die meisten Implantationen durch. Danach setzte ein allmählicher Wandel ein: In den 1990er-Jahren waren weniger als die Hälfte der implantierenden Ärzte in den USA noch Chirurgen^71^. Ein wichtiger Impuls hinter dieser Veränderung war die Entwicklung eines Einführbestecks, das den Zugang zu einer Zentralvene ermöglichte und durch das eine Schrittmacherelektrode eingeführt werden konnte. In zwei Erstbeschreibungen von *Batson*^72^1973 und von *Jachuck*^73^1974 wurden Seldinger-Schleusen benutzt, die nach Einführung der Sonde längs aufgeschlitzt werden mussten, um wieder entfernt zu werden. In **Österreich** wurde das Verfahren von *Heinz Sterz*^74^aus Klagenfurt 1975 als Alternative zur Cephalica-Präparation beschrieben. 1979 stellte *Philip Littleford*^75^aus Orlando, USA, eine Modifikation der Seldinger-Schleuse vor, die längs aufgerissen („peeling“) werden konnte und berichtete über 164 Patienten, die über diesen Zugangsweg ihren Herzschrittmacher erhalten hatten.

In der Folgezeit gewann dieser Zugangsweg immer mehr an Popularität. In **Deutschland** berichtete *Dieter Kronski*^76^2001 über 2349 Patienten, die in **München**-Schwabing seit 1994 eine Subclavia-Punktion erhalten hatten und nur noch 36 Patienten (1,5 %) eine Cephalica-Präparation. Als Komplikationen zeigten sich gelegentliche, aber klinisch nicht relevante arterielle Fehlpunktionen. Nervenläsionen oder Pneumothoraces mit der Notwendigkeit für eine Drainage wurden nicht beobachtet. Zu einem anderen Ergebnis kommt *Andreas Markewitz* im **Jahresbericht des Deutschen Herzschrittmacher-Registers**^77^. Bei einem Anteil der Subclavia-Punktion von zuletzt 60 % war die perioperative Komplikationsrate mit 2,9 % signifikant gegenüber der Cephalica-Präparation mit 2,3 % erhöht und ein Pneumothorax trat mehr als 3‑mal häufiger (0,69 % vs. 0,2 %) auf. Die Daten aus NRW^78^von 2010 bis 2014 bei 139.176 Patienten zeigten eine 1,5-fach höhere Komplikationsrate und eine 5,5-fach höhere Pneumothoraxrate bei der Subclavia-Punktion.

In einem Vergleich zwischen Silikon- und Polyurethan-isolierten Elektroden (beide zu 95 % bipolar) fand *Dante Antonelli* aus Ferrara, Italien, 1998 im Verlauf von 57 ± 30 Monaten Ausfälle in 11,3 % nur bei den Polyurethanelektroden. Dabei erwies sich der venöse Zugangsweg als einziger unabhängiger Prädiktor für die Vorhersage des Elektrodenversagens: 16,8 % bei Verwendung der Vena subclavia und 4,9 % bei Verwendung der Vena cephalica (*p* = 0,03). Als Erklärung wurde angegeben, dass die mechanische Elektrodenkompression zwischen der ersten Rippe und dem Schlüsselbein zu destruktiven Druckkräften führen kann, die den Abbau der Isolation durch Oxidation und Kettenspaltung beschleunigt und schließlich zu mechanischem Versagen führt^79^. In einer retrospektiven Kohorten Studie mit 681 Elektroden und einer Nachbeobachtungszeit von 73 ± 33 Monaten war die Subclavia-Punktion mit 5,6 % Sondenkomplikationen dem Axillariszugang mit 1,2 % signifikant unterlegen. Die Komplikationsrate bei der Cephalica-Präparation betrug 2,3 %^80^. In zwei Metaanalysen fand sich eine 75 %ige Risikoreduktion von Sondenkomplikationen bei der Cephalica-Präparation im Vergleich zur Subclavia-Punktion^81^, bzw. eine Verdoppelung des Risikos bei Subclavia-Punktion^82^.

#### Elektrodenspitze

Die Stimulationsreizschwellen, die für die frühen transvenösen Elektroden charakteristisch waren, erforderten große Sicherheitsspannen mit Impulsdauern von 1,8 ms und Stimulationsamplituden von mehr als 5,5 V. Dies führte zu einer hohen Batteriestromentnahme, was große und schwere Pulsgeneratoren, einige > 200 g, erforderlich machte. Dies war der Anstoß, die Stimulationsenergie zu reduzieren. So begann eine Erforschung der Wechselwirkung zwischen Technologie und Physiologie mit dem Ziel, eine chemisch inerte, biokompatible, korrosionsfreie Elektrode zu entwickeln, die eine hohe Stromdichte, minimale Polarisationsverluste, niedrige stabile akute und chronische Spannungs- und Stromschwellenwerte und nahezu gleiche Wahrnehmungs- und Stimulationsimpedanzen aufwies.

In **Deutschland** waren vor allem *Werner Irnich* von der TU **Aachen** und der Universität **Gießen** sowie *Max Schaldach* von der Universität **Erlangen-Nürnberg** und Gründer von Biotronik, in den USA *Seymour Furman* vom Montefiore Hospital in New York sowie *Kenneth B. Stokes*^83^, Director of Brady Leads Research, Medtronic und *Gerald Timmis* vom Royal Oak Hospital, Michigan, für grundlegende Erkenntnisse und wegweisende Entwicklungen auf dem Gebiet der Elektrodenentwicklung verantwortlich.

Die frühen Elektrodenspitzen wiesen eine Kugel‑, Halbkugel- oder Zylinderform und eine glatte Metalloberfläche von 90 mm^2^ (Medtronic 5816) und später 29 mm^2^ (Biotronik IE-60-K) auf. Diese großen Oberflächen bewirkten einen hohen Shuntstrom, niedrige Stimulationsimpedanz mit hohem Stromverbrauch. *Furman* beschrieb 1969 bei temporären Stimulationskathetern den Zusammenhang zwischen Oberflächengröße und Reizschwelle und wies 1970 bei implantierbaren Elektroden eine lineare Beziehung zwischen beiden Größen nach^84^. Eine **Verringerung der Elektrodenoberfläche** um etwa 80 % bei den Cordis-Elektroden von 50 mm^2^ auf 8 mm^2^ führte zu einer etwa 30-fachen Verringerung der Schwellenenergie, führte jedoch zu Verlusten des Wahrnehmungssignals, auch bei der Medtronic Elektrode 6901^85^. *Irnich* schätzte den Radius einer optimal bemessenen halbkugelförmigen Elektrode, unterhalb dessen eine weitere Verringerung der Elektrodengröße keine zusätzliche Verringerung der Reizschwelle bewirkt, auf 0,6 mm, was ungefähr der Dicke der Bindegewebsreaktion entspricht, die sich um die Elektrodenspitze herum bildet^86^. 1975 leitete *Furman* aus seinen Untersuchungen ab, dass bei Verwendung von Elektroden mit kleiner Oberfläche die Impulsdauer von 1 ms auf 0,2–0,3 ms verkürzt werden könnte, um die Laufzeit der Herzschrittmacher von 2 auf 4 Jahren zu verdoppeln^87^.

Der reduzierten Reizschwelle durch Miniaturisierung der Elektrodenspitze stand der **Polarisationseffekt** gegenüber, der durch Stromabgabe ins angrenzende Myokard entsteht. Er ist abhängig von der Impulsbreite und umgekehrt proportional zur Elektrodenoberfläche. Bereits Anfang der 70er-Jahre wurde daran gearbeitet, die Polarisationsspannung zu verringern. 1971 führte *Schaldach* in **Deutschland** die dielektrische Schrittmacherelektrode (DPE) als Modifikation des Konzepts einer Elektrode mit differentieller Stromdichte (DCD) ein^88^. Die DPE verwendete eine Tantaloxidbeschichtung, um Polarisationsverluste zu reduzieren. Die Elektrodenoberfläche war jedoch zu groß, um die Schwellen dramatisch zu verbessern. Trotzdem erwies sich die entwickelte Technologie als nützlich, um Polarisationsverluste zu reduzieren.

Mit der Modifikation der Elektrodenoberfläche sollte auch die Biokompatibilität verbessert werden, die die fibrotische Reaktion zwischen Herz und Elektrode bestimmt und zum chronischen Reizschwellenanstieg führt. Zur Verbesserung der Wahrnehmungseigenschaften wurden eine Vielzahl **poröser Elektrodenspitzen** entwickelt, welche zu einer Reduktion der Wahrnehmungsimpedanz führten. 1979 konnte *MacGregor* beim Hund nachweisen, dass es tatsächlich zu Gewebeeinwuchs in den porösen Zwischenräumen der Elektrode kam^89^, und *Guarda* zeigte bei Schafen, dass der Entzündungsprozess an der Gewebe-Elektroden-Grenzfläche im Vergleich zu glatten Platin-Iridium-Elektroden begrenzter war^90^. Um die Polarisation noch weiter zu reduzieren, wurden Kohlenstoffelektroden, bestehend aus einem Graphitkern und einer aktivierten Kohlenstoffoberfläche entwickelt. Sie erzeugten ungefähr den gleichen Grad an Fibrose an der Elektroden-Gewebe-Grenzfläche wie gesinterte Platin-Iridium-Elektroden und weniger als herkömmliche Metallelektroden.

*Jacques Mugica* aus St. Cloud, Frankreich, berichtete 1986 über 910 **Carbon-Elektroden**, die in seiner Klinik in St. Cloud, Frankreich, seit 1980 implantiert und mit polierten Platinelektroden verglichen wurden. Die beiden Elektroden ELA PMCF 860 und Sorin S 100 mit pyrolytischen Kohlenstoffspitzen zeigten nach 12 Monaten signifikant niedrigere chronische Reizschwellen als die Platinspitzen mit gleicher Form und Oberfläche. Die Komplikationsrate der Elektroden lag unter 2 % und die meisten Patienten konnten sicher mit einer Amplitude von 2,5 V bei einer Impulsdauer von 0,5 ms stimuliert werden^91^.

Eine kohlenstoffbeschichtete poröse Titanelektrode mit signifikant niedrigeren Reizschwellenwerten wurde 1981 von Intermedics eingeführt. 1983 testete Medtronic im Tierversuch die Target-Tip-Elektrode, die aus einer gerillten Platinoberfläche bestand und mit Platinpartikeln von weniger als 1 µ beschichtet war, die stark reduzierte Reizschwellen aufwies.

Weitere Fortschritte wurden erzielt, als *Kenneth Stokes* und *Gerald Timmis* aus Michigan, USA, 1983 die **steroidfreisetzende Kathode** einführten. Hierzu wurde der Silikonkern dieser Elektrode mit weniger als 1 mg Dexamethason-Natriumphosphat imprägniert. In Tierversuchen zeigte sich, dass dieses über einen Zeitraum von etwa 10 Tagen durch die poröse Spitze der Elektrode an der Grenzfläche zwischen Elektrode und Gewebe diffundiert. Bestimmungen des Restvorrats an Dexamethason in explantierten Sonden zeigten, dass auch Jahre nach der Implantation noch Steroid freigesetzt werden konnte. Der Verlauf der akuten Reizschwellenkurven war für die steroidhaltige poröse Elektrode (Medtronic 4003) im Vergleich zu konventionellen Elektroden (Medtronic 6971) signifikant niedriger^92^. *Harry Mond* aus Melbourne, Australien und *Kenneth Stokes* berichteten 1996 über den 10-Jahres-Verlauf einer doppelblinden Humanstudie mit anfangs 20 implantierten unipolaren ventrikulären Elektroden. Während des gesamten Studienzeitraums blieben die mittleren Stimulationsschwellen für die steroidfreisetzenden Elektroden mit einer engen Standardabweichung nahezu konstant, während die Elektroden ohne Steroid einen unvorhersehbaren Anstieg und eine breite Standardabweichung zeigten. Alle Patienten mit steroidfreisetzenden Elektroden wurden mit 1,5 V stimuliert^93^. Über ähnliche Befunde berichtete *Bernhard Schwaab* aus **Homburg/Saar** 1997 für die aktiv fixierbaren Elektroden, wobei diese sich in ihrem mikroporösem Design nicht von den steroidfreien Elektroden unterschieden^94^.

In den 90er-Jahren wurde von vielen Herstellern ein **Hochimpedanz-Konzept** verfolgt, um den Stromverbrauch weiter zu reduzieren. Fast alle Daten über Elektroden mit sehr kleinen (1,2–1,5 mm^2^) Oberflächen bezogen sich auf platinierte, Steroid freisetzende Elektroden, mit Ausnahme einer, in **Deutschland** hergestellten Elektrode mit einem „fraktal“ nichtsteroiden Design (Biotronik SD-V137). Bei der von *Schaldach* 1991 beschriebenen „Fraktal“-Elektrode wurde die Oberfläche durch eine sog. Sputter-Technik erzeugt, die die grundlegende Struktur wiederholt und zu einer Vergrößerung der aktiven Oberfläche um einen Faktor von 2^n^ führt, wobei *n* mit 10–12 angegeben wird^95^. *Gerd Fröhlig* aus **Homburg/Saar** konnte 1998 zeigen, dass fraktal beschichtete Elektroden mit einer Elektrodenoberfläche von 1,3 mm^2^ im Vergleich zu 10 mm^2^ eine mehr als doppelt so hohe Impedanz aufwiesen (1344 ± 376 vs. 538 ± 79 Ω) bei signifikant niedrigerer Ladungsreizschwelle (0,15 ± 0,15 vs. 0,66 ± 0,20 µC)^96^. Die 3‑Jahres Ergebnisse von *Dejan Danilovic* aus Bergen, Norwegen, mit 66 steroideluierenden Hochohmelektroden bei 1‑ und 2‑Kammer-Schrittmachern ergaben im Vergleich mit herkömmlichen Elektroden zwar eine 39 % bzw. 62 %ige Verlängerung der Batterielebensdauer, aber nur im Vergleich zu einer Nominaleinstellung mit 3,5 V und 0,4 ms Impulsdauer. Im Vergleich zu einer Programmierung mit 1,6 V und 0,6 ms betrug die Einsparung nur noch 11 %^97^. Wegen des vergleichsweise hohen internen Stromverbrauchs des Herzschrittmachers setzte sich die Energieeinsparung nicht in entscheidende Laufzeitvorteile um und betrug bei optimierter Programmierung weniger als 10 %. Dagegen bedurften Hochimpedanz-Elektroden einer sorgfältigen Platzierung mit perfektem Wandkontakt, um *Mikrodislokationen* mit nachfolgender Reizschwellenerhöhung zu vermeiden^98^.

Mit der Einführung der Demand-Schrittmacher (siehe Kap. „Bedarfsschrittmacher“) mussten die Schrittmacherelektroden eine **Wahrnehmung des QRS-Signals** im Ventrikel bewerkstelligen. Die effektive Kammerstimulation mit einer geringen Reizschwelle ist unter anderen Faktoren wie Elektrodenmaterial, Elektrodengeometrie, Polarisationseffekte am Elektrodenübergang zum Myo-Endokard (Helmholtz-Schicht) vor allem von der Oberflächengröße der Stimulationselektrode abhängig. Die Problematik dieser Doppelfunktion, Stimulation und Wahrnehmung des herzeigenen Signals liegt darin, dass sich beide Funktionen bezogen auf die Elektrodengröße diametral verhalten: Eine kleine Elektrodenoberfläche ist für eine niedrige Stimulationsreizschwelle von Vorteil, für die Wahrnehmungsfunktion (Sensing) ist eine größere Elektrodenoberfläche vorteilhaft, da damit die Polarisation am Übergang vom Myokard zur Elektrode reduziert wird. Dadurch wird der Spannungsabfall des herzeigenen Signals an der Grenzschicht verringert, was zu höheren und stabileren R‑Wellen, bzw. zur besseren Wahrnehmung führt. Die Lösung dieses Problems wurde mit den beschichteten und bearbeiteten Elektroden durch Aufbringen von gerillten, porösen und fraktalen Strukturen an den Oberflächen gefunden. *Gerd Fröhlich* aus **Homburg/Saar** zeigte 1998, dass bei den so bearbeiteten Elektrodenoberflächen strukturelle Bereiche mit sehr hoher Ladungsdichte entstehen, welche zu einer geringeren abgegebenen Ladung (µC) an der Stimulationsreizschwelle führen. Gleichzeitig erlauben sie das Einwachsen des Endomyokardgewebes in die Vertiefungen, was de facto zu einer größeren Oberfläche und somit zur einer verbesserten Wahrnehmungsfunktion führt.

### Sondenlose Herzschrittmacher

Schon früh gab es Versuche, die mit den Schrittmacherleitungen verbundenen Probleme dadurch zu beseitigen, indem man einen Stimulator entwickelte, der direkt an der Stimulationsstelle implantiert werden konnte. *John Schuder* aus Columbia, USA, untersuchte 1962 die Möglichkeit, die für die Herzstimulation erforderliche Energie durch direkte induktive Kopplung zwischen einer externen Spule und einem extrem kleinen Schrittmacherempfänger, der direkt auf eine Herzkammer genäht wurde, zu transportieren. Die experimentellen Ergebnisse an 17 Hunden bestätigten, dass das Herz zuverlässig durch Induktion zu stimulieren war^99^. Auch *Furman* untersuchte 1966 das Prinzip der direkten Stimulation des Herzens durch kleine Radiofrequenzempfänger^100^.

**Abb. 3:**
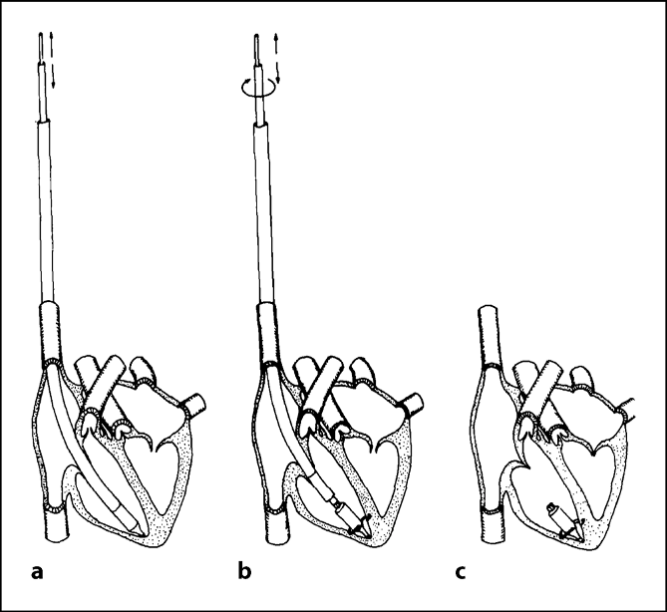
Platzierung eines intrakardialen Schrittmachers über einen Führungskatheter. **a** Insertion, **b** Attachment, **c** Implanted. (Spickler JW, Rasor NS, Kedi P, Misra SN, Robins KE, LeBoeuf C. Totally self-contained intracardiac pacemaker. J. Electrocardiology 1970; 3:325–331) © Elsevier, mit freundl. Genehmigung

*William Spickler* vom Cox Heart Institute in Ohio, USA, gelang 1970 beim Hund die erste Operation eines vollständig im Herzen implantierten Schrittmachers mit integrierter Elektrode und Befestigungsmechanismus. Mit einem Durchmesser von 8 mm und einer Länge von 18 mm war er so klein, dass er über die V. jugularis mit einem Führungskatheter in der Spitze des rechten Ventrikels platziert werden konnte (Abb. 3). Das erste funktionierende Testmodell verwendete eine Quecksilberbatterie und asynchrone Stimulation und funktionierte über 2 Monate, bevor die Batterie erschöpft war. Um die grundsätzliche Machbarkeit eines intrakardialen Schrittmachers mit langer Lebensdauer zu demonstrieren, wurde ein experimentelles Modell mit einer „Betacel“-Nuklearbatterie gebaut und bei einem Hund implantiert. Sie stimulierte das Herz mit einer Frequenz von 100 Schlägen pro Minute und sollte eine voraussichtliche Lebensdauer von 5 Jahren haben^101^. Die nuklearbetriebenen Geräte konnten aber aufgrund von Sicherheitsbedenken und kurzer Batterielebensdauer nicht praktisch eingesetzt werden (siehe auch Kapitel „Batterieentwicklung, Nukleargenerator“).

1991 berichtete *Panos Vardas*, Heraklion, Griechenland, über einen Miniaturschrittmacher, implantiert in 8 Hunden. Er wurde mit drei 1,5-V-Batterien betrieben und hatte keine Wahrnehmungsmöglichkeit. Daher wurde dieser Herzschrittmacher als nicht geeignet für die klinische Anwendung erachtet, obwohl die Stimulation bei den Hunden erfolgreich war^102^.

1999 testete *H. Goto* vom Research Institute of Electronics, Shizuoka University, Hamamatsu, Japan ein automatisches Stromerzeugungssystem (AGS), das Bewegungsenergie in elektrische Energie für Quarzuhren (SEIKO) umwandelte, als Energiequelle für implantierbare Herzschrittmacher. Das AGS wurde an der rechten Ventrikelwand eines Mischlingshundes platziert und erzeugte 13 µJ pro Herzschlag und damit genügend Energie für die Verwendung in einem vollständig implantierbaren Herzschrittmacher^103^.

Im Jahr 2006 berichtete *Debra Echt* vom EBR Systems Inc.^104^, Sunnyvale, USA, über einen Ultraschallsender, der Energie von der Brustwand zu einer Empfängerelektrode in Kontakt mit dem Myokard leitet, wo die Ultraschallenergie in elektrische Energie umgewandelt und für die Stimulation genutzt wird^105^ (s. auch „Zukünftige Entwicklungen“, kardiale Resynchronisationstherapie).

In **Deutschland** brachte *Heinrich Wieneke* vom Universitätsklinikum in **Essen** 2009 erneut die Induktion ins Spiel. Dabei wurden eine subkutan direkt über dem Herzen implantierte Sendeeinheit und eine in der Spitze des rechten Ventrikels implantierte endokardiale Empfängereinheit verwendet. Die Sendeeinheit erzeugte ein magnetisches Wechselfeld von ca. 0,5 mT, das von der Empfangseinheit in einen Spannungsimpuls von 0,6–1,0 V bei 0,4 ms Impulsdauer umgewandelt wurde. Durch Verschraubung konnte eine sichere Fixierung der Empfängereinheit in der Spitze des rechten Ventrikels und eine zuverlässige Stimulation des Herzens erreicht werden^106^.

**Abb. 4:**
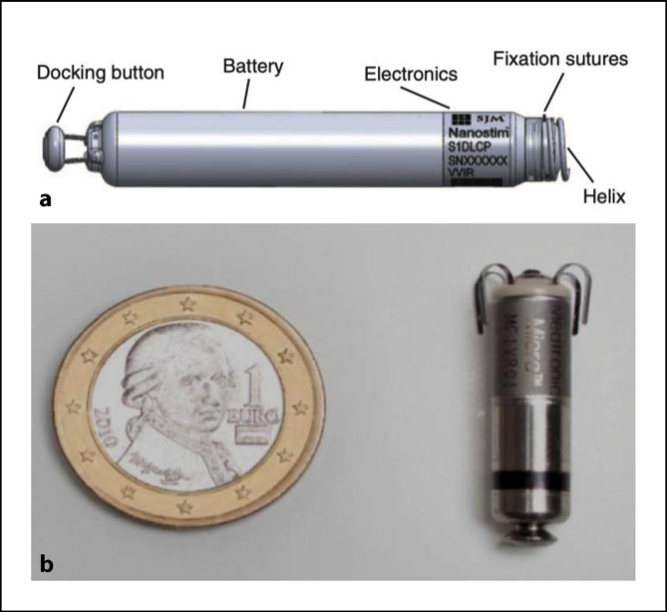
**a** Nanostim^TM^, St. Jude Medical, Sylmar, CA, USA, Leadless Pacemaker mit Steroid freisetzender Helix zur Fixierung im Myokard. Mit freundl. Genehmigung von Abbott © 2024. All rights reserved. **b**  Micra^TM^, Medtronic, Minneapolis, MN, USA, Transcatheter Pacing System mit einem Fixationsmechanismus aus 4 gebogenen Nitinolärmchen. (Sperzel J, Burri H, Gras D, Tjong FVY, Knops RE, Hindricks G, Steinwender C, Defaye P. State of the art of leadless pacing. Europace 2015; 17:1508–1513. Mit freundl. Genehmigung von Medtronic Inc.)

Ein vollständig im Herzen zu implantierendes System (**Nanostim**^**TM**^ Leadless Pacemaker System, St. Jude Medical, Sylmar, CA, USA) kam im Dezember 2012 erstmals im Rahmen der **LEADLESS-Studie** zur Anwendung. Das System mit einer Länge von 42 mm und einem maximalen Durchmesser von 6 mm wurde mit einer steuerbaren 18-F-Schleuse über die V. femoralis in den rechten Ventrikel eingeführt und durch eine distale, steroidfreisetzende Helix am Endokard fixiert (Abb. 4). Für die frequenzvariable Stimulation (VVIR) wurde ein temperaturbasierter Sensor verwendet. *Vivek Reddy* von der Mount Sinai School of Medicine, New York, USA, berichtete 2015 über eine erfolgreiche Implantationsrate von 97 % (*n* = 32) mit einer komplikationsfreien Gesamtrate von 94 % (*n* = 31)^107^.

Im Tierversuch wies in **Deutschland** *Johannes Sperzel* von der Kerckhoff Klinik in **Bad Nauheim** 2013 nach, dass bei allen 10 Schafen die implantierten Geräte nach 5 Monaten ohne Schwierigkeiten wieder explantiert werden konnten^108^.

2015 wurden von *Reddy* die Ergebnisse der großen Multicenterstudie (LEADLESS II) mit 526 Patienten veröffentlicht. Der elektrodenlose Herzschrittmacher wurde bei 95,8 % der Patienten erfolgreich implantiert. Nach 12 Monaten stieg die R‑Zacken-Amplitude von 7,8 mV auf 9,2 mV (*p* < 0,01), die Impedanz sank von 700 Ω auf 456 Ω (*p* < 0,01), und die Reizschwelle bei 0,4 ms sank von 0,82 V auf 0,58 V. Der primäre Sicherheitsendpunkt wurde von 93,3 % der Patienten erreicht. Dabei traten bei 6,7 % gerätebedingte schwerwiegende unerwünschte Ereignisse auf (Dislokation, Herzperforation, Erhöhung der Stimulationsreizschwelle)^109^. Die Batterielebensdauer für das Leadless-Pacing-System wurde auf 15,0 Jahre^110^ geschätzt.

2018 berichtete *Sperzel* aus **Bad Nauheim** über die Ergebnisse der LEADLESS Observational Post-Market-Studie, die die Sicherheit von Nanostim in einer realen Umgebung bewerten sollte. Sie wurde nach 131 Implantationen aufgrund von 2 Herzperforationen, die zum Tod der Patienten führten, abgebrochen. Nach Protokolländerungen und erneuter Implanteursschulung wurden bei 94,6 % (285 von 300 Patienten) nach 6 Monaten Nachbeobachtung keine schwerwiegenden unerwünschten Ereignisse mehr beobachtet. Am häufigsten waren 4 Herzperforationen (1,3 %) und 4 vaskuläre Komplikationen (1,3 %). Insgesamt ereigneten sich 11 Herzperforation (2,3 %), die nach der Studienpause durch eine Änderung des primären Implantationsortes von der Spitze zum Septum von 4,6 % auf 1,5 % abnahmen. Im Oktober 2016 stoppte der Sponsor alle weltweiten Implantationen nach Berichten über Batterieausfälle, die zu einem Verlust der Stimulationsleistung und der Kommunikation mit dem LP führten^111^.

Am 04. April 2022 gab die U.S. Food and Drug Administration (FDA) bekannt, dass dem modifizierten System Aveir™ nach Abschluss der LEADLESS II – Phase 2 Studie mit 200 Patienten die Zulassung in den USA erteilt wurde^112^. Die Änderungen umfassen eine um 12 % längere Batteriedauer, die jetzt mit 10,4 Jahren angegeben wurde, eine geänderte Form (10 % kürzer, und 1,5 F breiter), die jetzt einen 25-F-Einführungskatheter erforderlich machte, einen geänderten Andockkopf, um die Entfernbarkeit zu erleichtern und ein geändertes Applikationssystem. Die erfolgreiche Implantationsrate lag bei 98 %, die häufigsten Komplikationen waren 3 Fälle von Herzbeuteltamponade (1,5 %, alle während apikaler Positionierung, 2 mit Sternotomie) und 3 Fälle von vorzeitiger Freisetzung des Systems (1,5 %)^113^.

Das zweite System, Micra^TM^-Transkatheter-Stimulationssystem (Medtronic, Minneapolis, MN, USA), wurde ein Jahr nach dem Nanostim-Leadless-Pacemaker im Dezember 2013 im Rahmen der **Micra Transcatheter Pacing Study** erstmalig implantiert. Zuvor war von *Eggen*, Medtronic, ein neuer Fixationsmechanismus aus 4 gebogenen Nitinolärmchen an 89 Tieren und an 13 frisch explantierten menschlichen Herzen auf Stabilität und Entfernbarkeit getestet^114^und von *Bonner* zum Patent^115^ angemeldet worden. Das Gerät mit einer Länge von 25,9 mm und einem Außendurchmesser von 6,7 mm war am distalen Ende eines steuerbaren Katheterzuführungssystem befestigt und wurde über eine 27-F-Schleuse durch die Femoralvene platziert (Abb. 4). Bei 60 Patienten betrug die mittlere Reizschwelle 0,51 V bei einer Impulsdauer von 0,24 ms. Eine frühe Beurteilung nach 2–3 Monaten durch *Philippe Ritter*, Universität Bordeaux, Frankreich, zeigte, dass der Transkatheterschrittmacher sicher und effektiv angewendet werden konnte^116^. 2016 stellte dann *Dwight Reynolds* von der Universität von Oklahoma, USA, die Gesamtergebnisse der Studie vor. Das Gerät wurde bei 719 von 725 Patienten (99,2 %) erfolgreich implantiert. Im Verlauf von 6 Monaten stieg die R‑Zacken-Amplitude von 11,2 mV auf 15,3 mV, die Stimulationsimpedanz sank von 724 Ω auf 627 Ω und die Reizschwelle bei einer Impulsdauer von 0,24 ms sank von 0,63 V auf 0,54 V. Es kam zu 28 (4 %) schwerwiegenden Komplikationen: 1 Todesfall (0,1 %), 1 Funktionsverlust (0,1 %), 28 Hospitalisationen (4,9 %) und 2 Systemrevisionen (0,4 %). Herzperforationen und Perikardergüsse traten bei 11 Patienten (1,6 %) auf 117. Die Batteriefunktionsdauer für das Transkathetersystem wurde auf 12,5 Jahre geschätzt^118^,^119^.

2018 berichtete *Mikhael F. El-Chami* aus Atlanta, USA, über die Ergebnisse des Micra Post Approval Registers (PAR). Das Gerät wurde erfolgreich bei 1801 von 1817 Patienten (99,1 %) implantiert. Nach 12 Monate betrug die Rate an schwerwiegenden Komplikationen 2,7 %. Die Hauptkomplikationsrate war niedriger als in der Zulassungsstudie, was auf die niedrigere Rate an Perikardergüssen (0,44 %) zurückzuführen war^120^.

Die Frequenzreaktion (VVIR) beim Micra^TM^-Schrittmacher wurde mit einem 3‑Achsen-Beschleunigungsmesser erreicht (s. auch Kapitel „Sensorgesteuerte frequenzvariable Stimulation“). Dieser Sensor wurde auch eingesetzt, um die atriale Kontraktion zu detektieren und dadurch eine vorhofsynchrone Ventrikelstimulation (VDD) zu ermöglichen. *Clemens Steinwender* aus **Linz**, Österreich, bewertete in der MARVEL-2-Studie die Leistung dieses Algorithmus bei 40 Patienten mit Sinusrhythmus und komplettem AV-Block; hier konnte eine AV-Synchronität von 94 % in Ruhe erreicht werden^121^. *Felix Neugebauer* aus **Bern** fand bei 20 ambulanten Patienten mit einem sondenlosen VDD-Schrittmacher (Micra-AV, Medtronic) bei Sinusfrequenzen von 50–80 min^−1^ eine AV-Synchronität in 91 %, aber trotz mehrfacher Optimierung der Programmierung nur 33 %, wenn sie über 80 min^−1^ lagen^122^.

#### Zukünftige Entwicklungen

*Pierce J. Vatterott* aus St. Paul, USA veröffentlichte 2020 die Ergebnisse der Micra^TM^-Implantation im Vorhof bei 12 Schafen mit gutem Ergebnis^123^. Auch Abbott arbeitet an einem sondenlosen Vorhofschrittmacher, der zusammen mit einem Gerät im Ventrikel eine DDD-Funktion ermöglicht. In einer vorklinischen Tierstudie wurde die Machbarkeit der Kombination eines sondenlosen antitachykarden Schrittmachers mit einem S-ICD (Boston Scientific) getestet^124^. Nach der Aufnahme des ersten Patienten wird derzeit die klinische Studie MODULAR ATP (NCT04798768) durchgeführt, um die Leistungsfähigkeit eines solchen Systems zu testen.

Für den Bereich der kardialen Resynchronisationstherapie (CRT) bei Nonrespondern oder fehlendem Gefäßzugang wurde ein sondenloser Stimulator für den linken Ventrikel entwickelt. Dieses System besteht aus einem subkutanen Impulsgenerator, der über Schallenergie (Ultraschall) eine im LV-Endokard fixierte Stimulationselektrode anspricht. Die Energie wird in einen Stimulationsimpuls umwandelt und getriggert an ein DDD-System (Schrittmacher oder Defibrillator) im linken Ventrikel abgegeben. Die WiSE-CRT-Studie wurde gestoppt, nachdem bei 3 von 17 Patienten (18 %) eine Perikardtamponade auftrat, bei einem Patienten mit tödlichem Ausgang. Nach einer Modifikation des Applikationskatheters veröffentlichte *Reddy* 2017 die Ergebnisse der SELECT-LV-Studie mit 35 Patienten. Diese war zwar frei von Perikardtamponaden, es entwickelten sich aber akut und im Verlauf von 6 Monaten vermehrt schwerwiegende unerwünschte Ereignisse (39,5 %), darunter 3 Generatorinfektionen und 3 Defekte der Transmittereinheit^125^. (Näheres hierzu siehe den Beitrag zur „Kardialen Resynchronisationstherapie“.)

## Batterieentwicklung

### Quecksilber-Zink-Zellen, wiederaufladbare Nickel-Cadmium-Zellen, elektrochemische Verfahren, piezoelektrische Effekte

In den 1960er-Jahren bezogen nahezu alle Herzschrittmacher ihre Energie aus **Quecksilber-Zink-Zellen**, die ursprünglich während des Zweiten Weltkriegs für militärische Anwendungen entwickelt worden waren. Eine Firma belieferte alle Herzschrittmacherhersteller. Anfangs wurde eine Batteriefunktionsdauer von 5 Jahren prognostiziert, in der Praxis mussten die Herzschrittmacher aber nach Ablauf von 2 Jahren wegen Batterieerschöpfung ausgetauscht werden. Die Zellen wurden durch Selbstentladung fast ebenso stark erschöpft wie durch Stimulation, aber von größerer Besorgnis war, dass sie als Nebenprodukt Wasserstoffgas emittierten und daher der Impulsgenerator nicht hermetisch gegen das Eindringen von Körperflüssigkeiten abgedichtet werden konnte. „Wenn eine wirklich gute Stromquelle zur Verfügung stünde“, schrieb *Victor Parsonnet* im Jahr 1970, „könnte die große Mehrheit der Schrittmacherwechsel vermieden werden.“^126^

Die Unzufriedenheit mit den Quecksilber-Zink-Zellen führte zu einer Flut erfinderischer Aktivitäten mit völlig anderen Konzepten: wiederaufladbare Herzschrittmacherbatterien, eine biogalvanische Zelle, Bioenergiequellen wie das Antreiben eines Herzschrittmachers durch mechanische Wirkung der Aorta oder des Zwerchfells, Nukleargeneratoren und Lithiumbatterien.

Ohne die Erstimplantation durch *Senning* und *Elmqvist* zu erwähnen, schlug *Furman*^127^1965 einen **wiederaufladbaren Herzschrittmacher** mit **Nickel-Cadmium-Batterie** vor und postulierte, bei richtiger Behandlung hätten die Zellen eine nahezu unbegrenzte Lebensdauer. Das Wiederaufladen sollte über Hochfrequenzinduktion von Strom in die Schrittmachereinheit erfolgen und über einen Zeitraum von 8 Stunden dauern. *Silver*^128^nahm Bezug auf die Arbeiten von *Senning* und *Elmqvist* und berichtete 1965 über seine Ergebnisse mit einer wiederaufladbaren Nickel-Cadmium Batterie, die beim Hund mit chirurgisch induziertem AV-Block implantiert wurde. Hier dauerte die Wiederaufladezeit nur wenige Minuten am Tag.

In **Deutschland** berichtete *Schaldach* aus **Erlangen-Nürnberg** 1969 über ein **elektrochemisches Verfahren**. Die Elektroden der galvanischen Zelle befanden sich an den gegenüberliegenden Seiten eines zylindrischen Herzschrittmachers. Die Anode aus Zink diente gleichzeitig als indifferente Elektrode für die Stimulation, die Kathode bestand aus einer Mischung aus Silber und Silberchlorid. Um eine sofortige Oxidation des Zinks zu erreichen, sollte der Herzschrittmacher in gut durchblutetem Gewebe eingesetzt werden.

Beim Kontakt der galvanischen Zelle mit den Elektrolyten des Körpers begann der Herzschrittmacher spontan zu stimulieren. Vierzehn dieser Herzschrittmacher wurden in den Musculus pectoralis major implantiert, der Rest in subkutane Gewebetaschen. Bei subkutaner Implantation kam es durch eine Ansammlung von Exsudat zu einer schmerzhaften Gewebereaktion. Am 10. Tag war der Erguss im Bereich um den Herzschrittmacher abgeklungen. Bei intramuskulären Implantationen waren die Gewebereaktionen deutlich weniger ausgeprägt. Eine Einheit musste wegen einer Drucknekrose in der subkutanen Tasche entfernt werden.

In einer klinischen Versuchsdauer von 10 Monaten traten sowohl im Tierversuch als auch bei den Patienten keine schwerwiegenden Nebenwirkungen auf, und es konnte gezeigt werden, dass es durch galvanische Zellen möglich ist, Energie aus biologischem Gewebe zu gewinnen^129^. Die langfristige Verwendung dieses Geräts zeigte aber, dass die Anode mit einer viel größeren Rate als erwartet verbraucht wurde und nur begrenzte Energiemengen entnommen werden konnten, so dass sich kein Vorteil gegenüber den Standard-Quecksilber-Zink-Zellen ergab.

Es wurde versucht, die kinetische Energie natürlicher beweglicher Körperteile zu nutzen, um Elektrizität zu erzeugen. Eine Methode, die sich den **piezoelektrischen Effekt** zunutze machte, hatte sich als geeignet erwiesen, einen Herzschrittmacher zu betreiben. Sowohl die Expansion als auch die Kontraktion der Aorta und die Pulsation des Herzens selbst wurden auf diese Weise verwendet. Das Hauptproblem war die Einkapselung der Keramikkristalle, welche die Effizienz solcher Vorrichtungen stark einschränkte^130^.

### Nukleargenerator

Als weitere Alternative zur Quecksilber-Zink-Batterie wurde in den 1960er-Jahren der nuklear betriebene Generator entwickelt und Anfang der 1970er-Jahre zur klinischen Anwendung gebracht. Dabei wurde die Strahlungsenergie, die beim Zerfall radioaktiver Isotope entsteht, direkt oder indirekt in elektrische Energie umgewandelt.

^238^Plutonium zerfällt mit einer Halbwertzeit von 86,4 Jahren unter Aussendung von α‑Strahlen. Bei Verwendung dieses Isotops erfolgte die Umwandlung der kinetischen Energie der emittierten α‑Teilchen in elektrische Energie über die im angrenzenden Medium entstehende Wärme in thermoelektrischen Elementen (Thermocouple). Ein anderer Weg wurde bei Verwendung des Isotops ^147^Promethium beschritten, ein β‑Strahler mit einer Halbwertzeit von 2,6 Jahren. Durch die stark ionisierende Wirkung ließ sich die kinetische Energie der β‑Teilchen über einen Semi-Kontaktor direkt in elektrische Energie umwandeln.

Der gemeinsam mit der französischen Atomenergiekommission durch die Firmen Alcatel und Medtronic entwickelte Herzschrittmacher mit dem **thermoelektrischen Isotopenelement** ^**238**^**Plutonium** wurde erstmals im April 1970 von *Laurens* und *Piwnica* in Paris implantiert^131^. 1971 führten *Christoph Reidemeister* und *Wolfgang Bircks* in **Düsseldorf** die ersten 7 Implantationen eines Nuklearschrittmachers (Laurens-Alcatel Model Medtronic 9000) in **Deutschland** durch^132^. In den USA fand die erste Implantation eines Nuklearschrittmachers erst im April 1973 durch *Parsonnet* am Newark Beth Israel Medical Center statt^133^. Basierend auf einem weltweiten Register von *Laurens* berichtete *Reidemeister* im Juli 1974 über 886 Nuklearschrittmacher Typ 9000 der Firma Medtronic, die weltweit implantiert wurden, 225 in Deutschland, davon 36 in Düsseldorf und in **Essen** bei Patienten im Alter zwischen 23 und 56 Jahren. Bei den 20 weltweit verstorbenen Patienten wies nur ein Gerät einen elektronischen Defekt auf, die meisten Aggregate wurden erneut implantiert^134^. Ausgehend von den 3‑Jahres-Daten postulierte *Laurens* eine erwartete 10-Jahres-Funktionsrate von 90 % und eine 20-Jahres-Rate von 80 %.

Dies bestätigte *Christophe Chauvel* 1995 durch die Nachbeobachtung von 325 Patienten, die zwischen April 1970 und Juli 1982 im Lariboisière University Hospital von Paris einen Nuklearschrittmacher der Firma Medtronic (Typ 9000/9090) erhalten hatten. Die kumulative Funktionsrate des Geräts betrug 97 % nach 18,5 Jahren. Während einer mittleren Nachbeobachtungszeit von 12 Jahren wurden 122 Reoperationen bei 85 Patienten durchgeführt. Bei 68 % der 122 Reoperationen lagen Funktionsstörungen der epimyokardial implantierten Elektroden vor, bei 6 % Generatorversagen und bei 26 % sonstige Ursachen; 72 % der Patienten blieben während der Nachbeobachtungszeit ohne Eingriff und 61 Patienten (20 %) starben während dieser Zeit^135^. Die Verwendung dieses Radioisotopen-Impulsgenerators wurde 1982 aufgegeben, da der Hersteller beschlossen hatte, die Produktion einzustellen.

1990 berichtet *Parsonnet* über die Erfahrungen mit den ersten 132 Patienten, die im Newark Beth Israel Medical Center zwischen 1973 und 1987 insgesamt 155 Nuklearschrittmacher der Firmen Cordis, Medtronic, Coratomic und ARCO erhalten hatten und über einen Zeitraum von 15 Jahren nachbeobachtet werden konnten. Ein Ausfall der Stromquelle wurde nur in einem Fall beobachtet, 47 Nuklearschrittmacher wurden aus anderen Gründen entfernt, darunter Komponentenfehlfunktionen in 11 % und ein Moduswechsel von VOO auf VVI in 9 %. Hohe Stimulationsreizschwellen und Elektroden- oder Steckerprobleme der zu 95 % endovenös implantierten Elektroden traten in 10 % auf. Die kumulative Funktionsrate nach 15 Jahren betrug 99 % für die Stromquellen und 82 % für die gesamten Herzschrittmachersysteme. Trotz dieser positiven Erfahrungen wurden in den USA seit 1983 nur noch sehr wenige Nuklearschrittmacher implantiert. Eine der ernsthaften Bedenken des NRC^136^ und der FDA^137^ war die Möglichkeit, dass eine längere Strahlenexposition krebserregend sein könnte. Diese Besorgnis bestand trotz vieler theoretischer Modelle und physikalischer Messungen und Berechnungen, dass die Strahlendosis innerhalb akzeptierter Grenzen lag. Die Formel, die die Wirkung der Strahlung auf das Flächendosisprodukt der Körperoberfläche bezog, schien bei jungen Patienten mit einer implantierten Strahlungsquelle kein geeigneter Test zu sein. Alle Hersteller, außer einem, stellten in den USA die Produktion ein. Der Hersteller Biocontrol Technology erreichte noch 1988 die Zulassung für einen 2‑Kammer-Schrittmacher (DDD), doch es war fraglich, ob er die Produktion bei der kritischen Haltung der medizinischen Gemeinschaft zur Nuklearbatterie weiter fortsetzen würde.

**Abb. 5:**
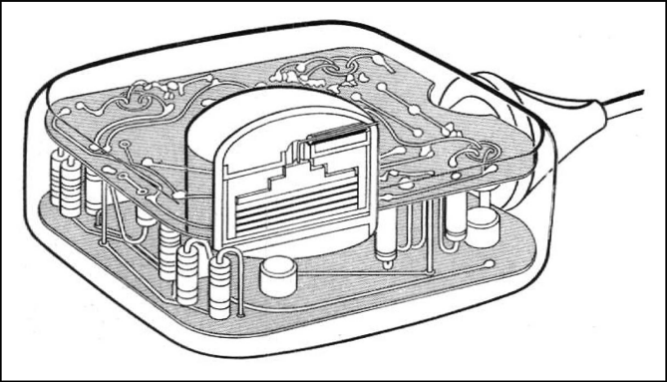
Querschnitt eines Biotronik-Schrittmachers mit „Betacel 400“ Zelle. (© Georg Thieme Verlag KG, mit freundl. Genehmigung. Rosenkranz KA, Schaldach M. Zur Anwendung kernenergiebetriebener Herzschrittmacher mit transvenös-endokardialer Elektrostimulation. DMW 1973; 98:2227–2233)

Die Ergebnisse des von den Donald W. Douglas-Laboratories in den USA in Zusammenarbeit mit dem Institut für Biomedizinische Technik der Universität **Erlangen-Nürnberg** entwickelten Herzschrittmachers mit dem Isotop ^**147**^**Promethium** und **betavoltaischem Konversionsprinzip**, berichtete *Karl-Adolf Rosenkranz* aus dem **Bergmannsheil Bochum** 1973. Im Gegensatz zu den anderen Atomschrittmachern im V00- und VVI-Modus bestand bei diesen Systemen die Möglichkeit zur Vorhofsteuerung (VAT) oder Vorhofstimulation (AAI). Die erste Implantation eines vorhofgesteuerten Schrittmachers (Betacel 400 Biotronik IKP 53) erfolgte am 08.02.1973 bei einem 16-jährigen Mädchen mit totalem AV-Block nach Verschluss eines Vorhofseptumdefektes. In der Publikation wird auch der Effekt einer Vorhofstimulation bei sinuaurikularem Block einer 53-jährigen Frau beschrieben. Insgesamt wurden bei 16 Patienten im durchschnittlichen Alter von 52,6 Jahren Biotronik-Schrittmacher mit Radionuklidbatterie implantiert^138^ (Abb. 5).

Bei 5 Patienten mittleren Alters wurden in der Klinik für Kardiologie der **Charité Berlin**, Pm^147^-Geräte eingesetzt. Sie konnten unterschiedliche Elektronik enthalten und wiesen verschiedene Stimulationsmodi auf. 2013 fiel bei der Vorbereitung einer Sonderausstellung „Visite im Depot“ des Berliner Medizinhistorischen Museums der Charité ein explantierter Pm^147^ Herzschrittmacher (Betacel Biotronik IKP-51) auf, der nicht vorschriftsmäßig entsorgt worden war^139^.

Die Möglichkeiten zur Ultra-Miniaturisierung der Pm^147^ Betacel-Batterien wurde bereits 1970 in einem experimentellen intrakardialen und sondenlosen Herzschrittmacher von *William Spickler*^140^ untersucht (siehe auch Kapitel „Sondenlose Herzschrittmacher“), den das Cox Heart Institute in Ohio entwickelt hatte und der durch transvenöse Einführung in das Hundeherz implantiert wurde. Der Herzschrittmacher hatte einen Durchmesser von 8,5 mm und die Stromquelle ein Volumen von 0,7 cm^3^.

Die Batterie des konventionellen Pm^147^-Herzschrittmachers wurde dagegen mehrfach abgeschirmt und eingekapselt, daher waren diese Implantate mit 174 g selbst für die damalige Zeit sehr schwer. Aufgrund der kurzlebigen Isotope bei den Betavoltaikbatterien (Pm^147^ Promethium mit einer Halbwertszeit von 2,6234 Jahren) mussten die Herzschrittmacher meist nach 5 Jahren (ca. doppelte Halbwertszeit) entfernt werden. Das Bundesamt für Strahlenschutz (BfS) sagte dazu 2011: „Wegen ihrer geringen Halbwertszeit ist ein Großteil der bis Ende der 1970er-Jahre eingesetzten radioaktiven Stoffe seitdem ‚zerfallen‘ und stellt heute kein Problem dar“. Dies gilt natürlich nur für kurzlebige Isotope wie Pm^147^ (Promethium).

Weltweit wurden mehr als 3000 Nuklearschrittmacher verschiedener Hersteller implantiert. Ein abschließender Bericht über diese gesamte Erfahrung wurde nie veröffentlicht.

In Westdeutschland erhielten zwischen 1971 und 1976 284 Patienten einen Herzschrittmacher mit Nuklearbatterie. Nur einer der in Deutschland eingebauten Herzschrittmacher ist 1976 zusammen mit seinem Träger in Kambodscha verschollen. Im deutschen Register standen 2009 noch zwei lebende Patienten – doch es gibt eine Dunkelziffer nicht registrierter Plutoniumbatterien. In den Staaten des ehemaligen Ostblocks wurden die Atomschrittmacher mindestens bis Ende der 1980er-Jahre implantiert. Es ist daher davon auszugehen, dass Patienten mit solchen Herzschrittmachern – wahrscheinlich als Spätaussiedler – nach Deutschland gezogen sind. Eine 64-Jährige Patientin stellte sich 2009 in Halle in einer kardiologischen Praxis mit ihrem 1986 in der Sowjetunion implantierten Nuklearschrittmacher (Tenzor, Modell REKS 2203) vor^141^. Auch in Leipzig wurde eine Patientin mit ihrem über 50 Jahre alten Herzschrittmacher vorstellig. Sie sagte, er sei programmierbar, aber den Kollegen fehlte natürlich das Programmiergerät^142^. Im Register des Bundesamtes für Strahlenschutz tauchen diese Patienten aber nicht auf. Es gibt keine Meldepflicht, keine gesetzliche Zuständigkeit für die Erfassung dieser Plutoniumbatterien. Mindestens ein Patient ist in Deutschland schon beerdigt worden, ohne dass der Atomschrittmacher entfernt wurde. Das BfS schätzt aber die Gefahr bei Feuerbestattung, bei Müllverbrennungsanlagen durch Einschmelzen mit Metallschrott und durch Deponierung und Erdbestattung als eher gering [!] ein^143^. Auf der aktuellen Homepage des BfS finden sich hierzu keine Informationen mehr.

**Abb. 6:**
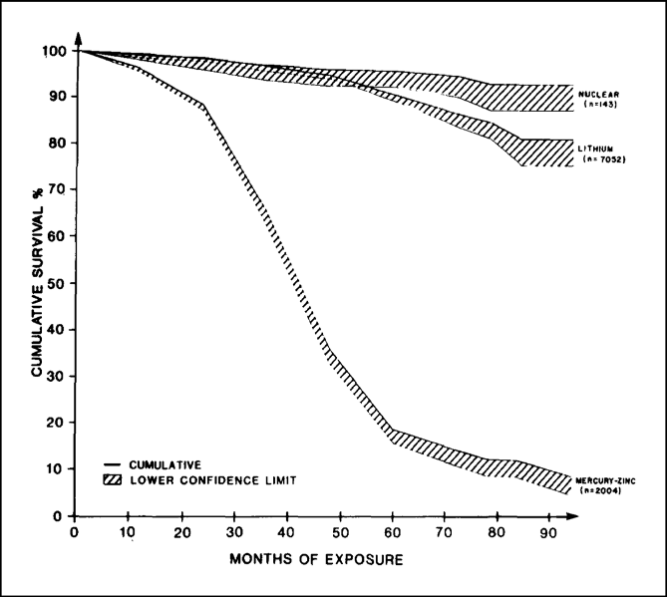
Kumulative Überlebensraten von Impulsgeneratoren in Abhängigkeit von der Stromquelle. Die bemerkenswerte Langlebigkeit von Lithiumbatterien im Vergleich zu Quecksilber-Zink-Batterien ist offensichtlich. (© Elsevier, mit freundl. Genehmigung. Parsonnet V, Bernstein AD. Cardiac Pacing in the 1980s: Treatment and Techniques in Transition. J Am Coll Cardiol 1983; 1:339–54)

Der Nuklearschrittmacher hatte gegenüber den Standardschrittmachern der späten 1960er-Jahre deutliche Vorteile, da die Stromquelle ein Leben lang hielt. Mitte der 1970er-Jahre wurden aber die neuen Lithiumbatterien leistungsfähiger und versprachen eine Funktion von 8 bis 10 Jahren bei kleinerer Größe als der Isotopengenerator (Abb. 6). Nuklearschrittmacher arbeiteten sehr zuverlässig, aber Fortschritte bei anderen Schrittmacherkomponenten, ein ungünstiges Kosten-Nutzen-Verhältnis und die Limitation der Systemlebensdauer durch Sondenprobleme relativierten die Vorteile. Schließlich führten Umweltbedenken, ein öffentliches Unbehagen in Bezug auf die nukleare Sicherheit und ein erheblicher bürokratische Mehraufwand zum Scheitern des Nuklearschrittmachers.

### Lithiumbatterien

Lithiumbatterien waren von Ingenieuren des Batterieherstellers Catalyst Research in Baltimore entwickelt worden und wurden 1970 von Greatbatch für den Einsatz in Herzschrittmachern produziert. Im August 1971 präsentierte *Wilson Greatbatch* seine ersten Daten einer neuen Festkörperbatterie auf der 9th International Conference on Medical and Biological Engineering in Melbourne, Australien. Die Zelle verwendete eine Lithiumanode und eine Jodkathode mit einem kristallinen Festkörperelektrolyt aus Lithiumjodid. Dies verhinderte die Erzeugung von Wasserstoffgas und ermöglichte somit zum ersten Mal die hermetische Abdichtung einer Batterie und eines Herzschrittmachers in einem geschweißten Gehäuse, das auch als indifferente Anodenelektrode in einem unipolaren System verwendet werden konnte^144^.

Die ersten Zellen (702A, 702B) waren im Designkonzept sehr einfach. Zwischen der Lithiumanode und dem Jod-Polyvinylpyridin(PVP)-Kathodenmaterial bildete sich eine Schicht aus Lithiumjodid, wodurch ein Festkörperelektrolyt in situ hergestellt wurde. Muster der 702C-Zelle der nächsten Generation wurden an alle interessierten Herzschrittmacherhersteller versandt. Die Weiterentwicklung (Modell 702E-Zelle) wies eine zentrale Lithiumanode auf, die vom Kathodenmaterial umgeben war. Dies führte zu einer deutlichen Verringerung der Zellimpedanz und einer Verlängerung der Lebensdauer. Proben dieses Zellmodells hatten ihre Entladung in Echtzeit nach etwa 17 Jahren abgeschlossen. Die Beschichtung der Anode mit reinem PVP vor der Zellkonstruktion führte zu einer weiteren dramatischen Abnahme des Innenwiderstands^145^.

Die erste Implantation eines Herzschrittmachers mit Lithiumbatterie (LEM Biomedica) erfolgte am 13. März 1972 in Italien bei einem Patienten mit Vorhofflimmern und langsamer Ventrikelfrequenz. Das System hatte eine einfache elektronische Schaltung (V00) und war noch in Epoxidharz eingegossen. Es musste nach weniger als 3 Monaten wegen Parasystolie wieder explantiert werden. Eine weitere Implantation erfolgte am 19. Juli 1972 im Arcispedale S. Anna in Ferrara (Italien) durch *Antonioli* bei einer Patientin mit totalem AV-Block, Batterieerschöpfung und epikardialer Elektrode. Sie erhielt eine neue endovenöse Sonde, die an einen Prototyp (LEM Cardiocron) mit einer 702C-Zelle angeschlossen wurde. Im Verlauf von 15 Monaten fiel die Stimulationsfrequenz von 74 min^−1^ auf 60 min^−1^ ab, was durch das Eindringen von Flüssigkeit in das Epoxidharz verursacht wurde. Das System wurde nach 30 Monaten aus hämodynamischen Gründen explantiert. Alle nachfolgenden lithiumbetriebenen Herzschrittmacher wurden außerhalb des Epoxidharzes in drei metallisch galvanisierte Schichten eingekapselt. Diese Herzschrittmacher mit neuer Lithiumzelle (702P) wurden erstmals im April 1973 verwendet^146^.

Zum Zeitpunkt dieser ersten Implantationen gab es einen allgemeinen Widerwillen, die etablierten Zink-Quecksilber- oder Kernenergiequellen aufzugeben. 1974 führte Medtronic einen mit Zink-Quecksilber betriebene Herzschrittmacher (Xytron) mit vorhofsynchronem und ventrikelinhibierendem Modus (VDD) ein, der eine Lebensdauer von 5 Jahren erreichen sollte. Ein Rückruf dieses Modells führte schließlich zur Aufgabe der Zink-Quecksilber-Stromquelle.

Die industrielle Produktion von Lithium-Herzschrittmachern in den USA wurde von dem früheren Medtronic Manager *Manny Villafaña* vorangetrieben, der nach einem Treffen mit *Greatbatch* die Firma Cardiac Pacemakers Incorporated (CPI, jetzt Boston Scientific, St. Paul, USA) gründete. Die damals erhältlichen Batterien waren die Greatbatch-702C-Zellen. Bis Januar 1973 wurden sieben asynchrone Lithium-Jodid-Impulsgeneratoren (CPI 702C) in den USA und Australien implantiert, von denen einer mehr als 10 Jahre hielt^147^.

Die nächste Generation mit doppelt umhüllter 702P-Zelle wurde in ungefähr 100 CPI-Herzschrittmachern implantiert, wobei eine Reihe von vorzeitigen Ausfällen berichtet wurde. Eine signifikante Verbesserung kam mit der 702E-Zelle mit Doppelanode. Die resultierende 80-g-Energiezelle hatte eine Nennkapazität von 2–3,5 Ah. Zwischen 1974 und 1976 wurde eine große Anzahl von Impulsgeneratoren (CPI Maxilith 1972, Teletronics 120 1974, Biotronik IDP-144 1975) mit diesen Zellen ausgestattet und erzielten hervorragende Ergebnisse. Allerdings war die große Energiezelle und damit der Impulsgenerator mit seinen eckigen Kanten anfällig für Drucknekrosen. Kleinere und dünnere Konstruktionen der Batteriehersteller Catalyst Research Corporation (CRC) und Wilson Greatbatch (WG) ermöglichten die Konstruktion von kleineren Impulsgeneratoren mit einer Kapazität von 2–3 Ah (Intermedics Thin Lith mit CRC 803/804 und CPI mit WG 755).

In **Deutschland** berichtete 1977 *Heinz Präuer*^148^ aus **München**, Rechts der Isar, über die 3‑Jahres-Ergebnisse mit 244 Lithiumschrittmachern unterschiedlicher Hersteller. Fünf Herzschrittmacher mussten wegen eines Elektronikfehlers ausgetauscht werden, keiner wegen Batterieversagen.

Sowohl Medtronic als auch Cordis setzten spät auf die Lithiumtechnologie. Erst 1977 wurden von Medtronic mehr als 3000 Herzschrittmacher vom Typ Xyrel mit zwei rundlichen Lithium-Jodid-Batterien (WG 742) auf den Markt gebracht. Der Cordis-Schrittmacher Lambda enthielt eine Alternativ-Technologie mit 3 Lithium-Kupfer-Sulfid-Batterien, die exklusiv von Dupont entwickelt worden waren. Nach seiner Einführung im Jahr 1976 kam es zu einer übermäßigen Anzahl unerwarteter vorzeitiger Ausfälle, die zu einem vollständigen Produktrückruf führten^149^. Mehrere andere Systeme auf Lithiumbasis wurden in Herzschrittmachern verwendet, die in den späten 1970er- und in den 1980er-Jahren hergestellt wurden. Ihre Verwendung ging im Laufe der Jahre zurück, und praktisch alle in den 1990er-Jahren hergestellten Herzschrittmacher verwendeten das Lithium-Jodid-PVP-System.

In den späten 1990er-Jahren erforderten Speicher zur Erfassung von Elektrogrammen, neue Sensoren der frequenzvariablen Therapie, leistungsstarke, kabellose Telemetriefunktionen und neue Therapien wie die kardiale Resynchronisation eine schnellere Bereitstellung höherer Energiemengen. Lithium-Jodid-Batterien wurden diesen Anforderungen nicht gerecht, sodass neue Batterietypen entwickelt wurden. Ein Beispiel dafür ist die von *Weiss* 1993 patentierte Hybridkathode, die aus Mischungen von Silber-Vanadiumoxid und Polycarbonmonofluorid besteht. Medtronic führte das Batteriesystem 1999 zur Behandlung von Vorhofflimmern ein. Im Jahr 2000 folgten die Systeme zur kardialen Resynchronisationstherapie mit 3 Elektroden und die normalen Herzschrittmacher. Die Gesamtzahl der mit Hybridkathodenbatterien betriebenen Implantate belief sich im Januar 2005 auf mehr als 100.000^150^.

## Bedarfsschrittmacher

Schon früh nach dem Beginn der Schrittmachertherapie mit starrfrequenten Systemen (VOO) kamen Bestrebungen auf, den Herzschrittmacher empfindlich zu machen für Herzaktionen aus der Kammer und den Vorhöfen. Bei Auftreten von Eigenrhythmus oder Extrasystolen bestand die Gefahr der Induktion lebensbedrohlicher tachykarder Rhythmusstörungen^151^. Die Herzschrittmachertherapie war so im Wesentlichen auf den permanenten totalen AV-Block beschränkt, intermittierende Störungen der AV-Überleitung oder Impulsbildung konnten nur unzureichend behandelt werden. Die Synchronisation von Vorhof und Kammer war nicht möglich.

Bereits der externe transkutane Herzschrittmacher, über den *Aubrey Leatham*^152^ 1956 in einer Modifikation berichtete, erfasste mittels EKG-Elektroden die Herzfunktion und reagierte darauf durch automatischen Beginn und Ende der Stimulation.

In **Deutschland** befasste man sich in **Düsseldorf** schon kurz nach der Erstimplantation mit der Entwicklung eines Bedarfsschrittmachers^153^. *Zacouto* hatte eine Kombination aus implantierbarem Schrittmacher und externem Gerät entwickelt, das Herzereignisse von der Körperoberfläche detektierte und den Herzschrittmacher durch induktive Hemmung ausschaltete. Beim Ausbleiben von Spontanaktionen sistierten die hemmenden Impulse und der Herzschrittmacher schaltete sich wieder ein. Die Lebensdauer der Batterie konnte so verlängert und eine elektrisch induzierte Parasystolie vermieden werden. Der Herzschrittmacher wurde durch *Sykosch, Effert* und *Pulver* 1962 getestet und 1963 erstmals bei einer jungen Patientin eingesetzt^154^. Mit dem schnellen Fortschreiten der Elektronik wurden in den folgenden Jahren allerdings einfachere Lösungen realisiert.

1965 stellte *Louis Lemberg*^155^ aus Miami, USA, einen vollständig implantierbaren Herzschrittmacher vor, der auf ventrikuläre Ereignisse reagieren konnte. 1968 veröffentlichte *Agustin Castellanos*^156^ die Ergebnisse von 10 Patienten mit implantierten Herzschrittmachern, bei denen das „Demand“-Prinzip (VVI) temporär für 2 Tage bis 2 Wochen getestet wurde. Eine weitere Variante war der von *Franco Donato* und *Luigi Denoth* aus Pisa, Italien, 1966^157^ vorgestellte R‑Zacken getriggerte („self-synchronising“) Herzschrittmacher (VVT), der bei auftretendem Eigenrhythmus den Impuls in die R‑Zacken und damit in die absolute Refraktärphase abgab. Die Störanfälligkeit des Demand-Schrittmachers gegenüber äußeren Impulsen wurde damit aufgehoben, der Vorteil der Stromersparnis ging allerdings verloren.

## Vorhofbeteiligte Stimulation

Konzepte einer vorhofsynchronen Kammerstimulation waren schon 1957 von *Folkman* und *Watkins* vor der ersten permanenten Herzschrittmacherimplantation entwickelt und im Tierexperiment angewandt worden^158^. Das erste System beim Menschen wurde 1963 von *David Nathan* aus Miami, USA, beschrieben und von der Cordis Corporation, USA, kommerziell hergestellt. Dieses System war unipolar und wurde durch Thorakotomie implantiert. Die atriale Elektrode diente nur der Wahrnehmung und übermittelte die P‑Welle an den Impulsgenerator mit Quecksilber-Zink-Zellen, der nach einer AV-Verzögerung einen ventrikulären Stimulus auslöste (VAT)^159^.

Die hämodynamischen Vorteile der vorhofbeteiligten Kammerstimulation konnten in den 60er-Jahren eindrucksvoll belegt werden. Hämodynamische Studien von *Gilmore* 1963 160 beim Hund und von *Benchimol* 1965^161^ und von *Samet* 1966^162^ am Patienten belegten den Nutzen der atrialen Synchronität für das Herzzeitvolumen. Trotzdem standen der allgemeinen Anwendung technische Schwierigkeiten und medizinische Bedenken gegenüber.

In **Deutschland** wurden die ersten Implantationen eines P‑Wellen-gesteuerten Schrittmachers 1964 in **Hamburg** von *Georg-Wilhelm Rodewald*^163^ und in **Düsseldorf** von *Jochen Sykosch*^164^ vorgenommen. Während *Rodewald* hierzu eine von *Lagergren*^165^ entwickelte Vorhofelektrode im rechten Herzohr platzierte, wurde in Düsseldorf die Implantation mittels Thorakotomie vorgenommen. *Sykosch* selbst beurteilte den P‑Wellen-gesteuerten Herzschrittmacher eher skeptisch.

„Ob diese Vorteile [Vorhofsynchronisation und Frequenzanstieg, *d. Verf.*] nicht mehr theoretischer Art sind, sei dahingestellt. Im eigenen Krankengut haben sich Nachteile dieser Konstruktion durch die prinzipbedingte Abhängigkeit von einer einwandfreien Vorhoffunktion bzw. von Vorhofrhythmustörungen ergeben … So haben wir in zwei Fällen eine vom Schrittmacher beantwortete, das heißt auf die Kammer übertragene Vorhoftachykardie beobachtet.“^166^

Die Vorhofelektrode war für viele Jahre nur über eine Thorakotomie sicher zu platzieren. Eine Alternative wurde 1965 von *Lagergren*^167^ in Zusammenarbeit mit der Firma Elema-Schönander, Schweden, entwickelt. Dabei wurde die Detektionselektrode über eine Mediastinoskopie in der Bindegewebsschicht zwischen der Hinterwand des Vorhofs und der Speiseröhre platziert. In **Deutschland** berichtete *Manfred Kleinert* aus **Hamburg-Harburg** 1976 über dieses Verfahren^168^. Bei transvenösen Implantationsverfahren mit vorgeformten Elektroden und bei der Verankerung der Elektrode innerhalb des Koronarsinus traten hohe Dislokationsraten und Fehlfunktionen auf^169^.

**Abb. 7:**
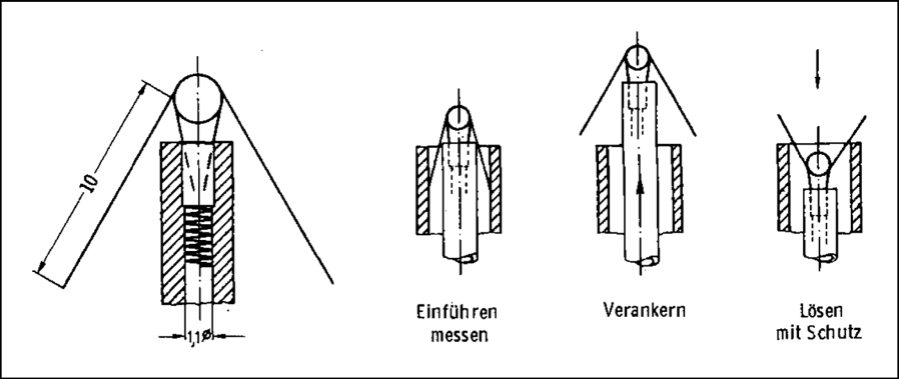
Widerhakenelektrode mit dem Prinzip der Einführung und der Entfernung mit Hilfe eines Führungskatheters. (© Georg Thieme Verlag KG, mit freundl. Genehmigung. Rosenkranz KA, Schaldach M. Transvenös-endokardiale Vorhofsteuerung von Schrittmachern. Dtsch Med Wschr 1971; 96:680–686)

Therapiesicherheit wurde erst 1968 mit Hilfe der in **Deutschland** von *Schaldach*^170^ entwickelten *Wire-hook-Elektrode* erreicht (Abb. 7), die über einen Führungskatheter implantiert werden musste und die erstmals von *Werner Porstmann*^171^ an der Charité in **Ost-Berlin**, DDR, erprobt wurde.

Die Elektrode wurde über einen vorgebogenen Führungskatheter über die V. jugularis oder V. cephalica zum rechten Herzohr vorgeschoben, bis der Elektrodenkopf etwa 3 bis 5 mm aus dem Führungskatheter herausragte. Bei guten Reizschwellen wurden durch weiteres Hinausschieben der Elektrode die Widerhaken freigegeben. Bis 1971 wurden an der Charitè bei 12 Patienten mit AV-Block und bei 3 Patienten mit SA-Block die Wire-hook-Elektroden implantiert und an einen VAT-Schrittmacher (IDP-64) oder an einen festfrequenten Vorhofschrittmacher (A00, IDP-44) der Firma Biotronik, Berlin, angeschlossen. In einer Nachbeobachtungszeit von bis zu 39 Monaten traten keine Dislokationen auf. Vorübergehende Sensingstörungen beschränkten sich auf die postoperative Phase. In einem erweiterten Follow-up von 36 Patienten bis 1974 traten 1 Perikarderguss, 2 Generatorinfektionen, 1 Dislokation und eine Sondenverlagerung auf. Die Elektrode wurde auch zur Vermeidung von Dislokationen bei stark dilatierten rechten Ventrikel vorgeschlagen und an 3 Fällen demonstriert^172^.

1971 berichteten *Rosenkranz* und *Schaldach*^173^ über die Ergebnisse bei 60 Patienten, die im Bergmannsheil **Bochum** mit einer Widerhakenelektrode im Vorhof versorgt wurden. Während der Beobachtungszeit von 27 Monaten trat keine Sondendislokation und kein Sondenbruch auf.

*Irnich* berichtete 1972 über die routinemäßige Nutzung der *Wire-hook-Elektrode* bei allen Patienten, bei denen Elektrodendislokationen aufgetreten oder zu erwarten waren. Dies hatte zu einer drastischen Reduzierung von Dislokationen von 22 % auf 8 % geführt. Ihm erschien diese Art der Implantation aber nicht risikolos, da durch den steifen 12-F-Führungskatheter Myokardperforationen auftreten konnten.

Als Weiterentwicklung stellte *Irnich* 1972 eine im Tierexperiment in **Aachen** getestete Hakenelektrode vor, die ohne Führungskatheter platziert werden konnte, indem durch einen inneren Mandrin ein beweglicher Bolzen nach vorne geschoben wurde, an dem dünne Häkchen aus Edelstahl befestigt waren. Die Elektrode konnte sowohl im Vorhof als auch im Ventrikel eingesetzt werden^174^.

Mitte der 1970er-Jahre kamen Elektroden mit feststehender Schraube auf den Markt, die in Zentren mit Erfahrung an aktiv fixierbaren Elektroden eingesetzt wurden. 1981 berichtete *Abderrahman Machraoui* aus dem Bergmannsheil in **Bochum** über 190 Patienten, bei denen 208 Schraubelektroden implantiert wurden, 147 im rechten Ventrikel und 61 im rechten Vorhof. Die Implantationsdauer im Vorhof- und Ventrikel war vergleichbar (54,7 ± 19 min vs. 56,0 ± 25 min), ebenso wie die Durchleuchtungszeiten (6,3 min vs. 8,1 min). Im Nachbeobachtungszeitraum von 23 Monaten trat nur bei einem Patienten mit „twiddler syndrome“ eine Dislokation der Vorhofelektrode auf. Bei einem Patienten musste die Ventrikelelektrode wegen eines Exitblocks repositioniert werden^175^.

Am Bergmannsheil **Bochum** war so bei 195 Patienten, die 1969–1986 einen VAT/DDD- oder AAI-Schrittmacher erhalten hatten, ein Vergleich verschiedener aktiv fixierbarer Vorhofelektroden möglich: die frühe Widerhakenelektrode (Biotronik IVE), die sichelförmige Ankerelektrode (Biotronik FH) und neue Schraubelektroden (Biotronik DY, Y2; Osypka VY). Die komplikationsfreien Überlebensraten im Vorhof zwischen Widerhaken- und Schraubelektroden waren über einen Zeitraum von 5 Jahren vergleichbar (88 % und 90 %) und verschlechterten sich nach 7 Jahren für die Widerhakenelektrode (79 % vs. 90 %)^176^. Der sichelförmige Verankerungsmechanismus hatte nach 5 Jahren eine deutlich höhere Dislokationsrate von > 20 % und die Widerhakenelektrode eine hohe Rate an ventrikulären Exitblockierungen^177^.

Einer der Entwickler einer feststehenden Schraube sowie des Mehrfach-Wendelprinzips^178^ war *Peter Osypka*^179^, Gründer der gleichnamigen Medizintechnik GmbH. Die Schraubelektrode stieß damals auch auf Skepsis. „Da haben mich die Chirurgen erst alle ausgelacht. Herr Osypka, im Herzen schraubt man nicht, haben die gesagt.“ Einer seiner Patienten, ein junges Mädchen, erhält 1977 als Erste solch eine Schraubelektrode. „Wir haben die dann jahrelang beobachtet, und das Kind ist gewachsen und gediehen und die Elektrode ist mitgewachsen und hat sich mitgedehnt.“ Einer der berühmtesten Träger einer solchen Konstruktion war Papst Benedikt XVI.

**Abb. 8:**
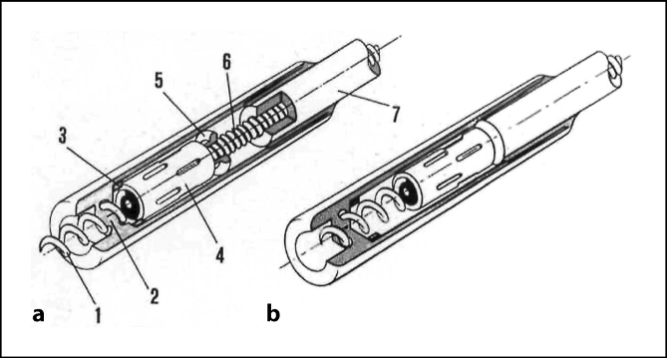
Prinzip der transvenösen Einschraubelektrode. **a** Spitze protrahiert, **b** Spitze zurückgezogen. *1* Wendelelektrode, *2* Vorderteil, *3* röntgendichter Ring, *4* Crimpbus, *5* Dichtring, *6* Leiterspulen, *7* Isolierschlauch. (© Wiley, mit freundl. Genehmigung. Bisping HJ, Kreuzer J, Birkenheier H. Three-year clinical experience with a new endocardial screw-in lead with introduction protection for use in the atrium and ventricle. PACE 1980; 3:424–435)

1976 berichtete *Hans-Jürgen Bisping*^180^ aus **Aachen** von ersten Ergebnissen aus Tierversuchen über eine neue, einfach handhabbare Schraubelektrode^181^ und 1977 zusammen mit *Manfred Kleinert* aus **Hamburg-Harburg** über erste klinische Ergebnisse^182^. Im Gegensatz zur feststehenden Schraube, bei der Wendel und Stylet gemeinsam mit dem Sondenkörper gedreht werden mussten, konnte bei dieser Elektrode durch Rechtsdrehung des Steckerstiftes die Wendel aus der Schutzhülle ausgefahren und dabei in der zuvor gewählten Position verankert werden. Bei Drehung entgegen dem Uhrzeigersinn konnte die Schraube wieder eingefahren werden (Abb. 8). Die Ergebnisse von 127 Implantationen bei dilatiertem Ventrikel und von 23 im Vorhof wurden 1980 mitgeteilt. Die Elektroden waren einfach zu platzieren und zeigten hervorragende Reizschwellen (Vorhof 0,62 V, Ventrikel 0,59 V bei 0,5 ms) und Sensingwerte (Vorhof 3,6 mV; Ventrikel 12 mV). Während einer Nachbeobachtungszeit von 900 Monaten traten bei 2 Patienten (1,6 %) Exit-Blöcke auf^183^. Damit waren die Komplikationsraten so niedrig, dass sie nicht weiter einer vorhofbeteiligten Stimulation im Wege standen.

1977 stellte *Kleinert* aus **Hamburg-Harburg** seine Ergebnisse einer neuen atraumatischen Vorhofelektrode bei 53 Patienten vor. Die Elektrode bestand aus Platin-Iridium und hatte eine aktive Oberfläche von ca. 11 mm^2^. Der distale Teil bildete eine J‑Form, die durch Einführen eines Stylets begradigt werden konnte. Am kurzen Schenkel des J waren in einen Winkel von 90° drei Reihen von elastischen Silikonkautschukankern angebracht, um die Elektrode im rechten Herzohr zu fixieren. Die durchschnittliche P‑Wellen-Amplitude betrug 5,17 mV, die Reizschwelle 1,13 V bei einer Impulsbreite von 1 ms und damit höher als im Ventrikel. Exitblockierungen traten nicht auf, Dislokationen in 5,6 %^184^.

Neben Vorhofschrittmachern (AAI) kamen nun auch vermehrt 2‑Kammer-Schrittmacher zum Einsatz. Bei einer vorhofsynchronen Kammerstimulation mit einem VAT-System, das bei totalem AV-Block eingesetzt wurde, konnte der Vorhof nicht stimuliert und Kammeraktionen nicht wahrgenommen werden, mit der Gefahr proarrhythmischer Stimulationen. Das von *Barouh Berkovits* aus Miami, USA, 1969 vorgestellte AV sequentielle Demand-System (DVI), war auf Vorhofebene blind und konnte deshalb nur bei überwiegender Sinusknotenerkrankung eingesetzt werden. *Cesar Castillo*^185^berichtete 1971 über 7 Patienten, die temporär mit einem externen bifokalen Demand-Schrittmacher versorgt wurden und über 3 Patienten, bei denen ein System dauerhaft implantiert wurde, mit zufriedenstellenden Ergebnissen. *Greatbatch* patentierte 1969 den VDD-Schrittmacher^186^, eine Weiterentwicklung des VAT-Systems, das jetzt Ventrikelaktionen wahrnehmen konnte, und *Berkovits* 1971 den DDI-Schrittmacher^187^, der zwar Vorhof und Kammer wahrnehmen und stimulieren, aber keine vorhofsynchrone Ventrikelstimulation durchführen konnte. Der Einsatz dieser 2‑Kammer-Schrittmacher blieb also auf die jeweiligen Rhythmusstörung, AV-Blockierungen beim VDD-System und Sinusknotenerkrankung beim DDI-System, beschränkt.

Zur Untermauerung des klinischen Nutzens eines solchen DDI-Systems, das in **Düsseldorf** bei 3 Patienten implantiert worden war, führten *Marx, Sykosch, Goebel und Arnold* aus der Chirurgischen Klinik und dem Physiologischen Institut der Universität Düsseldorf, sowie der Chirurgischen Abteilung des Dominikus-Krankenhauses Düsseldorf tierexperimentelle hämodynamische Untersuchungen an Hunden durch. In 11 Versuchen fiel im Mittel der periphere Blutdruck beim Übergang vom Vorhof- auf Ventrikelstimulation von 161 auf 133 mm Hg und der linksventrikuläre Druck von 132 auf 109 mm Hg ab. Die Druckanstiegsgeschwindigkeit nahm um 19,5 % ab, ebenso das Schlagvolumen von 16,6 auf 13,3 ml und das Herzminutenvolumen von 2,20 auf 1,73 l pro min. Damit konnte nachgewiesen werden, dass mit dem atrioventrikulären bifokalen Demand-Schrittmacher eine neue effektive Behandlungsmethode für sinuatriale Überleitungsstörungen eingeführt wurde^188^.

**Abb. 9:**
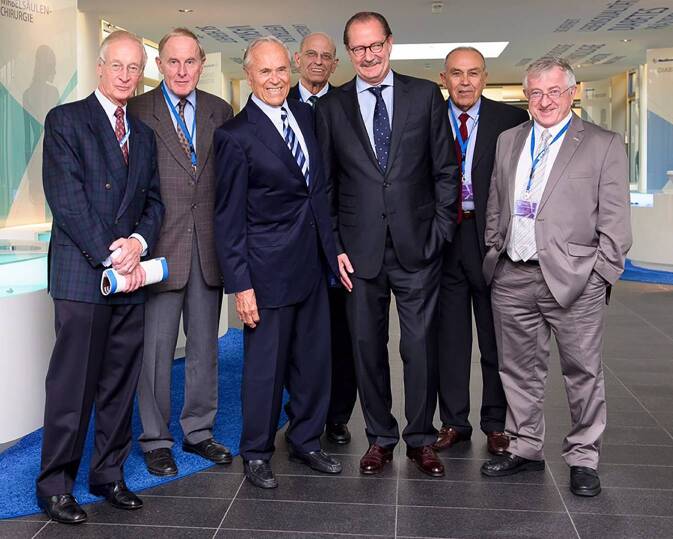
Jubiläumsfeier 2011 in der Universitätsklinik Düsseldorf anlässlich des 50. Jahrestages der ersten Schrittmacherimplantation in Deutschland; *von links nach rechts*: Prof. Ludger Seipel, Tübingen; Prof. Christoph Reidemeister, Essen; Prof. Heinz-Joachim Sykosch, Düsseldorf; Prof. Hagen Schulte, Düsseldorf; Prof. Joachim Winter, Düsseldorf; Prof. Garcia; Prof. Hermann Funke, Bonn. (Quelle: Prof. Joachim Winter, Düsseldorf)

Der Durchbruch zu einem optimalen 2‑Kammer-System, das bei beiden Rhythmusstörungen eingesetzt werden konnte, gelang in **Deutschland**. Bereits 1975 stellten *Irnich* und *de Bakker* vom Helmholtz-Institut für Biomedizinische Technik an der RWTH **Aachen**^189^ das Konzept eines optimalen 2‑Kammer-Schrittmachers vor, der eine vorhofsynchrone sequentielle Stimulation ermöglichte. Der Schrittmacher führte bei AV-Blockierungen eine P‑Wellen getriggerte Vorhof- und Ventrikelstimulation durch und bei Sinusknotenerkrankung eine Vorhofstimulation sowie eine Ventrikelinhibition bei intrisischer Überleitung. Das Problem hoher Stimulationsfrequenzen bei auftretendem Vorhofflimmern oder -flattern wurde ebenso berücksichtigt (automatische Frequenzbegrenzung auf 75 min^−1^ bei Vorhoffrequenzen über 180 min^−1^) wie das Problem schrittmacherinduzierter Reentry-Tachykardien. Den ersten universellen Vorhof- und Kammer-Bedarfsschrittmacher (DDD), wie er heute noch Verwendung findet, stellte 1978 *Hermann Funke*^190^ aus **Bonn**^191^ vor, nachdem er zuvor das Patent hierfür angemeldet hatte^192^. Dieser Schrittmacher arbeitete multiprogrammierbar auf der Grundlage eines Computerchips, wie er kurz zuvor in einem der ersten überall verfügbaren Homecomputer (Commodore C64®, Commodore Business Machines Inc., West Chester, PA, USA) erhältlich wurde. Anlässlich des 50. Jahrestages der ersten Herzschrittmacher-Implantation in Deutschland trafen sich 2011 wichtige Pioniere der Therapie in Düsseldorf (Abb. 9).

Bereits in den 1970er-Jahren konnte *Giovanni Antonioli* aus Ferrara, Italien, zeigen, dass eine vorhofgesteuerte Ventrikelstimulation mit einem **Ein-Elektrodensystem** möglich war und atriale Elektrogramme über eine unipolare Floating-Elektrode im mittleren bis oberen rechten Vorhof detektiert werden konnten. Im Jahr 1980 wurden implantierbare Prototypen mit angepassten VDT-Impulsgeneratoren konstruiert. Zwei solche Einheiten wurden im August und November 1980 implantiert. 1982 wurde eine neue AV-Elektrode mit einer atrialen Ringelektrode von 43 mm^2^ Oberfläche entwickelt, die 13 cm von der ausschraubbaren ventrikulären Spitze entfernt angeordnet war. Zwischen Oktober 1985 und März 1989 wurde diese unipolare AV-Elektrode in Italien bei mehr als 250 Patienten mit komplettem AV-Block implantiert und an den VDD-Schrittmacher der Firma Cardiac Pacemakers, USA (Modell 910, Ultra II) angeschlossen. Synchronisationsfehler wurden in weniger als 3 % der Patienten beobachtet. In den frühen 1980er-Jahren wurden drei verschiedene Konfigurationen atrialer Ringe verglichen^193^.

*Bernd Nowak* aus **Frankfurt** berichtete 2001 über die in den 1990er-Jahren durchgeführten Untersuchungen bei 2000 Patienten mit bipolaren VDD-Schrittmachern und einer maximal empfindlich programmierten atrialen Wahrnehmungsschwelle, die mittels Langzeit-EKG eine AV-synchrone Stimulation von im Mittel über 99 % aufwiesen. In randomisierten Studien zeigte die VDD-Implantation gegenüber der DDD-Implantation eine signifikante Verkürzung der Operations- und der intraoperativen Durchleuchtungszeit^194^ sowie eine Reduktion perioperativer Komplikationen^195^.

1992 demonstrierte *Maria Bongiorni* aus Pisa, Italien, erstmals unter Verwendung einer konventionellen flottierenden VDD-Elektrode eine atriale wandferne Stimulation, jedoch mit hohen Reizschwellen und häufigen Zwerchfellstimulationen^196^. Es wurden speziell vorgeformte Elektroden entwickelt, die einen stabilen Wandkontakt des atrialen Dipols ermöglichen sollten. *Carsten Israel* aus **Bochum** berichtete 1999 über eine erfolgreiche Stimulation über atriale Ringe einer präformierten VDD-Elektrode^197^. Es kam jedoch im Verlauf von 1 Jahr zu einem Reizschwellenanstieg bei 5,0 V von 0,22 ms auf 0,65 ms. *Jan C.J. Rees* aus Rotterdam, Niederlande, berichtete 2000 über eine zuverlässige atriale Stimulation mit einer dreipoligen atrialen Vollringelektrode in 93 % der Patienten bei der Implantation. Allerdings war diese nach 3 Monaten bei hohen Reizschwellen ohne Zwerchfellzucken nur noch bei 54 % der Patienten möglich^198^. Eine neuartige Impulskonfiguration untersuchte *Wolfgang Hartung* aus **Magdeburg** 1998. Durch die Abgabe eines „overlapping biphasic impulse“ (OLBI) zwischen den atrialen Elektroden wurde eine stabilere atriale Stimulation erreicht zusammen mit einer Verkürzung der intra- und interatrialen Leitungszeiten^199^.

All diese Ansätze konnten sich letztlich gegenüber einem DDD-System mit separater Vorhofelektrode nicht durchsetzen. Bereits 1984 schlug eine Task Force der NASPE^200^,^201^ vor, dass eine DDD-Stimulation in 60−80 % aller Fälle indiziert sei, heute beträgt der Anteil in Deutschland 76,5 % plus 6,2 % CRT-Systeme (3-Kammer-Schrittmacher). Der Anteil der VDD-Schrittmacher in Deutschland lag zuletzt nur noch bei 0,1 %^202^.

## Sensorgesteuerte, frequenzvariable Stimulation

*Astrand* und *Landegren* führten am Karolinska Institut in Stockholm bereits 1965 bei Patienten mit totalem AV-Block und externem Ventrikelschrittmacher Belastungsuntersuchungen auf dem Fahrrad durch, bei denen sie die Stimulationsfrequenz bis auf 105 min^−1^ anhoben. Dabei konnten sie zeigen, dass mit steigender Herzfrequenz die maximale Leistung und die Sauerstoffaufnahme zunahmen und der Blut-Laktatspiegel abfiel^203^.

Zehn Jahre später (1975) konnte *Ingvar Karlof* am selben Institut bei 25 Patienten mit komplettem AV-Block in hämodynamischen Vergleichsuntersuchungen zwischen festfrequenter, vorhofsynchroner und vorhofunabhängiger frequenzvariabler Kammerstimulation den Nutzen der atrialen Synchronität und Frequenzvariation für das Herzzeitvolumen nachweisen. Dabei konnte er erneut zeigen, dass bei körperlicher Anstrengung eine Erhöhung der Herzfrequenz auch ohne atriale Synchronität zu einer Zunahme des Herzzeitvolumens führt^204^. Diese Erkenntnis war nach *Furman*^205^ die intellektuelle Grundlage für die spätere Entwicklung von Sensoren zur Frequenzmodulation.

Zu Beginn der 1980er-Jahre begann eine intensive Suche nach Sensoren, die es ventrikulären Schrittmachern ermöglichten, die Stimulationsfrequenz als Reaktion auf physiologische Anforderungen zu ändern. Dreizehn Sensoren für die frequenzadaptive Stimulation wurden in klinischen Untersuchungen am Menschen getestet. Durchgesetzt hat sich der am wenigsten physiologische Aktivitätssensor, der eine Standardelektrode verwendete und schnell auf körperliche Betätigung reagierte, aber nicht mit dem Belastungsniveau korrelierte und nicht auf emotionale Reize oder Fieber. Drei andere Sensoren sind heute noch erhältlich: Atemminutenvolumen, Closed-Loop-Stimulation und zentralvenöse Temperatur^206^.

Das erste frequenzvariable System, das von *Cammilli* 1976 implantiert (LEM Biomedica, Italien) wurde, reagierte auf den ***pH-Wert des Blutes***, der bei körperlicher Belastung abfällt. Eine Ir/IrO2-Elektrode auf atrialer Ebene erfasste den pH-Unterschied im Vergleich zu einer Elektrode am Herzschrittmachergehäuse. *Cammilli* berichtete über 7 Patienten mit diesem Schrittmacher, 4 funktionierten über ein Jahr angemessen, 3 reagierten nicht^207^. Neben der unzureichenden Zuverlässigkeit hat auch die eingeschränkte Biokompatibilität der Silberchloridelektrode die weitere Entwicklung beendet.

In **Deutschland** veröffentlichte *Funke* 1975 das Konzept eines frequenzvariablen Herzschrittmachers und die tierexperimentellen Ergebnisse beim Hund, der über die Messung ***thorakaler Atemschwankungen*** mittels eines intrapleuralen Drucksensors gesteuert wurde^208^.

Die ***Atemfrequenz*** als einfacher biologischer Parameter war schon 1966 von *Voukydis* und *Krasner* aus Boston, USA, als Sensor vorgeschlagen und im Tierexperiment und an Probanden getestet^209^und ein ähnlicher Ansatz mit Atemfrequenzerfassung über Druckänderungen 1980 von *Ionescu* beschrieben worden^210^.

Anfang der 80er-Jahre griff *Paolo Rossi* aus Novara, Italien, das Konzept des atmungsgesteuerten Schrittmachers erneut auf, indem er die Atemfrequenz über transthorakale Impedanzmessung zwischen der Sondenspitze des Schrittmachersystems und einer subkutanen Zusatzsonde bestimmte. 1983 berichtete er über die Ergebnisse atmungsgesteuerter Schrittmacherstimulation bei 67 Patienten mittels zweier vollständig implantierter Systeme (Biotec, Italien)^211^. 1988 waren bereits ca. 5000 atemgesteuerte Herzschrittmacher in Europa implantiert, und *Rossi* stellte die klinischen und physiologischen Daten von 143 Patienten vor, 121 mit ventrikulärer und 22 mit atrialer Stimulation. Die physiologische Empfindlichkeit des Stimulationssystems war bei 70 % der Patienten ausgezeichnet (bis zur Erschöpfung), bei 20 % sehr gut (bis zur anaeroben Schwelle) und ohne Zusammenhang zum O_2_-Atemäquivalent bei 10 % der Patienten. Die Inzidenz der Fehlfunktion des Systems betrug weniger als 8 %^212^. Im Langzeitverlauf war das System aber nicht zuverlässig genug, und es traten Komplikationen mit der Subkutanelektrode auf.

Mit dem Ziel einer physiologischeren Frequenzantwort verfolgten in **Deutschland** *Lampadius*^213^ 1985^214^und *Alt*^215^ 1987^216^aus **München** den Ansatz, die Herzfrequenz nicht allein durch die Atemfrequenz, sondern durch das ***Atemminutenvolumen*** zu steuern.

*Tibor Nappholz*^217^ und *Tony Simmons*^218^ zeigten 1985 und 1986 für intravaskuläre Elektroden eine hohe Korrelation zwischen Atemzugvolumen und gemessener elektrischer Impedanz. Die Arbeiten mündeten in der Entwicklung eines Atemminutenvolumen-gesteuerten Schrittmachersystems^219^, das über den Ring einer bipolaren Elektrode niedrigenergetische Impulse gegen das Schrittmachergehäuse als indifferente Elektrode abgab (Teletronics, Australien). 1988 berichtete *Harry Mond* aus Melbourne, Australien, über die Ergebnisse bei 12 Patienten, 6 mit AV-Knoten-Ablation, 4 mit totalem AV-Block und 2 Sick-Sinus-Patienten, die einen leistungsadäquaten Frequenzanstieg zeigten^220^.

Trotzdem war das Atemzugvolumen mit Totzeiten um 20 s nur ein mittelschnell reagierender Parameter, zu dem sich noch technische Totzeiten addierten. Diese Limitationen machten eine Steuerung mittels Atemminutenvolumen erst ab einem mittleren Lastbereich möglich. Eine partielle Kompensation konnte durch die Kombination mit einem schnell reagierenden Sensor wie der Aktivität erreicht werden und wurde in 1‑ und 2‑Kammer-Schrittmachern realisiert (Teletronics, Australien; Medtronic, USA). Gegenwärtig gibt es von zwei Herstellern (Boston Scientific, USA und MicroPort, China) DDDR-Schrittmacher mit einem dualen Sensor inkl. Atemminutenvolumen-Sensor.

In **Deutschland und Österreich** wurde die ***zentralvenöse Bluttemperatur*** als Biosensor des Stoffwechselbedarfs experimentell und klinisch untersucht. *Georg Csapo* aus **Bad Krozingen** beschrieb die Veränderungen während körperlicher Belastung und präsentierte die Ergebnisse 1976 in Tokio auf dem VIII. Weltkongress Kardiologie^221^. Nach seinem tragischen Tod wurde sein Patent^222^ nicht mehr weiterentwickelt. 1982 berichtete *Axel Laczkovics* aus **Wien** über seine Ergebnisse^223^ und *Jerry Griffin* aus Houston beschrieb 1983 beim Hund eine nahezu lineare Beziehung zwischen zentralvenöser Temperaturänderung und Belastung^224^. 1984 testete *Laczkovics* im Selbstversuch und bei einem Schrittmacherpatienten eine temporäre Thermistorsonde im rechten Vorhof. Er fand signifikante Temperaturanstiege bis 1,35 °C bei Laufbandgeschwindigkeiten von 6 und 10 km/h, während bei einer langsameren Geschwindigkeit von 3 km/h kein eindeutiger Temperaturanstieg zu verzeichnen war. Gleichzeitig berichtete er über den ersten temporären Schrittmacher (Biotronik), der bei einem Probanden an eine temporäre Thermistorsonde angeschlossen wurde und der bei einer Laufbandbelastung von 6 km/h eine sehr gute Übereinstimmung von Temperaturänderung, eigener Herzfrequenz und Stimulationsfrequenz zeigte^225^. Eine gute Korrelation zwischen Belastung und zentralvenöser Bluttemperatur fand 1984 auch *Alt* aus **München**^226^und stellte 1986 seine Untersuchungen bei 31 Probanden vor^227^.

Drei Hersteller hatten temperaturgesteuerte Herzschrittmacher in Studien getestet (Biotronik, Intermedics, Cook Pacemaker). Probleme ergaben sich mit der Langzeitstabilität des Temperatursensors und durch den initialen Temperaturdip bzw. den verzögerten Temperaturabfall nach Belastung, auch durch den unzureichenden Temperaturanstieg bei herzinsuffizienten Patienten und bei geringer Belastungsintensität. Derzeit wird nur beim Leadless Pacer (St. Jude/Abbott) die Temperatur zur Frequenzsteuerung benutzt.

**Abb. 10:**
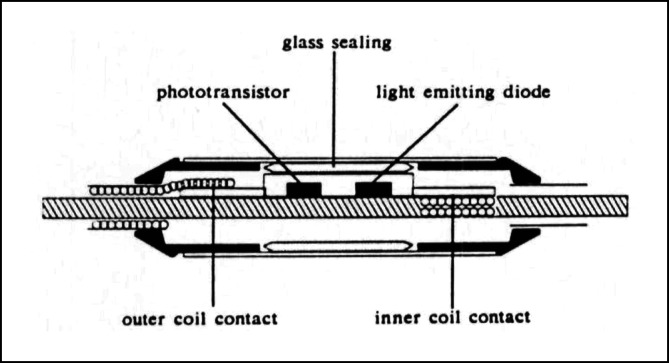
Querschnitt des optischen Sensors zur Messung der zentralvenösen Sauerstoffsättigung. (© Wiley, mit freundl. Genehmigung. Stangl K, Wirtzfeld A, Heinze R, Laule M. First experience with an oxygen controlled pacemaker in man. PACE 1988; 11:1882–1887)

Als ein weiteres physiologisches Prinzip der Frequenzsteuerung wurde in **Deutschland** 1981 von *Alexander Wirtzfeld*^228^ aus **München** die Messung der ***zentralvenösen Sauerstoffsättigung*** (SO_2_) vorgeschlagen^229^. Unter Verwendung eines speziellen optischen Sensors und einer Leuchtdiode, die in der Schrittmacherelektrode integriert war (Abb. 10), konnte der Abfall der Sauerstoffsättigung während körperlicher Belastung gemessen werden. Die Erwartung war, dass die SO_2_-Messung als *Closed-Loop-System* funktioniert: Durch die Änderung der Stimulationsfrequenz verbessert sich das Herzzeitvolumen und die arteriovenöse Sauerstoffdifferenz nimmt ab, was wiederum zu einer negativen Rückkopplung auf den SO_2_-Wert führt. In einem Multisensorvergleich bei 12 gesunden Probanden berichtete er 1988 zusammen mit *Karl Stangl* über eine hervorragende kurzzeitige Modulation der Herzfrequenz mit einem SO_2_-Sensor, der sich im rechten Vorhof befand. Wegen der geringeren Sensitivität des Sensors bei höherer Belastung schlug er gleichzeitig eine Kombination mit einem Temperatursensor vor^230^. 1988 stellte *Stangl* die ersten Implantationen eines SO_2_-gesteuerten Herzschrittmachers (Siemens Elema) beim Menschen vor^231^. Zur Stabilität dieses Designs über einen längeren Nachbeobachtungszeitraum liegen keine Informationen vor.

Obwohl die Messung der zentralvenösen Sauerstoffsättigung am besten geeignet war, die physiologischen Bedingungen einer adäquaten Frequenzsteigerung abzubilden, erreichte keines der Systeme in klinischer Erprobung der Firmen Siemens und Medtronic eine Marktzulassung.

Nach *Harry Mond* aus Melbourne, Australien, leitete 1983 der ***Aktivitätssensor***^232^ die Ära der modernen frequenzadaptiven Stimulation ein und bleibt der bei weitem am häufigsten in Herzschrittmachersystemen eingebaute Sensor^233^. Mittels eines piezoelektrischen Kristalls, der an der Innenseite der Oberfläche des Herzschrittmachergehäuses angebracht ist, werden Vibrationen oberhalb einer programmierbaren Schwelle gezählt und zur Steuerung der Stimulationsfrequenz genutzt.

1983 berichtete *D. P. Humen* aus London, Ontario, Canada, auf dem VII. Weltkongress Cardiac Pacing in Wien über die ersten Ergebnisse dieses Sensorschrittmachers, der bei 5 Probanden epikutan auf der Thoraxwand angebracht war und bei 2 Patienten implantiert wurde. Bei den Probanden ergab die Auswertung des Holter-EKGs eine gute Übereinstimmung mit der Sinusknotenfrequenz, wobei die Maximalfrequenz des Schrittmachers auf 120 min^−1^ begrenzt war, der Sinusrhythmus aber bis auf 180 min^−1^ anstieg. Ein Patient mit Sinusknotenerkrankung erhielt einen AAI-Schrittmacher, der andere mit AV-Block einen VVI-Schrittmacher. Unter Laufbandbelastung zeigte der Sinusknotenpatient einen mit gesunden Probanden vergleichbaren Frequenzanstieg und der AV-Block-Patient, verglichen mit VVI 70 min^−1^, eine deutlich verlängerte Belastungstoleranz^234^. 1986 stellte *Fred Lindemans* von Medtronic die Ergebnisse einer Multicenterstudie mit 222 Patienten vor und zeigte unter frequenzvariabler Stimulation eine Verbesserung der körperlichen Leistungsfähigkeit und Abnahme der Symptome^235^.

Der aktivitätsgesteuerte Herzschrittmacher reagierte nicht auf physiologische Veränderungen, die mit minimalen Körperbewegungen einhergingen. Insbesondere korrelierte die Frequenzanpassung nicht mit dem Belastungsniveau. So war die Stimulationsfrequenz beim Treppe steigen niedriger als beim Treppe heruntergehen. Die Stimulationsfrequenz änderte sich während des Laufbandtrainings nicht, wenn nur die Steigung erhöht wurde und die Geschwindigkeit konstant blieb, da die Schrittmacherfrequenz von der Schrittfrequenz abhängig war^236^. Bei einem Patienten mit Beinprothese und aktivitätsgesteuertem Herzschrittmacher blieb jeglicher Frequenzanstieg unter Belastung aus, weshalb ich meinen Vortrag auf dem internationalen Rate Responsive Kongress 1987 in München^237^ mit der Bemerkung schloss: „Activitrax and artifical leg don’t go together.“

Der Herzschrittmacher der Firma Siemens verwendete einen ähnlichen piezoelektrischen Kristallsensor zur Aktivitätserfassung. Eine Besonderheit war die Bereitstellung von Frequenzhistogrammen und Autoprogrammierbarkeitsoptionen, um eine erforderliche Stimulationsfrequenz für eine Aktivität zu erreichen.

In **Deutschland** berichtete 1989 *Eckhard Alt* aus **München** über die Verwendung von drei miniaturisierten piezoresistiven ***Beschleunigungsmessern*** der Firma Endevco, USA, die in der Automobilindustrie zur Steuerung von Airbags eingesetzt wurden. Durch Verwendung eines Tiefpassfilters wurde der Einfluss unerwünschter externer Vibrationen reduziert. Die erfassten Beschleunigungskräfte wurden mit der Intensität der Arbeitsbelastung während täglicher Aktivitäten in Beziehung gesetzt und konnten von Fremdschwingungen gut unterschieden werden^238^.

Bei den Schrittmachersystemen wurde der Sensor direkt auf die Platine verlagert und war damit nicht mehr anfällig für direkten Druckeinfluss von außen. Verwendet wurden piezoelektrische Kristalle (Intermedics, USA) oder integrierte Silikon-Beschleunigungsmesser (Cardiac Pacemakers, USA). Der Niederfrequenz-Beschleunigungsalgorithmus wurde auch in den Herzschrittmachern von Biotronik, Berlin, mit piezoelektrischer Erfassung verwendet. Alle Aktivitätssensoren waren kommerziell erfolgreich und werden weiterhin als häufigster Sensor eingesetzt.

Über einen ***mechanischen Sensor***, der ein Neigungsschalterprinzip enthält, berichtete *Alt* 1988. Hiermit konnte eine Haltungs- und Aktivitätserkennung erreicht werden, indem eine Metallkugel über eine Scheibe mit Ein‑/Ausschaltern bewegt wurde. Die Haltungserkennung ermöglichte eine niedrigere Stimulationsfrequenz während des Schlafs und eine schnelle Frequenzanpassung beim Einnehmen einer aufrechten Haltung^239^.

Ein ***Gravitationsbeschleunigungssensor*** erkannte die vertikale Beschleunigung beim Gehen und Laufen im Unterschied zu Vibrationen. Der Herzschrittmacher (Sorin, Italien) wurde seit 1990 implantiert und enthielt einen zylindrischen, hermetisch verschlossenen Metallbehälter mit einem zentralen Quecksilbertröpfchen, das sich bei Anstrengungen verformte. Die Ergebnisse wurden 1992 von *Maria Bongiorni* aus Pisa, Italien, veröffentlicht^240^. Eine spätere Version von Siemens aus dem Jahr 1994 verwendete eine frei bewegliche Magnetkugel in einem elliptischen Hohlraum, der von zwei Kupferdrahtspulen umgeben und auf der Leiterplatte montiert war. Die Bewegung des Balls erzeugte elektrische Signale, die bei Aktivität zu Änderungen der Stimulationsfrequenz führten^241^.

Die ***maximale endokardiale Beschleunigung*** wurde über einen Beschleunigungsmesser in der Spitze der rechtsventrikulären Stimulationselektrode während der ventrikulären Kontraktion gemessen. Damit konnte auch der Schluss der atrioventrikulären Klappen während der isovolumetrischen Kontraktionszeit erfasst werden. Der Sensor konnte Veränderungen bei emotionalem Stress und körperlicher Betätigung erkennen. Die ersten Sonden wurden 1989 von Sorin (Sorin, Italien) implantiert und die Ergebnisse von *Langenfeld* aus **Würzburg** 1998 publiziert^242^. Die Notwendigkeit einer speziellen Elektrode mit miniaturisiertem Piezo-Kristall schränkte die Akzeptanz des Sensors ein.

Ein neu entwickelter ***dreiachsiger Beschleunigungssensor*** wurde für den sondenlosen ventrikulären Herzschrittmacher entwickelt (Medtronic, USA). Der Sensor steuert nicht nur die frequenzadaptive Stimulation, sondern erkennt auch die atriale Kontraktion und ermöglicht somit eine vorhofsynchrone ventrikuläre Stimulation (VDD). Allerdings nahm die Erkennung des atrialen Signals während der Aktivität mit steigender Herzfrequenz ab, was zum Verlust der atrialen Synchronität führte^243^ (siehe auch Kapitel „Sondenlose Herzschrittmacher“).

Ein biologischer Sensor, der die Veränderungen des ***QT-Intervalls*** bei körperlicher Betätigung ausnutzte, wurde 1981 von *Anthony Rickards* aus London, England, entwickelt. Das QT-Intervall verkürzt sich bei körperlicher Anstrengung und psychischem Stress. Ungefähr die Hälfte dieser Verkürzung wird durch die Erhöhung der Herzfrequenz verursacht^244^.

Der QT-Schrittmacher (Vitatron, Niederlande) konnte über die normale Stimulationselektrode die T‑Welle der stimulierten ventrikulären Aktion, die Stimulus-T-Zeit, ausmessen und als Sensor für die Frequenzadaptation benutzen. Die Limitationen des Systems waren dadurch begründet, dass die Beziehung zwischen Herzfrequenz und Stimulus-T-Intervall inter- und intraindividuell erheblich variieren konnte. Obwohl das Ausmaß der Frequenzanpassung bei diesem Herzschrittmacher mit der Arbeitsbelastung zusammenhing, trat die QT-Zeit-Verkürzung mit einiger Verzögerung auf und konnte während der Erholung zu Tachykardien führen. Der QT-Schrittmacher war über eine Dekade im Einsatz, bevor nach mehrfachen Modifikationen der Algorithmen die Produktion eingestellt wurde.

Die Korrelation zwischen ***Druckanstiegsgeschwindigkeit dP/dt*** im rechten Ventrikel und Herzfrequenz wurde von *Bennett* 1985 bei chronisch implantierten dP/dt-Systemen in Hunden untersucht^245^. Ein implantierbarer Herzschrittmacher (Medtronic, USA) benötigte eine spezielle Drucksensorelektrode auf piezoelektrischer Basis. 1987 wurden von S*harma* 246und von *Richard Sutton*, London, England,^247^ die ersten Implantationen im Humanbereich durchgeführt. Da viele Fragen, wie z. B. die Auswirkung einer Rechtsherzinsuffizienz und die Langzeitrobustheit des Sensors, unbeantwortet blieben, wurden Implantationen des Systems nur begrenzt durchgeführt.

Das Konzept des ***ventrikulären Depolarisationsgradienten*** wurde erstmals 1934 von *Wilson* beschrieben. Der Gradient wird aus der Integration des stimulierten evozierten QRS-Komplexes abgeleitet und verringert sich unter körperlicher Belastung^248^. Die Verwendung dieses Signals zur frequenzadaptiven Stimulation wurde 1987 von *Callaghan* vorgeschlagen^249^. Der implantierte Herzschrittmacher (Teletronics, USA) zeigte eine schnelle Änderung der Stimulationsfrequenz von 10 s nach Belastung. Allerdings trat bei einer Nachuntersuchung von 123 Patienten ein signifikantes Versagen bei der Frequenzanpassung (14 %) und der Schwellenerkennung (11,2 %) auf, zwei Einheiten mussten wegen unangemessener Frequenzreaktion explantiert werden^250^. Das Konzept konnte sich im Markt nicht durchsetzen. Dafür begründete ein Vorteil des Algorithmus, dass mit ihm ein Exitblock erkannt und durch Erhöhung der Energieabgabe behoben werden konnte, die *Autocapture-Funktion* späterer Herzschrittmacher.

Sofortige Schlag-zu-Schlag-Änderungen des ***rechtsventrikulären Präejektionsintervalls*** und des ***Schlagvolumens*** können unter Verwendung eines intravaskulären Impedanzsensors gemessen werden. Für die frequenzadaptive Stimulation wurden die Änderungen des Präejektionsintervalls und des Schlagvolumens bewertet. Für die Messungen benötigte der Sensor eine tripolare rechtsventrikuläre endokardiale Elektrode. Ein kontinuierlicher, unterschwelliger Wechselstrom mit konstanter Amplitude wurde von der Kathode zum Impulsgeneratorgehäuse geleitet. Die ersten Implantationen wurden 1989 durchgeführt (Cardiac Pacemakers, USA). In einem Bericht von *Platia* von 1990 über 16 Patienten wurde das Präejektionsintervall für die Frequenzanpassung als nützlich bewertet, obwohl ein paradoxer Abfall der Frequenz beim Einnehmen einer aufrechten Haltung auftrat^251^. Die Abhängigkeit der Frequenzanpassung von der Körperposition, unbekannte Reaktionen bei Rechtsherzerkrankungen, die Notwendigkeit einer speziellen Elektrode und der Erfolg des Aktivitätssensors verhinderten eine weitere Entwicklung.

Ein weiteres Prinzip der intrakardialen Impedanzmessung stellte in **Deutschland** *Schaldach* 1992 mit dem ***Closed-Loop-Stimulationssensor (CLS)*** (Biotronik, Deutschland) vor. Das Konzept sah vor, dass mit der Messung der Kontraktionsleistung des Myokards Informationen genutzt werden, die das autonome Nervensystem (ANS) repräsentieren und damit bei physischer und mentaler Belastung eine physiologische Frequenzadaption ermöglichen. Zur Messung wurde die Impedanz nach einer Stimulationsabgabe oder eines detektierten Ereignisses durch die Abgabe von unterschwelligen Impulsen bestimmt, um hieraus intrakardiale Impedanzkurven zu berechnen. Ein spezieller Detektionsalgorithmus erlaubte die Bestimmung der regionalen effektiven Steigungsquantität und durch die Verwendung eines individuell einstellbaren inotropen Indexes wurde die Frequenzanpassung erreicht. Das Konzept wurde in einer multizentrischen Studie anhand eines standardisierten Übungsprotokolls evaluiert^252^. Die Ergebnisse bei Patienten mit AV-Block zeigten eine gute Übereinstimmung zwischen dem spontanen Sinusrhythmus und der impedanzgesteuerten Stimulationsfrequenz. In einer Akutstudie bei 12 Patienten konnte *S. Osswald* aus **Basel**, Schweiz, zeigen, dass die Sensorsignale eng mit dem Kontraktilitätsparameter dP/dt_max_ während Dobutamin-Belastungstests korrelierten^253^. Durch den Einsatz eines zusätzlichen Akzelerometers konnten im Ruhezustand Referenzkurven erstellt werden, die im Abgleich mit den Belastungskurven zu einem automatisierten Frequenzanstieg führten. In einer Multicenterstudie mit 131 Patienten konnte *Martin Coenen* aus Basel, Schweiz, 2008 zeigen, dass der mentale Belastungstest mit einer signifikant höheren Herzfrequenz für das CLS als für den Beschleunigungssensor verbunden war. Beim 6‑Minuten-Gehtest gab es zwischen den beiden Sensoren keinen Unterschied. Die Patienten bevorzugten CLS gegenüber dem Akzelerometer^254^. Der Sensor ist in Herzschrittmacher- und Defibrillatorsystemen der Firma Biotronik, Deutschland verfügbar.

*Irnich*^255^ aus **Gießen** stellte 1988 die ***AV-Zeitverkürzung unter Belastung*** als einen physiologischen Sensor vor. Bei drei untersuchten Patienten mit AAI-Schrittmacher bestand eine reproduzierbare Korrelation zwischen Belastung und Verkürzung des AV-Delays. Dieser Effekt war bereits nach 10 s nachweisbar. Die Erhöhung der Stimulationsfrequenz über die physiologische Frequenz hinaus verursachte dagegen eine Verlängerung der AV-Überleitungszeit, so dass durch eine negative Rückkopplung eine Überstimulation vermieden konnte^256^. Weitere Patente wurden auf das Prinzip angemeldet^257^^,^ 258.

Das Konzept wurde 1998 von *Mathias Meine* vom Institut für Biomedizinische Technik der Ruhr Universität **Bochum** aufgegriffen, der 5 Probanden und 10 Herzschrittmacherpatienten mit Sinusknotenerkrankung ergometrisch untersuchte. Auf der Basis eines Regelkreises mit negativem Feedback wurde das Konzept eines dromotropen Algorithmus zur Frequenzoptimierung bei chronotrop inkompetenten SSS-Patienten mit intakter AV-Überleitung entwickelt^259^. 1999 meldete *Martin Hexamer* den Sensor zum Patent an und stellte 2004 einen neuen Algorithmus vor, mit einem erweiterten Bereich der individuellen Herzfrequenz und einer wirksamen Dämpfung der respirationsbedingten Störung. Sieben Patienten wurden unter Belastung mit diesem Algorithmus stimuliert. Die Experimente bestätigten die Eignung dieses Konzepts zur Wiederherstellung der chronotropen Kompetenz^260^. Trotz umfangreicher Untersuchungen und Patente^261^ wurde das Konzept nicht in einem implantierbaren System umgesetzt.

## His-Bündel- und Linksschenkelstimulation

Trotz jahrelanger, erfolgreicher Stimulationstherapie wird weiterhin über den optimalen ventrikulären Stimulationsort gestritten. Die in der Anfangszeit verfügbaren Herzschrittmacher lieferten zwar eine ausreichende Frequenzunterstützung, waren jedoch nicht mit der atrialen Kontraktion synchronisiert und führten so zu negativen hämodynamischen Ergebnissen, einschließlich eines erhöhten Risikos für Herzinsuffizienz und Vorhofflimmern. Diese negativen Effekte konnten durch die frequenzvariable Stimulation nicht ausgeglichen werden. Auch die vorhofsynchrone Ventrikelstimulation von der rechtsventrikulären Spitze aus verschlechterte die kontraktile Funktion bei vielen Patienten. Alternative Stimulationsorte des Myokards wie das rechtsventrikuläre Septum oder der Ausflusstrakt konnten ebenfalls eine stimulationsinduzierte Kardiomyopathie nicht verhindern^262^. Obwohl die biventrikuläre Stimulation bei Patienten mit Linksschenkelblock (LBBB) und schwerer systolischer LV-Dysfunktion die klinische Symptomatik deutlich verbessert und die Herzinsuffizienz und Sterblichkeit reduziert, bleibt ihre Rolle bei Patienten mit erhaltener systolischer LV-Funktion ungelöst (siehe Kapitel „Kardiale Resynchronisationstherapie“).

Bereits 1925 kam der Physiologe *Carl J. Wiggers* aus Cleveland, USA, zu dem Schluss, dass eine direkte Stimulation der Ventrikel einen signifikanten Effekt auf die Herzleistung hat, da die erzeugten Druckkurven sich merklich von denen unterschieden, die während einer normalen Aktivierung des Herzens beobachtet wurden^263^. Ein idealer physiologischer Stimulationsort sollte die normale Reizleitung durch das His-Purkinje-System mit einbeziehen. 1963 beschrieb *Brian Hoffmann* aus New York, USA, im chronischen Hundeversuch eine atriale endokardiale Stimulation in der Nähe des Ventrikels, bei der Vorhof und His-Bündel simultan erregt werden konnten^264^. Die erste selektive His-Bündel-Stimulation gelang *Benjamin Scherlag* aus New York, USA, 1967 bei 11 Hunden mittels einer feinen Schraubelektrode. Im Gegensatz zur direkten Stimulation der Ventrikel führte die Stimulation des His-Bündels zu keiner Veränderung in der Amplitude, Konfiguration oder Dauer der Ventrikelaktivität, die in den peripheren Elektrokardiogrammen oder intraventrikulären Elektrogrammen aufgezeichnet wurden^265^.

Eine His-Bündelstimulation beim Menschen wurde erstmals 1970 von *Onkar Narula* aus Miami, USA, beschrieben. Bei 30 Patienten konnte er zeigen, dass der QRS-Komplex in allen EKG-Ableitungen bei direkter Stimulation des His-Bündels gegenüber dem normalen Sinusrhythmus unverändert blieb, und dies auch bei einem Patienten mit komplettem AV-Block und schmalen Kammerkomplexen^266^.

Erst im Jahr 2000 beschrieb *Pramod Deshmukh* aus Sayre, USA, eine dauerhafte His-Bündel-Stimulation bei Patienten mit chronischem Vorhofflimmern und LV-Dysfunktion, die sich einer AV-Knoten-Ablation unterzogen^267^. Die Schraubelektroden wurden geleitet durch einen Elektrophysiologie-Katheter und Stylet-gesteuert im His-Bündel platziert. Dieses konnte bei 12 von 18 Patienten zuverlässig dauerhaft stimuliert werden, bei zwei dieser Patienten trat jedoch eine Sondendislokation auf. Die Reizschwellen waren mit 2,4 ± 1,0 V höher und die R‑Amplituden mit 1,0–3,2 mV niedriger als bei der konventionellen Ventrikelstimulation. Der endsystolische Diameter im Echokardiogramm nahm signifikant von 51 auf 43 mm ab und die Auswurffraktion von 20 % auf 31 % zu. Die mittlere Prozedurdauer betrug 3,7 h. Dies und eine Erfolgsrate von nur 55 % waren Gründe dafür, dass das Verfahren zunächst keine weitere Verbreiterung fand. Die His-Bündel-Stimulation blieb auch in meiner eigenen Institution (Bergmannsheil Bochum) nach 1999 auf Einzelfälle beschränkt.

Die Entwicklung einer dünnen (4,1 F) Stimulationselektrode (Medtronic SelectSecure 3830), die ohne Innenlumen über eine Einführschleuse (Medtronic SelectSite C304 und C304His mit 8,4 F und C315His mit 7F) zu platzieren ist, führte zu einer kürzeren Prozedurdauer und einer verbesserten Erfolgsrate. Mittlerweile haben auch andere Firmen (Biotronik Selectra 3D, Abbott Agilis HisPro, Boston Scientific SSPC) z. T. steuerbare Führungskatheter auf den Markt gebracht, die mit Stylet-geführten Elektroden kombiniert werden können^268^.

Es gibt nur wenige Studien, die bei Patienten mit **AV-Blockierungen** eine mögliche Auswirkung der His-Bündel-Stimulation auf die Mortalität und die Hospitalisierung wegen Herzinsuffizienz beobachtet haben. Die bisher größte ist die Fall-Kontroll-Studie von *Mohamed Abdelrahman* 2018 mit über 750 Patienten (mittlere LVEF 54 %) und einer mittleren Nachbeobachtungszeit von 2 Jahren, die entweder eine rechtsventrikuläre oder eine His-Bündel-Stimulation erhalten hatten. Der primäre Endpunkt aus Tod, Herzinsuffizienz-Hospitalisation oder Upgrade auf ein CRT-System war in der His-Gruppe niedriger als in der konventionell stimulierten Gruppe. Die Ergebnisse waren bei Patienten mit einer ventrikulären Stimulationslast von mehr als 20 % ausgeprägter, und es gab einen Trend zu reduzierter Sterblichkeit^269^.

Eine weitere Veröffentlichung von *Pugazhendhi Vijayaraman* 2018 aus derselben Gruppe untersuchte die 5‑Jahres-Nachbeobachtung bei 192 Patienten. Die Inzidenz von stimulationsinduzierter Kardiomyopathie war in der His-Gruppe signifikant geringer als in der RV-stimulierten Gruppe (2 % vs. 22 %), bei Patienten mit > 40 % ventrikulärer Stimulation war auch die Todes- oder Herzinsuffizienzrate in der RV-stimulierten Gruppe signifikant höher (HR 1,9). Dies ging zu Lasten eines größeren Bedarfs an Elektrodenrevisionen (6,7 % vs. 3 %) und Generatorwechseln (9 % vs. 1 %) in der His-Bündel-Pacing-Gruppe^270^.

In einer hämodynamischen Akutstudie zeigte *Daniel Keene* aus London 2020 im intraindividuellen Vergleich, dass die His-Bündel-Stimulation bei Patienten mit **verlängerten PR-Intervallen** eine bessere akute Herzfunktion bietet als ein Eigenrhythmus oder eine rechtsventrikuläre Stimulation. Im Gegensatz zum Stimulationsvermeidungsalgorithmus ermöglicht sie die Normalisierung verlängerter AV-Zeiten, ohne eine ventrikuläre Dyssynchronie zu verursachen^271^.

Die Daten der His-Bündel-Studien bei **AV-Knoten-Ablation** und Vorhofflimmern wurden wegen der geringen Fallzahlen in Metaanalysen zusammengefasst. Diese haben durchweg symptomatische und echokardiographische Verbesserungen gezeigt, wenn auch mit höheren Stimulationsschwellen als die rechtsventrikuläre Stimulation^272^. Die Metaanalyse von *Zhiyong Qian* aus Nanjing, China, 2018 zeigte, dass die His-Bündel-Stimulation bei Patienten mit Herzinsuffizienz und Vorhofflimmern, die sich einer AV-Knoten-Ablation unterzogen, die LVEF von 37 auf 48 % verbesserte^273^. In einer prospektiven Singlecenterstudie bei 94 Patienten mit Vorhofflimmern, Herzinsuffizienz und schmalem QRS konnte *Lan Su* aus Wenzhou, China, zeigen, dass sich die linksventrikuläre Ejektionsfraktion über eine Nachbeobachtungszeit von 3 Jahren von 45 % auf 58 % verbesserte. In der Subgruppe der Patienten mit reduzierter LVEF betrug der Ausgangswert 33 % und nahm um 20 % zu, während bei Patienten mit normaler Auswurffraktion diese sich um 8 % verbesserte^274^.

Über die **Implantationserfolgsrate** der His-Bündel-Stimulation berichtete *Keene* 2019 in einer großen internationalen Beobachtungsstudie mit insgesamt 529 Patienten. Sie lag initial bei 81 % und verbesserte sich nach Abschluss von 40 Fällen auf 87 %. Die mittlere Durchleuchtungszeit betrug 12 min und die Reizschwelle 1,4 V bei 0,8 ms Impulsdauer. Bei 7,5 % der erfolgreichen Implantationen kam es aufgrund einer Elektrodendislokation oder eines Anstiegs der Reizschwelle zu einer Deaktivierung^275^.

Eine in jüngster Zeit durch die Arbeitsgruppe um *Weijian Huang* aus Wenzhou, China, entwickelte weitere Form der Stimulation des Reizleitungssystems stellt die direkte Stimulation des linken Faszikels durch eine septale Penetration der rechtsventrikulären Elektrode dar^276^. Die Methode wird sowohl bei Patienten mit AV-Block als auch zur kardialen Resynchronisation bei verbreiterten Kammerkomplexen angewandt. Es ist noch zu früh, um den langfristigen Erfolg dieser neuen Methode zu beurteilen. Die ersten Langzeitergebnisse von *Lan Su* aus Wenzhou, China 2021, über 18 Monate bei 632 konsekutiven Patienten mit AV-Blockierungen oder AV-Knoten-Ablation und bei Patienten zur kardialen Resynchronisation sind vielversprechend. Sie zeigen eine Implantationserfolgsrate um 98 %, niedrige Reizschwellen, eine Abnahme der QRS-Breite und bei Patienten mit breitem QRS-Komplex eine Verbesserung der Auswurffraktion^277^.

Und so kann man schließen, dass auch mehr als 60 Jahre nach der Erstimplantation eines Herzschrittmachers die Entwicklung der Elektrotherapie des Herzens noch immer nicht abgeschlossen ist. Gleichzeitig unterstreicht die Aussage des 1. Patienten, *Arne Larsson* vom Dezember 2000 die Bedeutung der Therapie für den Patienten selbst:

„What was done 1958 in Stockholm, with help of doctors Senning and Elmqvist—and in a small way by myself—was a sensation. Today you don’t think of a pacemaker implantation as something sensational. Well, ladies and gentlemen, then you are wrong. It is still a sensation—for the patient!“^278^

## Footnotes

1 Lidwill, Mark C. The University of Sydney School of Medicine. Online Museum. https://www.sydney.edu.au/medicine/museum/mwmuseum/index.php/Lidwill,_Mark_C#_note-0.

2 Hyman AS. Resuscitation of the stopped heart by intracardial therapy. Experimental use of an artificial pacemaker. Arch Int Med 1932; 50:283–305.

3 Mannebach H. Hundert Jahre Herzgeschichte. Kapitel 8 Herzschrittmacher: Chardack und Senning. Springer-Verlag Berlin Heidelberg 1988; S. 62–73.

4 Callahan JC, Bigelow WO. An electrical artificial pacemaker for standstill of the heart. Ann Surg 1951; 134:8.

5 Zoll PM. Resuscitation of the heart in ventricular standstill by external electric stimulation. N Engl J Med 1952; 247:768–771.

6 Weirich WL, Paneth M, Vincent L, Lillehei CW. The treatment of complete heart block by the use of an artificial pacemaker and a myocardial electrode. Circulation Research 1958; 6:410–415.

7 Furman S. The early history of cardiac pacing. Pacing Clin Electrophysiol 2003; 26:2023–2032.

8 *Earl Elmer Bakken* (* 10. Januar 1924 in Minneapolis, USA; † 22. Oktober 2018 in Hawaii) war ein US-amerikanischer Elektrotechniker und Unternehmer. Er absolvierte an der University of Minnesota ein Studium der Elektrotechnik und erhielt 1948 den Bachelor-Grad. 1949 gründete er das Medizintechnikunternehmen Medtronic und entwickelte 1957 den ersten batteriebetriebenen Herzschrittmacher. Das kleine Unternehmen, welches zunächst aus einer Garage geführt wurde, war auf die Reparatur medizinischer Geräte spezialisiert. Es entwickelte sich zu einem internationalen Medizintechnik-Unternehmen. Bis 1976 war *Bakken* Chief Executive Officer und Chairman von Medtronic, bis 1989 noch Senior Chairman. 1994 schied er aus dem Board of Directors aus.

9 Lillehei CW, Gott VL, Hodges PC, et al. Transistor pacemaker for treatment of complete atrioventricular dissociation. JAMA 1960; 172:2006–2010.

10 Furman S, Schwedel J. An intracardiac pacemaker for Stokes-Adams Seizures. N Eng J Med 1959; 261:943–948.

11 Elmquist R, Senning A. An implantable pacemaker for the heart. In: Smyth (Ed) Medical electronics, Proc 2nd Int Conf Med Electr, Hiffe and Sons, London 1960.

12 Patent US3057356A vom 22.07.1960 (Wilson Greatbatch Technologies Inc). *Wilson Greatbatch* (*6. September 1919 in Buffalo, New York; † 27. September 2011) war ein US-amerikanischer Erfinder, der rund 325 Erfindungen zum Patent anmeldete, darunter einen implantierbaren Herzschrittmacher und eine korrosionsfreie Lithiumbatterie. Er war Fellow des IEEE und der British Royal Society of Health und einer von nur zwei Ingenieuren, die als Fellow des American College of Cardiology aufgenommen wurden. 1986 wurde er als einer von nur 63 Erfindern in die National Inventors Hall of Fame aufgenommen.

13 Chardack WM, Gage AA, Greatbatch W. A transistorized, self-contained, implantable pacemaker for the long-term correction of complete heart block. Surgery 1960; 48:643–664.

14 Zoll et al. Long-term electric stimulation of the heart for Stokes-Adams disease. Ann Surg 1961; 154:330–46.

15 Chardack et al. Correction of complete heart block by a self-contained and subcutaneously implanted pacemaker. Clinical Experience With 15 Patients. J. Thoracic and Cardiovas Surg 1961; 42:814–830.

16 Effert S, Greuel H, Grosse-Brockhoff F, Sykosch J. Die Therapie mit elektrischen Schrittmachern beim Adams-Stokes-Syndrom. Dtsch Med Wochenschr 1962; 87:473–479.

17 Effert S, Greuel H, Grosse-Brockhoff F, Sykosch J. Die Therapie mit elektrischen Schrittmachern beim Adams-Stokes-Syndrom. Dtsch Med Wochenschr 1962; 87:473–479.

18 Lüderitz B. Ingenieurwissenschaftliche Möglichkeiten und medizinische Erfordernisse. Ein autorisiertes Gespräch mit Prof. Dr. med. Sven Effert zur Entwicklung der modernen Kardiologie. CardioNews 07.01.2001.

19 Weber MF. Sven Effert (1922–2000) Leben und Werk. Dissertation. Kassel University Press GmbH, Kassel 2018. http://dx.medra.org/10.19211/KUP9783737605014.

20 Herzschrittmacher: Chirurg nach Pioniertat zunächst gefeuert. Nordwest-Zeitung Online 06.10.2011. https://www.nwzonline.de/panorama/chirurg-nach-pioniertat-zunaechst-gefeuert_a_1,0,567989647.html.

21 Sunder-Plassmann P. Pacemaker-Implantation bei totalem a.v.-Block. Thoraxchirurgie 1962; 10:220–221.

22 Sunder-Plassmann P, Portheine H, Menges G. Künstliche Schrittmacher des Herzens zur Behandlung von Adams-Stokes-Anfällen. Medizinische Klinik 1963; 58:581–586.

23 Seipel L, Pietrek G, Körfer R, Loogen F. Prognose nach Schrittmacherimplantation. Internist 1977; 18:21–24.

24 Menges G, Portheine H, Mohr HJ, Honkomp J, Esch R, Backmann L. Experimentelle und klinische Untersuchungen bei der Implantation von Herzschrittmachern. Thoraxchir vask Chir 1963/1964; 11:150–157.

25 *Max Gustav Julius Schaldach* (* 19. Juli 1936 in Berlin; † 5. Mai 2001 nahe Nürnberg tödlich verunglückt) war Physiker und Unternehmer und ein Pionier der biomedizinischen Technik in Deutschland. 1963 Gründung der Firma Biotronik, 1969 Professor für Experimentalphysik an der TU Berlin, 1970 Professor und Lehrstuhlinhaber für Physikalisch-Medizinische Technik an der Universität Erlangen-Nürnberg und geschäftsführender Direktor des 1971 gegründeten Zentralinstituts für biomedizinische Technik in Erlangen, Professor der Medizinischen Fakultät der Universität São José do Rio Preto und Professor der Biophysik an der der Lomonosov Universität Moskau, Zugehörigkeit zur Gesellschaft Deutscher Naturforscher und Ärzte. Von ihm stammen über 1500 wissenschaftliche Veröffentlichungen und über 100 Patente. B. Lüderitz: Max Schaldach (1936–2001), Z. Kardiologie, Band 90, 2001, S. 596–597.

26 Spitzer SG. Pacing and ICD Therapy in Germany. Pacing Clin Electrophysiol 1999; 22:1248–1252.

27 Flemming F, Geissler W, Schädlich M, Serfling HJ. Ein Beitrag zur chirurgischen Behandlung des Adams-Stokes-Anfalles. Z Gesamte Inn Med 1964; 19:909–915.

28 Hafemeister, G.: Elektrische Stimulierung des Herzens. Vortrag auf der 1. Tagung der Medizinisch-Wissenschaftlichen Gesellschaft der Internisten Mecklenburgs, Rostock, 23.–24.05.1963.

29 Herbst M, Ursinus W, Emmrich K, Weissbach G, Kraft D, Günther K. Die Behandlung des totalen Atrioventrikularblocks mit Adams-Stokes-Anfällen durch Einpflanzung eines elektronischen Schrittmachers. Dtsch Gesundheitsw 1964; 19:1900–1908.

30 Hafemeister G, Huth HJ, Parbs H, Schultz J. Schrittmacher-Implantation als Langzeitbehandlung von Herzrhythmusstörungen mit Adams-Stokes-Syndrom. Dtsch Gesundheitsw 1966; 21:15–22.

31 von Knorre GH, Voß W, Gänsicke FW. Vor 50 Jahren: Erste Herzschrittmacherimplantation im heutigen Mecklenburg-Vorpommern. Ärzteblatt Mecklenburg-Vorpommern 6/2013; 23:224–226.

32 Sykosch J, Büchner M, Effert S. Sechs Jahre Schrittmachertherapie. DMW 1968; 93:777–784.

33 *Werner Irnich* bekleidete eigenen Angaben zufolge als erster Diplomingenieur unter Prof. Dr. *Effert* eine Assistenzarztstelle am Klinikum der RWTH Aachen und war seit 1968 u. a. für die Betreuung von Herzschrittmacher-Patienten zuständig. Er wurde Leiter des Bioelektronischen Labors, habilitierte sich am 27. Januar 1975 als erster Ingenieurwissenschaftler an der Medizinischen Fakultät der RWTH Aachen mit der Arbeit „Elektrotherapie des Herzens“. Im Oktober 1979 übernahm er den neugegründeten Lehrstuhl für „Biomedizinische Elektronik“ im Fachbereich Medizin an der Justus-Liebig-Universität Gießen, wo er bis zu seiner Emeritierung im März 1999 wirkte. 1980 gründete er das **„Deutsche Zentralregister Herzschrittmacher“**.

34 Effert S, Irnich W. 15 Jahre Herzschrittmachertherapie. Dtsch med Wschr 1974; 99:1146–1151.

35 Dressler L, Witte J, Bohm J. 10jährige Erfahrungen und Ergebnisse in der Therapie mit künstlichen Herzschrittmachern. Dtsch Geswesen 1973; 28:1689–1693.

36 Witte J. Stand und Tendenz der Versorgung mit Herzschrittmachern in der DDR. Dtsch Geswesen 1973; 28:1686–1689.

37 Witte J et al. Die elektrische Langzeittherapie von Herzrhythmusstörungen. Übersicht über die Therapieergebnisse 1963 bis 1976 in der DDR. Dtsch Geswesen 1977; 32:2163–2170.

38 Witte J, Möbes R, Zobel W, Wohlgemuth P. Results of pacemaker therapy in the German Democratic Republic. Cor Vasa 1989; 31:98–109.

39 Pfeiffer D. Persönliche Mitteilung 03/2023.

40 Otte KB. Persönliche Mitteilung 12/2022.

41 Schwartze D. Wie ein Neurologe half, Herzpatienten zu retten. Cardio News 2015; 18:28.

42 Patente DE2225078A1 vom 23.05.1972 (Halle Laborelektronik); DE2251163A1 vom 19.10.1972 (Halle Laborelektronik); DE2321295A1 vom 27.04.1973 (Halle Ultraschalltechnik VEB); DD127430B1 vom 23.09.1976 (mit Kurt Bernd Otte); DD141999A1 vom 21.02.1979 (mit Wolfgang Hesse, Peter May, Kurt Bernd Otte, Manfred Mueller, Wolfgang Wittig); DD145497A1 vom 16.08.1979; DD152475A1 vom 13.08.1980 (mit Kurt Bernd Otte). Zusammen mit dem Physiker *Rudolf Millner* Autor des Buches „Grundlagen der medizinischen Elektrotechnik“ (Leipzig 1969, Geest & Portig).

43 Prof. Dr. sc. nat. *Kurt-Bernd Otte* war von 1971 bis 1983 maßgeblich an der Entwicklung einer eigenen Schrittmacherproduktion in der DDR beteiligt (Leiter für Forschung und Entwicklung VEB Laborelektronik Halle, entstanden durch Verstaatlichung der Privatfirma Wagomat, Fusion mit VEB Ultraschalltechnik Halle), ab 1980 Abteilungsleiter Herzreiztechnik VEB Transformatoren- und Röntgenwerke Dresden; von 1983–1990 Wissenschaftler am Institut für Polymerenchemie in Teltow; 1985 Dr. sc. nat., 1986 Ernennung zum Professor für Elektrochemie; seit 1990 Unternehmer (Otte Medizintechnik Halle, MedTec Halle, Otheka Halle, SYSTEM-DATA Beratungs- und Schulungs-GmbH Potsdam).

44 Ministerium für Gesundheitswesen.

45 Pfeiffer D. Herz-Kreislauf-Gesellschaft in der DDR. In: Lüderitz B, Arnold G (Hrsg) 75 Jahre Deutsche Gesellschaft für Kardiologie – Herz- und Kreislaufforschung. Springer-Verlag Heidelberg 2002, S. 73–117.

46 Nowak J. Schrittmacher-Witte. Porträt eines Arztes der Berliner Charité. Neues Deutschland 04./05.01.1986.

47 Witte J, Weber H, von Knorre GH, Seifert S, Mehler E, Schönherr P. Schrittmacher-System zur temporären transvenösen Herzstimulation. Dtsch Geswesen 1975; 30:305–307.

48 Der Stimulationsmodus eines Herzschrittmachers wird mit dem modifizierten NBG-Code gekennzeichnet. Der 1. Buchstabe gibt den Stimulationsort an: A (Vorhof), V (Herzkammer), D (Vorhof und Herzkammer), S (Vorhof oder Herzkammer), 0 (keiner), der 2. Buchstabe den Ort der Wahrnehmung und der 3. Buchstabe beschreibt die Betriebsart: T (getriggert), I (inhibiert), D (getriggert und inhibiert), 0 (keine). Der 4. Buchstabe hat nur noch für die Frequenzadaptation (R) eine Bedeutung. Der 5. Buchstabe, der angibt, ob an verschiedenen Stellen im Herzen stimuliert wird, ist durch die CRT Nomenklatur (CRT‑P für biventrikuläre Schrittmacher und CRT‑D für biventrikuläre Defibrillatoren) ersetzt worden. (Bernstein AD, Daubert JC, Fletcher RD, et al. The revised NASPE/BPEG generic code for antibradycardia, adaptive-rate, and multisite pacing. North American Society of Pacing and Electrophysiology/British Pacing and Electrophysiology Group. Pacing Clin Electrophysiol 2002; 25:260–264).

49 Böhme R. Der Dauerläufer. Herzschrittmacher aus Halle stellt einen Weltrekord auf: ein Vierteljahrhundert ununterbrochen im Einsatz. Mitteldeutsche Zeitung 22.12.2012.

50 Hettwer W. Mit herzlichen Grüßen … Superillu 2013; 15:22.

51 Labude-Gericke C. Ein Langläufer in der Brust. Bützower Zeitung online 25.01.2017.

52 Otte KB, von Knorre GH, Witte J, Wohlgemuth P. Herzschrittmacherentwicklung im Osten Deutschlands – ein Blick hinter die Kulissen. Cardio News 2017; 20:34.

53 Hunter SW, Roth NA, Bernardez D, et al. A bipolar myocardial electrode for complete heart block. Lancet 1959; 79:506–508.

54 Hunter SW, Roth NA. The platform bipolar myocardial electrode for prolonged treatment of total heart block. Lancet 1961; 81:115–117.

55 Aquilina O. A brief history of cardiac pacing. Images Paediatr Cardiol. 2006; 8(2): 17–81.

56 Tomaske M, Gerritse B, Kretsers L, Pretre R, Dodge-Khatami A, Rahn M, Bauersfeld U. A 12-year experience of bipolar steroid-eluting epicardial pacing leads in children. Ann Thorac Surg 2008;85;1704–1711.

57 Burger H, Kempfert J, van Linden A, Szalay Z, Schoenburg M, Walther T, Ziegelhoeffer T. Endurance and performance of two different concepts for left ventricular stimulation with bipolar epicardial leads in long-term follow-up. Thorac Cardiovasc Surg 2012;60:70–77.

58 Lagergren H, Johansson L, Landegren J, et al. One hundred cases of treatment for Adams-Stokes syndrome with permanent intravenous pacemaker. J Thorac Cardiovasc Surg 1965; 50:710–714.

59 Lagergren H, Edhag O. Long-term observation of pacemaker electrodes. Thorac cardiovasc Surgeon 1979;164–167.

60 Zucker IR, Parsonnet V, Gilbert L, Asa M. Dipolar electrode in heart block. JAMA 1963; 184:549–552.

61 Chardack WM, Gage AA, Federico AJ, Schimert G, Greatbatch W. The long-term treatment of heart block. Progress in cardiovascular diseases 1966; 9:105–135.

62 Elgiloy, Elgin Watch Co: eine Legierung aus Eisen, Nickel, Kobalt, Chrom und Molybdän.

63 MP35N, DuPont: eine Legierung aus Nickel, Kobalt, Chrom und Molybdän.

64 Alt E, Völker R, Blömer H. Lead fracture in pacemaker patients. Thorac cardiovasc Surgeon 1987; 35:101–104.

65 Stokes KB, Church T. Ten-year experience with implanted polyurethane lead insulation. Pacing Clin Electrophysiol 1986; 9:1160–1165.

66 Hayes DL, Graham KJ, Irwin M, Vidaillet H, Disler G, Sweesy M, Osborn MJ, Suman VJ, Neubauer SA, Seebandt M. A multicenter experience with a bipolar tined polyurethane ventricular lead. Pacing Clin Electrophysiol 1992;15:1033–1039.

67 Møller M, Arnsbo P, for the Danish Pacemaker Register. Appraisal of pacing lead performance from the Danish Pacemaker Register. PACE Pacing Clin Electrophysiol 1996;19:1327–1336.

68 Arnsbo P, Møller M. Updated appraisal of pacing lead performance from the Danish Pacemaker Register: the reliability of bipolar pacing leads has improved. Pacing Clin Electrophysiol 2000; 23:1401–1406.

69 Jenney C, Tan J, Karicherla A, Burke J, Helland J. A new insulation material for cardiac leads with potential for improved performance. Heart Rhythm. 2005;2:S318–S319.

70 De Voogt WG. Pacemaker Leads: Performance and Progress. Am J Cardiol 1999;83:187D–191D.

71 Bernstein AD, Parsonnet V. Survey of cardiac pacing and defibrillation in the United States in 1993. Am J Cardiol. 1996; 78:187–196.

72 Batson GA: Simplified technique for permanent transvenous endocardial pacing. Lancet 1973; 2:597–598.

73 Jahuck SJ, Gill ES, Petty AH: Permanent cardiac pacing through the subclavian vein. Br J Surg 1974; 61:373–376.

74 Sterz H, Prager H, Schellnegger W, Koller H, Haiderer O. Eine neue Methode zur perkutanen Implantation permanenter Herzschrittmacher. Wiener Medizinische Wochenschrift 1976; 126: 28–31.

75 Littleford PO, Parsonnet V, Spector SD. Method for the rapid and atraumatic insersion of permanent endocardial pacemaker electrodes through the subclavian vein. Am J Cardiol 1979; 43:980–982.

76 Kronski D, Haas H. Punktion der Vena subclavia als primärer Zugang bei einer Schrittmacher-Sondenimplantation. Herzschr Elektrophys 2001; 12:204–207.

77 Markewitz A, Nowak B, Fröhlig G, Lemke B, Wiegand U, Kolb C, Burger H. Jahresbericht 2020 des Deutschen Herzschrittmacherund Defibrillator-Registers Teil 1: Herzschrittmacher. Herzschr Elektrophys 2022; 10.1007/s00399-022-00893-5.

78 Hasan F, Nedios S, Karosiene Z, Scholten M, Lemke B, Tulka S, Knippschild S, Macher-Heidrich S, Adomeit HJ, Zarse M, Bogossian H. Perioperative complications after pacemaker implantation: higher complication rates with subclavian vein puncture than with cephalic vein cutdown. J Interv Card Electrophysiol 2022; 10.1007/s10840-022-01135-x

79 Antonelli D, Rosenfeld T, Freedberg NA, Palma E, Gross JN, Furman S. Insulation lead failure: is it a matter of insulation coating, venous approach or both? Pacing Clin Electrophysiol 1998;21:418–421.

80 Chan NY, Kwong NP, Cheong AP. Venous access and long-term pacemaker lead failure: comparing contrast-guided axillary vein puncture with subclavian puncture and cephalic cutdown. Europace 2016; 10.1093/europace/euw147

81 Benz AP, Vamos M, Erath JW, Hohnloser SH. Cephalic vs. subclavian lead implantation in cardiac implantable electronic devices: a systematic review and meta-analysis. Europace 2019; 21:121–129.

82 Atti V, Turagam MK, Garg J, Koerber S, Angirekula A, Gopinathannair R, Natale A, Lakkireddy D. Subclavian and Axillary Vein Access Versus Cephalic Vein Cutdown for Cardiac Implantable Electronic Device Implantation. A Meta-Analysis. JACC: Clinical Electrophysiology 2020; 6:661–671.

83 Stokes KB, Kay GN. Artificial Electric Cardiac Stimulation. In: Ellenbogen KA, Kay GN, Wilkoff BL (eds) Clinical Cardiac Pacing. Philadellphia, Saunders Company 1995; 3–37.

84 Furman S, Parker B, Escher DJW, Solomon N. Endocardial threshold of cardiac response as a function of electrode surface area. J Surg Res 1968; 8:161–166.

85 Furman S, Parker B, Escher DJW. Decreasing electrode size and increasing efficiency of cardiac stimulation. J Surg Res 1971; 11:105–110.

86 Irnich W. Engineering concepts of pacemaker electrodes. In: Schaldach M, Furman S (Eds) Advances in Pacemaker Technology. New York, Springer-Verlag 1975; pp 241–272.

87 Furman S, Garvey J, Hurzeler P—Pulse duration variation and electrode size as factors in pacemaker longevity. J Thorac Cardiovasc Surg 1975; 69:382–389.

88 Schaldach, M. New pacemaker electrodes. Trans Amer Soc Artif Inten Organs 1971; 17:29.

89 MacGregor DC, Wilson GJ, Lixfeld W, Pilliar RM, Bobyn JD, Silver MD, Smardon S, Miller SL. The porous-surfaced electrode. Thorac Cardiovasc Surg 1979; 78:281–291.

90 Guarda F, Galloni M, Assone F, Pasteris V, Luboz MP. Histological reactions of porous-tip endocardial electrodes implanted in sheep, Int J Artif Organs 1982; 5:267–273.

91 Mugica J, Henry L, Attuel P. Clinical experience with 910 carbon tip leads; comparison with polished platinium leads. Pacing Clin Electrophysiol 1986; 9:1230.

92 Timmis GC, Helland J, Westveer DC, Stewart J, Gordon S. The evolution of Low Threshold Leads. Clin Prog in Pacing and Electrophysiol 1983; 1:313–334.

93 Mond HG, Stokes KB. The Steroid-Eluting Electrode; A 10-Year Experience. Pacing Clin Electrophysiol 1996; 19:1016–102.

94 Schwaab B, Fröhlig G, Schwerdt H et al. Longterm follow-up of three microporous active fixation leads in atrial postion. In: Antonioli GE (Ed) Pacemaker Leads 1997. Bologna, Italy, Monduzzi 1997; 365–368.

95 Schaldach M, Bolz A, Breme J, Hubmann M, Hardt R. Acute and long-term sensing and pacing performance of pacemaker leads having titanium nitride electrode tips. In: Antonioli GE, Aubert AE, Ector H (eds) Pacemaker Leads 1991. Amsterdam, The Netherlands, Elsevier 1991, pp. 441–450.

96 Fröhlig G, Bolz A, Ströbel J, Rutz M, Lawall P, Scherdt H, Schaldach M, Schiefer H. A fractally coated, 1,3 mm^2^ high impedance pacing electrode. Pacing Clin Electrophysiol 1998; 21:1239–1246.

97 Danilovic D, Ohm OJ, Breivik K. Clinical use of low output settings in 1,2-mm2 steroid eiuting electrodes: three years of experience. Pacing Clin Electrophysiol 1998; 21:2606–2615.

98 Fröhlig G. 8.2.1 Schrittmachersonden. In: Fröhlig G, Carlson J, Jung J, Koglek W, Lemke B, Markewitz A, Neuzner J (Hrg) Herzschrittmacher- und Defibrillator-Therapie. Stuttgart, Thieme 2020; 260–267.

99 Schuder JC, Stoeckle, H. A micromodule pacemaker receiver for direct attachment to the ventricle. Trans Am Soc Artif Int Organs 1962; 8:344–351.

100 Furman S, Holcomb W, Escher DJW, Avery R. A micro-miniature radio-frequency receiver for cardiac pacing. Trans Am Soc Artif Int Organs 1966; 12:315–317.

101 Spickler JW, Rasor NS, Kedi P, Misra SN, Robins KE, LeBoeuf C. Totally self-contained intracardiac pacemaker. J. Electrocarddiology 1970; 3:325–331.

102 Vardas PE, Politopoulos C, Manios E, Parthenakis F, Tsagarkis C. A miniature pacemaker introduced intravenously and implanted endocardially. Preliminary findings from an experimental study. Eur J CPE 1991; 1: 27–30; zitiert nach: Seriwala M et al. Journal of Cardiology 2016; 67: 1–5.

103 Goto H, Sugiura T, Harada Y, Kazui T. Feasibility of using the automatic generating system for quartz watches as a leadless pacemaker power source. Med Biol Eng Comput 1999; 37:377–80.

104 EBR Systems, Inc. wurde 2003 gegründet, um ein implantierbares System zur drahtlosen Stimulation zu entwickeln, mit einem Fokus auf die Behandlung von Herzinsuffizienz mit kardialer Resynchronisationstherapie (CRT).

105 Echt DS, Cowan MW, Riley RE, Brisken AF. Feasibility and safety of a novel technology for pacing without leads. Heart Rhythm 2006; 3:1202–1206.

106 Wieneke H, Konorza T, Erbel R, Kisker E. Leadless pacing of the heart using induction technology: a feasibility study. Pacing Clin Electrophysiol 2009; 32: 177–83.

107 Reddy VY, Knops RE, Sperzel J, Miller MA, Petru J, Simon J, Sediva L, de Groot JR, Tjong FV, Jacobson P, Ostrosff A, Dukkipati SR, Koruth JS, Wilde AA, Kautzner J, Neuzil P. Permanent leadless cardiac pacing: results of the LEADLESS trial. Circulation 2014; 129:14661471.

108 Sperzel J, Burri H, Gras D, Tjong FVY, Knops RE, 4, Hindricks G, Steinwender C, Defaye P. State of the art of leadless pacing. Europace 2015; 17:1508–1513.

109 Reddy VY, Exner DV, Cantillon DJ, Doshi R, Bunch J, Tomassoni GF, Friedman PA, Estes M, John Ip, M.D., Imran Niazi, M.D., Kenneth Plunkitt, M.D., Rajesh Banker, M.D., James Porterfield, Ip JE, Dukkipati SR, for the LEADLESS II Study Investigators. Percutaneous Implantation of an Entirely Intracardiac Leadless Pacemaker. N Engl J Med 2015; 373:1125–35.

110 bei verwendeten Nominaleinstellungen (ISO-Standard) von 100 % Stimulation und 2,5 V/0,4 ms bei 60 min^−1.^

111 Sperzel J, Defaye P, Delnoy P, Garcia Guerrero JJ, Knops RE, Tondo C, Deharo JC, Wong T, Neuzil P. Primary safety results from the LEADLESS Observational Study. Europace 2018; 20: 1491–1497.

112 https://abbott.mediaroom.com/2022-04-04-Abbott-Receives-FDA-Approval-for-Aveir-TM-VR-Leadless-Pacemaker-System-to-Treat-Patients-with-Slow-Heart-Rhythms.

113 Reddy VY, Exner DV, Doshi R, Tomassoni G, Bunch TJ, Estes NAM, Neuzil P, Paulin FL, Guerrero JJG, Cantillon GJ, on behalf of the LEADLESS II Investigators. Letters. J Am Coll Cardiol Clin Elelectrophys 8:115–117.

114 Michael D. Eggen, Vladimir Grubac, and Matthew D. Bonner. Design and Evaluation of a Novel Fixation Mechanism for a Transcatheter Pacemaker. IEEE 2015; 62:2316–2323.

115 Patent US9375580B2 vom 31.10.2012 (Medtronic).

116 Ritter P, Duray GZ, Steinwender C, Soejima K, Omar R, 5, Mont L, Boersma LVA, Knops RE, Chinitz L, Zhang S, Narasimhan C, Hummel J, Lloyd M, Simmers TA, Voigt A, Laager V, Stromberg K, Bonner MD, Sheldon TJ, Reynolds D. Early performance of a miniaturized leadless cardiac pacemaker: the Micra Transcatheter Pacing Study. Eur Heart J 2015; 36:2510–2519.

117 Reynolds D, Duray GZ, Omar R, Soejima K, Neuzil P, Zhang S, Narasimhan C, Steinwender C, Brugada J, Lloyd M, Roberts PR, Sagi V, Hummel J, Bongiorni MG, Knops RE, Ellis CR, Gornick CC, Bernabei MA, Laager V, Stromberg K, Williams ER, Hudnall H, Ritter P, for the Micra Transcatheter Pacing Study Group. A Leadless Intracardiac Transcatheter Pacing System. N Engl J Med 2016;374:533–541.

118 bei Nominaleinstellung von 100 % Stimulation und 1,5 V/0,24 ms bei 60 min^−1^, bei ISO-Nenneinstellungen 4,7 Jahre. Das System verfügt aber über eine Autocapture-Funktion, die die Stimulation auf 0,5 V über der Reizschwelle optimiert.

119 Tjong FVY, Reddy VY. Permanent Leadless Cardiac Pacemaker Therapy: A Comprehensive Review. Circulation. 2017; 135:1458–1470.

120 El-Chami MF, Al-Samadi F, Clementy N, Garweg C, Martinez-Sande JL, Piccini JP, Iacopino S, Lloyd M, Prat XV, Jacobsen MD, Ritter P, Johansen JB, Tondo C, Liu F, Fagan DH, Eakley AK, Roberts PR. Updated performance of the Micra transcatheter pacemaker in the real-world setting: A comparison to the investigational study and a transvenous historical control. Heart Rhythm 2018;15: 1800–1807.

121 Steinwender C, Khelae SK, Garweg C, Yat Sun Chan J, Ritter P, Johansen JB, Sagi V, Epstein LM, Piccini JP, Pascual M, Mont L, Sheldon T, Splett V, Stromberg K, Wood N, Chinitz L. Atrioventricular Synchronous Pacing Using a Leadless Ventricular Pacemaker. Results From the MARVEL 2 Study. J Am Coll Cardiol Clin Elelectrophys 2020; 6:94–106.

122 Neugebauer F, Noti F, van Gool S, Roten L, Baldinger SH, Seiler J, Madaffari A, Servatius H, Ryser A, Tanner H, Reichlin T, Haeberlin A. Leadless atrioventricular synchronous pacing in an outpatient setting: Early lessons learned on factors affecting atrioventricular synchrony. Heart Rhythm 2022; 19:748–756.

123 Vatterott PJ, Eggen, Hilpisch KE, Drake RA, Grubac V, Anderson TA, Colin BP, Seifert KR, Mesich ML, Ramon LC. Implant, performance, and retrieval of an atrial 60 leadless pacemaker in sheep. Heart Rhythm 2021: 18:288–296.

124 Tjong FVY, Brouwer TF, Kooiman KM, Smeding L, Koop B, Soltis B, Shuros A, Wilde AAM, Burke M, Knops RE. Communicating Antitachycardia Pacing-Enabled Leadless Pacemaker and Subcutaneous Implantable Defibrillator. J Am Coll Cardiol 2016; 67:1865–1866.

125 Reddy VY, Miller MA, Neuzil P, Søgaard P, Butter C, Seifert M, Delnoy PP, van Erven L, Schalji M, Boersma LVA, Riahi S. Cardiac Resynchronization Therapy with Wireless Left Ventricular Endocardial Pacing: the SELECT-LV Study. J Am Coll Cardiol 2017; 69: 2119–2129.

126 Jeffrey K, Parsonnet V. Cardiac Pacing, 1960–1985. A Quarter Century of Medical and Industrial Innovation. Circulation 1998;97:1978–1991.

127 Furman S, Raddi WJ, Escher DJW, Denize A, Schwedel JB, Hurwitt ES. Rechargeable pacemaker for direct myocardial implantation. Arch Surg 1965; 91:796–800.

128 Silver AW, Root G, Byron FX, Sandberg H. Externally rechargeable cardiac pacemaker. Ann Thorac Surg 1965; 1:380–388.

129 Schaldach M. Bioelectric energy sources for cardiac pacing. Ann N Y Acad Sci 1969; 167:1016–1024.

130 Parsonnet V. Power Sources for Implantable Cardiac Pacemakers. Chest 1972; 61:165–173.

131 Laurens, F., A. Piwnica: Stimulateur cardiaque isotopique. Arch. Mal, Coeur 1970; 63:906.

132 Reidemeister JC, Marx E, Schulte D, Kreuzer H, Gleichmann U, Schorn A. Erste Erfahrungen mit Isotopen-Schrittmachern. Thoraxchir 1972; 20:425–440.

133 Parsonnet V, Berstein AD, Perry Y. The nuclear pacemaker: Is renewed interest warranted? Am J Cardiol 1990; 66:837–842.

134 Reidemeister JC, Schulte D, Bircks W. Clinical experience with nuclear-powered pacemakers (Plutonium-238). In: In: Schaldach M, Furman S (Eds). Advances in pacemaker technology. Springer, Berlin, Heidelberg, New York 1975, S. 485–502.

135 Chauvel C, Lavergne T, Cohen A, Ducimetière P, Le Heuzey JY, Valty J, Guize L. Radioisotpic pacemaker: Long-term clinical results. PACE 1995; 18:286–292.

136 Nuclear Regulatory Commission.

137 US Food and Drug Administration.

138 Rosenkranz KA, Schaldach M. Zur Anwendung kernenergiebetriebener Herzschrittmacher mit transvenös-endokardialer Elektrostimulation. DMW 1973; 98:2227–2233.

139 Kunst B. Eine Batterie fürs Herz. MuseumsJournal Berlin 2013:1 https://www.museumsportal-berlin.de/es/magazin/blickfange/eine-batterie-fuers-herz/.

140 Spickler JW, Rasor NS, Kedi P, Misra SN, Robins KE, LeBoeuf C. Totally self-contained intracardiac pacemaker. J. Electrocarddiology 1970; 3:325–331.

141 Meyer C. „Hurra, der ist noch da“. Spiegel 2009; 48.

142 Pfeiffer D. Persönliche Mitteilung 03/2023.

143 Bundesamt für Strahlenschutz. Herzschrittmacher mit radioaktiven Isotopenbatterien. Stand 22.08.2013 https://archive.ph/eO6AU.

144 Greatbatch W. Lee JH, Mathias W, Eldrige M, Moser JR, Schneider AA. The solid state lithium battery: A new improved chemical power source for implantable cardiac pacemakers. IEEE Trans Biomed Eng 1971; BME-18:317–324.

145 Greatbatch W, Holmes CF. The lithium/iodine battery: A historical perspective. Pacing Clin Electrophysiol 1992; 15:2034–2036.

146 Antonioli GE. Lithium pacemaker: The first clinical experience. Pacing Clin Electrophysiol 1990; 13:363–370.

147 Mond HG, Freitag G. The cardiac implantable electronic device power source: Evolution and revolution. Pacing Clin Electrophysiol 2014; 00:1–18.

148 Präuer HW, Wirtzfeld A, Lampadius M, Himmler C, Weber K. Lithium-powered cardiac pacemakers. A survey of 3 years clinical experience. Med Klin 1977; 72:1885–1891.

149 Tyers GFO, Brownlee RR. Current status of pacemaker power sources. Ann Thorac Surg. 1978; 25:571–587.

150 Chen K, Merritt DR, Howard WG, Schmidt CL, Skarstad PM. Hybrid cathode lithium batteries for implantable medical applications. J Power Sourcrs 2005; 162:837–840.

151 Bilitch M, Cosby RS, Cafferky EA. Ventricular fibrillation and competitive pacing. N Engl J Med 1967; 276:598–604.

152 Leatham A, Cook P, Davies JG. External electric stimulator for treatment of ventricular standstill. Lancet 1956; 2:1185–1189.

153 Sykosch J, Büchner M, Effert S. Sechs Jahre Schrittmachertherapie. DMW 1968; 93:777–784.

154 Sykosch J, Effert S, Pulver KG, Zacouto F. Zur Therapie mit elektrischem Schrittmacher. Ein implantierbarer, induktiv ausschaltberer elektrischer Schrittmacher. Elektromedizin 1963; 8:139–142.

155 Lemberg L, Castellanos A, Berkovits BV. Pacemaking on demand in AV block. JAMA 1965; 191:106–108.

156 Castellanos A, Lemberg L, Jude JR, Mobin-Uddin K, Berkovits BV. Implantable Demand Pacemaker. Brit Heart J 1968; 30:29–33.

157 Donato L, Denoth F. Self-synchronising cardiac pacemaker. Lancet 1966; ii:233.

158 Folkman MJ, Watkins E. An artificial conduction system for the management of complete heart block. Surg Forum 1958; 8:331–334.

159 Nathan DA, Center S, Wu C‑Y, et al. An implantable synchronous pacemaker for the long-term correction of complete heart block. Circulation 1963; 27:682–685.

160 Gilmore JD, Sarnoff SJ, Mitchell JH, Linden RJ. Synchronicity of ventricular contraction, observations comparing hemodynamic effects of atrial and ventricular pacing. Br Heart J 1963; 25:299–307.

161 Benchimol A, Duenas A, Liggett MS, et al. Contribution of atrial systole to the cardiac function at a fixed and at a variable ventricular rate. Am J Cardiol 1965; 16:11–21.

162 Samet P, Bernstein WH, Nathan DA, et al. Atrial contribution to cardiac output in complete heart block. Am J Cardiol 1965; 16:1–10.

163 Rodewald G, Giebel O, Harms H. Intravenös-intrakardiale Applikation von vorhofgesteuerten elektrischen Schrittmachern. Z Kreislauff 1964; 53:860.

164 Sykosch HJ, Effert S, Pulver KG. Synchronisierter P‑Wellen-gesteuerter Schrittmacher zur Behandlung von Adams-Stokes-Anfällen. Zentralbl f Chir 1964; 89:479–484.

165 Lagergren H. 25 years of implanted intracardiac pacers. The Lancet 1988; 19:636–638.

166 Sykosch J, Büchner M, Effert S. Sechs Jahre Schrittmachertherapie. DMW 1968; 93:777–784.

167 Lagergren H, Johansson L, Landegren J, et al. One hundred cases of treatment for Adams-Stokes syndrome with permanent intravenous pacemaker. J Thorac Cardiovasc Surg 1965; 50:710–714.

168 Kleinert M, Beer P, Taylessani A. Möglichkeiten der Elektrostimulation des Herzens mittels transmediastinal retrokardial verlegter Elektroden. Thoraxchir 1976; 24:484–492.

169 Witte J, Dressler L, Schröder G, von Knorre GH. Transvenous atrial synchronized pacing. In: Schaldach M, Furman S (Eds). Advances in pacemaker technology. Springer, Berlin, Heidelberg, New York 1975, S. 99–120.

170 Schaldach M. Fortschritte in der Schrittmachertherapie. Verh dtsch Ges Kreisl Forsch 1969; 35:268.

171 Porstmann W, Witte J, Dressler L, Schaldach M, Vogel I, Warnke H. P wave synchronous pacing using anchored atrial electrode implanted without thoracotomy. Am J Cardiol 1972; 30:74–76.

172 Wende, U., M. Schaldach: Neue intrakardiale Schrittmacherelektrode zur Vermeidung von Dislokationen bei stark dilatiertem Ventrikel. Dtsch med Wschr 1970; 95:2026.

173 Rosenkranz KA, Schaldach M. Transvenös-endokardiale Vorhofsteuerung von Schrittmachern. Dtsch Med Wschr 1971; 96:680–686.

174 Irnich W, Bleifeld W, Effert S. Permanente transvenöse Elektrostimulation des Herzens mit einer myokardial fixierten Elektrode. Thoraxchir 1972; 20:440–443.

175 Machraoui A, Schött D, Ong TS, Straub H, Jaedicke W, Barmeyer J. Erfahrungen mit 208 Schrittmacher-Schraubelektroden unter besonderer Berücksichtigung ihrer Verwendung als Vorhofelektrode. Herz 1981; 6:364–368.

176 Lemke B, Höltmann BJ, Selbach H, Barmeyer J. The atrial pacemaker: retrospective analysis of complications and life expectancy in patients with sinus node dysfunction. Int J Cardiol 1989; 22:185–193.

177 Lemke B, von Dryander S, Großkurth D, Jäger D, Machraoui A, Barmeyer J. Longterm results of actively fixed pacemaker-leads in atrial and ventricular position. In: Antonioli GE, Aubert AE, Ector H (Eds) Pacemarker leads 1991. Elsevier, Amsterdam 1991; *p* 277–282.

178 Patent DE2539553A1 vom 05.09.1975.

179 Prof. Dr.-Ing. *Peter Osypka*, promovierte als Elektroingenieur im Bereich elektrischer Unfälle/Kammerflimmern und wurde Mitbegründer der ersten deutschen Gesellschaft für Biomedizinische Technik. Nach verschiedenen Stationen, unter anderem als Post-Doctoral-Fellow an der Mayo-Clinic in Rochester, Minnesota, gründete er 1977 im südbadischen Rheinfelden die Dr. Osypka GmbH Medizintechnik – heute OSYPKA AG –, die Produkte für die invasive Erwachsenen- und Kinder-Kardiologie sowie Kinder-Herzchirurgie entwickelt und herstellt. 1986 erfand er den Hochfrequenzgenerator zur Ablation kardialer Rhythmusstörungen. 1997 gründete er die gemeinnützige Peter-Osypka-Stiftung und 2012 das „Peter-Osypka Institute for Pacing and Ablation“ an der Hochschule Offenburg. (nach: https://dgk.org/preise-und-stipendien/peter-osypka-forschungsstipendium/.

180 Dipl.-Ing. *Hans-Jürgen Bisping* arbeitete von 1969 bis 1980 als Wissenschaftlicher Mitarbeiter in der Abteilung Kardiologie des Klinikums der RWTH Aachen unter Prof. Dr. S. Effert. Er befasste sich dort mit der Elektrostimulation des Herzens und entwickelte und patentierte eine neuartige transvenöse Schraubelektrode („Bisping electrode“), die sich schnell zum heute noch gültigen Standard für die Implantation von Herzschrittmacherelektroden entwickelte. Nach 1980 folgten Stationen als Leiter Technik und Entwicklung bei der holländischen Herzschrittmacherfirma Vitatron Holding B.V. (heute eine Tochtergesellschaft der Medtronic, Inc.) sowie als Berater im europäischen Raum für die amerikanische Herzschrittmacherfirma Intermedics, Inc. (heute Teil der Boston Scientific Corporation). 1987 gründete er die Firma Bisping Medizintechnik. https://www.bispingmed.de/unternehmen/.

181 Patent DE2533766C2 29.07.1975; Patent US4106512A vom 16.12.1976 (Medtronic).

182 Kleinert M, Bisping HJ. Erste klinische Erfahrungen mit einer neuen transvenös endokardialen Schraubelektrode in Vorhof- und Kammer-Position. Z Kardiol 1977; 66:454–458.

183 Bisping HJ, Kreuzer J, Birkenheier H. Three-year clinical experience with a new endocardial screw-in lead with introduction protection for use in the atrium and ventricle. Pacing Clin Electrophysiol 1980; 3:424–435.

184 Kleinert M, Bock M, Wilhelmi F: Clinical use of a new transvenous atrial lead. Am J Cardiol 1977; 40:237–242.

185 Castillo CA, Berkovits BV, Castellanos A, Lemberg L, Callard G, Jude JR. Bifocal demand pacing. Chest 1971; 59:360–364.

186 Patent US3595242A vom 26.03.1969 (American Optical Corp).

187 Patent ZA727526B vom 26.12.1971.

188 Marx E, Sykosch HJ, Goebel H, Arnold G. Klinische und experimentelle Erfahrungen mit einem bifokalen atrioventrikulären Demand-Schrittmacher. Verhandl Dtsch Ges Kreislaufforschg 1971; 37:465–468.

189 Irnich W, de Bakker JMT. Konzept eines Optimal-Schrittmachers. Biomedizinische Technik 1975; 20:89–90.

190 Prof. Dr. *Hermann D. Funke*, Herzchirurg aus Bonn mit großer Technikleidenschaft, gehört zu den Pionieren der Medizintechnik. 1976 entwickelte er innerhalb von wenigen Tagen einen Prototyp für einen neuen Herzschrittmacher, mit dem die Stimulation von Herzvorhof und -kammer genau aufeinander abgestimmt werden konnte und der später bei Medtronic in die Serienproduktion ging. https://www.medtronic.com/de-de/c/50-jahre/geschichte.html.

191 Funke HD. Die optimierte sequentielle Stimulation von Vorhof und Kammer – ein neuartiges Therapiekonzept zur Behandlung bradykarder Dysrhythmien. Herz Kreisl 1978; 10:479–483.

192 Patent DE2701104C2 vom 12.01.1977 (Medtronic).

193 Antonioli GE. Single Lead Atrial Synchronous Ventricular Pacing: A Dream Come True. Pacing Clin Electrophysiol 1994; 17:1531–1547.

194 Nowak B, Kreuzer J, Pfeil W, Fach WA. Ergebnisse der VDD-Stimulation. Herzschr Elektrophys 2001; 12:178–185.

195 Nowak B, Voigtländer T, für die DAVE-Multicenter Studiengruppe. Aktuelle Ergebnisse der DAVE-Studie, DDD- und VDD-Evaluation: Eine prospektive randomisierte Studie zum Vergleich der DI)D- und VDD-Stimulation bei AV-Block. Herzschr Elektrophys 1998; 9:82–83.

196 Bongiorni MG, Bedendi N, Atrial stimulation by means of floating electrodes: A multicenter experience. Pacing Clin Electrophysiol 1992; 1977–1981.

197 Israel CW, Kruse IM, Van Mechelen R, Kroes G, Heynen H, Lokhoff N. Results from the use of a preshaped lead for single-pass VDD/DDD stimulation. Pacing Clin Electrophysiol 1999;22:1314–20.

198 Res JCJ, Lau C, on behalf of the European and Canadian VECATS multicenter study group. First results of the Canadian and European Single Lead DDD studies. A report of two multicenter studies on vena cava atrial stimulation (VECATS). Pacing Clin Electrophysiol 2000; 23:1804–1808.

199 Hartung D, Hartung WM, Klein HU. Bidirektionale monophasische Impulsstimulation (BiMOS) – Ein neuartiges Konzept für die atriale Floatingstimulation mit niedrigen Reizschwellen. Herzschr Elektrophys 1998; 9:70–71.

200 North American Society of Pacing and Electrophysiology.

201 Parsonnet V, Escher DJW, Furman S, Gillette PC, Goldman BS, Harthorne JW, Hauser RG, Levine PA, Maloney J. Indications for dual-chamber pacing. Pacing Clin Electrophysiol 1984;7: 318–319.

202 Markewitz A, Nowak B, Fröhlig G, Lemke B, Wiegand U, Kolb C, Burger H. Jahresbericht 2020 des Deutschen Herzschrittmacher- und Defibrillator-Register – Teil1: Herzschrittmacher. Herzschr Elektrophys 2022; 10.1007/s00399-022-00893-5.

203 Astrand I and Landegren J. The effect of varying pacemaker rate on physical work. Acta Med Scand 1965; 177:657–665.

204 Karlof I. Haemodynamic effect of atrial triggered versus fixed rate pacing at rest and during exercise in complete heart block. Acta Med Scand 1975; 197:195–206.

205 Furman S. The early history of cardiac pacing. Pacing Clin Electrophysiol 2003; 26:2023–2032.

206 Mond HG. Rate adaptive pacing: Memories from a bygone era. Heart Lung Circ 2021; 30:225–232.

207 Cammilli L, Alcidi L, Papeschi G. A new pacemaker autoregulating the rate of pacing in relation to metabolic needs. In: Watanabe Y (Ed) Cardiac pacing. Proceedings of the 5th international symposium. Excerpa Medica, Amsterdam 1977; *p* 414–419.

208 Funke HD. Ein Herzschrittmacher mit belastungsabhängiger Frequenzregulation. Biomed Tech 1975; 20:225–228.

209 Voukydis PC and Krasner JL. A physiologically regulated pacemaker. Br J Exp Pathol 1967; 48:118–123.

210 Ionescu VL. An „on demand pacemaker“ responsive to respiration rate. Pacing Clin Electrophysiol 1980; 3:375.

211 Rossi P, Plicchi G, Canducci G, Rognoni G, Aina F. Respiratory rate as a determinant of optimal pacing rate. Pacing Clin Electrophysiol 1983; 6 (Part II):502–507.

212 Rossi P, Prando MD, MagnaniA, Aina F, Rognoni G, Occhetta E. Physiological sensitivity of respiratory-dependent cardiac pacing: four year follow up. Pacing Clin Electrophysiol 1988; 11:1267–1278.

213 Patent DE3428975A1 vom 06.08.1984.

214 Lampadius MS. Event-triggered rheographic ventilation sensor for pacemaker rate control. In: Gomez FP (ed) Cardiac Pacing. Electrophysiology. Tachyarrhythmias. Editorial Grouz, Madrid 1985; p817.

215 Patent US4919136A vom 28.09.1987.

216 Alt E, Heinz M, Hirgstetter C, Emslander P, Daum S, BUlmer H (1987) Control of pacemaker rate by impedance-based respiratory minute ventilation. Chest 1987; 92:247–252.

217 Nappholz T, Lubin M, Maloney J, Simmons T. Measuring minute ventilation with a pcing catheter. Pacing Clin Electrophysiol 1985; 8:785.

218 Simmons T, Maloney J, Abi-Samra, Valenta H, Napholtz T, Castle L, Morant V. Exercise—responsive intravascular impedance changes as a rate controller for cardiac pacing. Pacing Clin Electrophysiol 1986; 9:285.

219 Patent US4702253A vom 15.10.1985 (Intermedics).

220 Mond HG, Strathmore N, Kertes P, Hunt D, Baker G. Rate responsive pacing using a minute ventilation sensor. Pacing Clin Electrophysiol 1988; 11:1866–1874.

221 Csapo G, Weisswange A, Perach W, Kannegiesser B. Autoregulation of pacemaker rate by blood temperature. Proc VIII World Congress Cardiology 1976; *p* 485.

222 Patent DE2609365A1 06.03.1976.

223 Laczkovics A, Simbrunner G, Losert U. Temperaturmessungen zur Steuerung der Herzfrequenz in der Schrittmacherchirurgie. Kongressber Österr Ges Chir 1982; 23:119.

224 Griffin JC, Jutzy KR, Claude JP, Knutti JW. Central blood temperature as a guide to optimal heart rate. Pacing Clin Electrophysiol 1983; 6:498–501.

225 Laczkovics A. The central venous blood temperature as a guide for rate control in pacemaker therapy. Pacing Clin Electrophysiol 1984; 7:822–830.

226 Alt E, Hirgstetter C, Heinz M, Wirtzfeld A. Control of pacemaker rate by central venous blood temperature (CBT). Circulation 1984; 70:11–407(A).

227 AIt E, Hirgstetter C, Heinz M, Blömer H. Rate control of physiologic pacemakers by central venous blood temperature. Circulation 1986; 73:1206–1212.

228 Patent DE2717659C2 21.04.1977 (Siemens AG).

229 Wirtzfeld A, Goedel-Meinen L, Bock T, Heinze R, Ließ HD, Munteanu J. Central venous oxygen saturation for the control of automatic rate-responsive pacing. Circulation 1981; 64:Suppl IV, 299.

230 Stangl K, Wirtzfeld A, Heinze R, Laule M, Seitz K, Göbl G. A new multisensor pacing system using stroke volume, respiratory rate, mixed venous oxygen saturation, temperature, right atrial pressure, right ventricular pressure, and dP/dt. Pacing Clin Electrophysiol 1988; 11:712–724.

231 Stangl K, Wirtzfeld A, Heinze R, Laule M. First experience with an oxygen controlled pacemaker in man. Pacing Clin Electrophysiol 1988; 11: 1882–1887.

232 Patent US4428378A 19.11.1981 (Medtronic).

233 Mond HG. Rate adaptive pacing: Memories from a bygone era. Heart Lung Circ 2021; 30:225–232.

234 Humen DP, Kostuk WJ, Klein GJ (1983). A pacemaker which automatically increases its rate with physical activity. In: Steinbach K (ed). Cardiac pacing. Steinkopff Darmstadt 1983; pp 259.

235 Lindemans FW, Rankin IR, Murtaugh R, et al. Clinical experience with an activity sensing pacemaker. Pacing Clin Electrophysiol 1986; 9:978–986.

236 Lemke B, v. Dryander S, Lawo TH, Barmeyer J. Is there an adequate rate response during marching with an activity sensing pacemaker? Pacing Clin Electrophysiol 1987; 10:1218.

237 International Congress on Rate Responsive Cardiac Pacing: Hemodynamic, Clinical and Technical Implications. October 8–10, 1987, Munich, West Germany.

238 Alt E, Matula M, Theres H, et al. The basis for activity controlled rate variable cardiac pacemakers: An analysis of mechanical forces on human body induced by exercise and environment. Pacing Clin Electrophysiol 1989; 12:1667–1680.

239 Alt E, Matula M, Thilo R, et al. A new mechanical sensor for detecting body activity and posture, suitable for rate responsive pacing. Pacing Clin Electrophysiol 1988; 11:1875–1881.

240 Bongiorni MG, Soldati E, Arena G, DeSimone L, Capucci A, Galli R, Parlapiano M, Cazzin R, Moracchini P, Leonardi C, Zardo F, Ometto R, Vingenzi M, Charles R, Makin G, Biagini A. Multicenter clinical evaluation of a new SSIR pacemaker. Pacing Clin Electrophysiol 1992;15:1798–803.

241 Faerestrand S, Ohm OJ. Clinical study of a new activity sensor for rate adaptive pacing controlled by electrical signals generated by the kinetic energy of a moving magnetic ball. Pacing Clin Electrophysiol 1994;17:1944–1949.

242 Langenfeld H, Klein A, Kirstein M, Binner L. Peak endocardial acceleration-based clinical testing of the „Best“ DDDR pacemaker: European PEA clinical investigation group. Pacing Clin Electrophysiol 1998; 21:2187–2189.

243 Chinitz L, Ritter P, Khelae SK, Iacopino S, Garweg C, Grazia-Bongiorni M, Neuzil P, Johansen JB, Mont L, Gonzalez E, Sagi V, Duray GZ, Clementy N, Sheldon T, Splett V, Stromberg K, Wood N, Steinwender C. Accelerometer-based atrioventricular synchronous pacing with a ventricular leadless pacemaker: results from the Micra atrioventricular feasibility studies. Heart Rhythm 2018;15:1363–1371.

244 Rickards AF, Norman J. Relation between QT interval and heart rate. New design of physiologically adaptive cardiac pacemaker. Br Heart J 1981; 45:56–61.

245 Bennett TD, Baudino M, Bornzin G, Anderson K, Olsen W (1985). Rate-responsive pacing using dynamic right ventricular pressure in heart-blocked dogs. JACC 5:393.

246 Sharma A, Yee R, Bennett T, Erickson M, Beck R, Sutton R, Klein G. The effects of ventricular pacing on right ventricular maximum positive dP/dt: implications for a rate-responsive pacing system based on this parameter. Pacing Clin Electrophysiol 1987; 10: 1229.

247 Sutton R, Sharma A, Ingram A, Camm J, Lindermans F, Bennett T. First derivative of right ventricular pressure as a sensor for an implantable rate responsive VVI pacemaker. Pacing Clin Electrophysiol 1987; 10:1230.

248 Wilson F, Macleod AG, Barker P. The determination and the significance of the areas of the ventricular deflections of the electrocardiogram. Am Heart J 1934; 10:46–61.

249 Callaghan F, Gamerlo J. Tarjan P. The ventricular depolarization gradient: Exercise performance of a closed-loop rate responsive pacemaker. Pacing Clin Electrophysiol 1987; 10:1212.

250 Singer I, Camm AJ, Brown R, et at. Results with Prism-CL rate modulated pacemaker. Pacing Clin Electrophysiol 1990; 13:211.

251 Platia EV, O’Donoghue S. Waclawski SH. Stroke volume and pre-ejection interval as sensors in a dual-chamber rate-modulated pacemaker. Pacing Clin Electrophysiol 1991; 14:659.

252 Schaldach M, Hutten H. Intracardiac impedance to determine sympathetic activity in rate responsive pacing. Pacing Clin Electrophysiol 1992; 15: 1778–1786.

253 Osswald S, Cron T, Grädel C, Hilti P, Lippert M, Ströbel J, Schaldach M, et al. Closed-loop stimulation using intracardiac impedance as a sensor principle: Correlation of right ventricular dP/dt_max_ and intracardiac impedance during dobutamine stress test. Pacing Clin Electrophysiol 2000; 23:1502–1508.

254 Coenen M, Malinowski K, Spitzer W, Schuchert A, Schmitz D, Anelli-Monti M, et al. Closed loop stimulation and accelerometer-based rate adaptation: Results of the PROVIDE study. Europace 2008; 10:327–333.

255 Patent EP0255899B1 24.07.1987.

256 Irnich W, Conrady J. A new principle of rate adaptive pacing in patients with sick sinus syndrome. Pacing Clin Electrophysiol 1988; 11:1823–1828.

257 Baker RG, Patent US4856524A 29.08.1988 (Intermedics).

258 Stoop G, Begemann, Patent US5144950A vom 30.08.1990.

259 Meine M, Hexamer M, Werner J, Israel CW, Lemke B, Barmeyer J. Das „dromotrope“ Schrittmacherkonzept für die frequenzadaptive Herzschrittmachertherapie. Herzschrittmacherther Elektrophysiol. 1998; 9 Suppl 1:22–24.

260 Hexamer M, Drewes C, Meine M, Kloppe A, Weckmüller J, Mügge A, Werner J. Rate-responsive pacing using the atrio-ventricular conduction time: design and test of a new algorithm. Med Biol Eng Comput 2004;42:688–697.

261 Patent DE10029677A1 vom 25.06.1999.

262 Vijayaraman P, Bordachar P, Ellenbogen KA. The continued search for physiological pacing: Where Are We Now? J Am Coll Cardiol 2017; 69:3099–114.

263 Wiggers CJ. The muscular reactions of the mammalian ventricles to artificial stimuli. Am J Physiol 1925; 73: 346–372.

264 Hoffman BF, Moore EN, Stuckey JH, Cranefield PF. Functional properties of the atrioventricular conduction system. Circulation Res 1963; 13: 308–328.

265 Scherlag BJ, Kosowsky BD, Damato AN. A technique for ventricular pacing from the His bundle in the intact heart. J Electrocardiology 1978; 11:584–587.

266 Narula OS, Scherlag BJ, Samet P. Pervenous pacing of the specialized conducting system in man: His bundle and A‑V nodal stimulation. Circulation 1970; 41:77–87.

267 Deshmukh P, Casavant D, Romanyshyn M, Anderson K. Permanent direct HB pacing: a novel approach to cardiac pacing in patients with normal His-Purkinje activation. Circulation 2000; 101: 869–77.

268 Ravi V, El Baba M, Sharma PS. His bundle pacing: Tips and tricks. Pacing Clin Electrophysiol 2021; 44:26–34.

269 Abdelrahman M, Subzposh FA, Beer D, Durr B, Naperkowski A, Sun H, Oren JW, Dandamudi G, Vijayaraman P. Clinical outcomes of His bundle pacing compared to right ventricular pacing. J Am Coll Cardiol 2018; 71:2319–2330.

270 Vijayaraman P, Naperkowski A, Subzposh FA, Abdelrahman M, Sharma PS, Oren JW, Dandamudi G, Ellenbogen KA. Permanent His-bundle pacing: long-term lead performance and clinical outcomes. Heart Rhythm. 2018; 15:696–702.

271 Keene D, Shun-Shin MJ, Arnold AD, March K, Qureshi N, Siong Ng F, Tanner M, Linton N, Lim PB, Lefroy D, Kanagaratnam P, Peters NS, Francis DP, Whinnett ZI. Within-patient comparison of His-bundle pacing, right ventricular pacing, and right ventricular pacing avoidance algorithms in patients with PR prolongation: Acute hemodynamic study J Cardiovasc Electrophysiol. 2020; 31:2964–2974.

272 Muthumala A, Vijayaraman P. Clinical outcomes of His-Purkinje conduction system pacing. Pacing Clin Electrophysiol 2020; 1–10.

273 Qian Z, Zou F, Wang Y, Qiu Y, Chen X, Jiang H, Hou X. Permanent His bundle pacing in heart failure patients: A systematic review and meta-analysis. Pacing Clin Electrophysiol 2019; 42:139–145.

274 Su L, Cai M, Wu S, Wang S, Xu T, Vijayaraman P, Huang W. Long-term performance and risk factors analysis after permanent His-bundle pacing and atrioventricular node ablation in patients with atrial fibrillation and heart failure. Europace 2020; 22:ii19–ii26.

275 Keene D, Arnold AD, Jastrzębski M, Burri H, Zweibel S, Crespo E, Chandrasekaran B, Bassi S, Joghetaei, Swift M, Moskal P, Francis DP, Foley P, Shun-Shin, Whinnett ZI. His bundle pacing, learning curve, procedure characteristics, safety, and feasibility: Insights from a large international observational study. J Cardiovasc Electrophysiol 2019;30:1984–1993.

276 Huang W, Wu S, Vijayaraman P, Su L, Chen X, Cai B, Zou J, Lan R, Fu G, Mao G, Ellenbogen KA, Whinnett IW, Tung R. Cardiac Resynchronization Therapy in Patients With Nonischemic Cardiomyopathy Using. Left Bundle Branch Pacing J Am Coll Cardiol EP 2020; 6:849–58.

277 Su L, Wang S, Wu S, Xu L, Huang Z, Chen X, Zheng R, Jiang L, Ellenbogen KA, Whinnett ZI, MD, Huang W. Long-Term Safety and Feasibility of Left Bundle Branch Pacing in a Large Single-Center Study. Circ Arrhythm Electrophysiol 2021;14:148–156.

278 Lüderitz B. 50 Jahre implantierbarer Herzschrittmacher. Ein Gespräch mit Arne Larsson auf dem Kongress „Progress in Clinical Pacing“, Rom, Dezember 2000. CardioNews 2008.

